# Iterative One-Carbon Homologation of Unmodified Carboxylic Acids

**DOI:** 10.1021/jacs.4c13630

**Published:** 2024-12-10

**Authors:** Emilie Wheatley, Heorhii Melnychenko, Mattia Silvi

**Affiliations:** § The GSK Carbon Neutral Laboratories for Sustainable Chemistry, University of Nottingham, Jubilee Campus, Nottingham NG7 2TU, United Kingdom; ‡ School of Chemistry, University of Nottingham, University Park, Nottingham NG7 2RD, United Kingdom

## Abstract

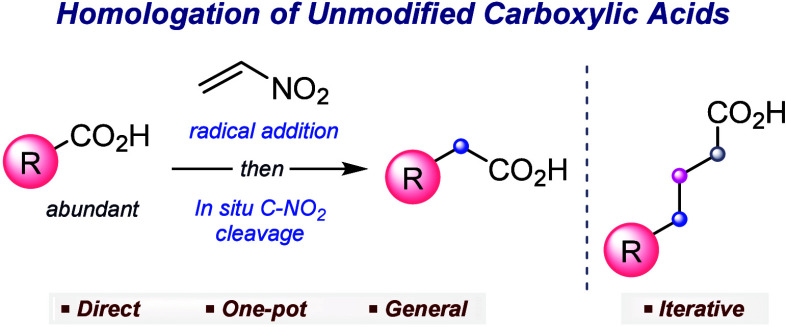

The one-carbon homologation of carboxylic acids is a valuable route to construct families of homologues, which play fundamental roles in chemistry and biology. However, known procedures are based on multistep sequences, use harsh conditions or are limited in scope. Thus, almost a century after the discovery of the original Arndt–Eistert homologation sequence, a general method to directly convert carboxylic acids into their corresponding homologues remains elusive. Exploiting the photoredox reactivity of nitroethylene, we disclose a practical visible-light-induced homologation of unmodified carboxylic acids. Iterations of the procedure reveal an exceptionally tunable strategy for the construction of inert carbon spacers, opening new opportunities in synthesis.

Methylene homologues—i.e., structural analogues differing in the length of a carbon chain—play fundamental roles in chemistry and biology.^1^ β-Amino acids **1** (Scheme 1a) represent a striking example of biologically relevant methylene homologues, constituting essential components of numerous antibiotics and peptidomimetics.^2−4^ Homologue structures also often play key roles in structure–activity relationship studies in drug design. For instance, the use of flexible carbon chain spacers is common in lead optimization, and their length is frequently observed to impact the affinity for specific targets, e.g., **3**.^5,6^

**Scheme 1:**
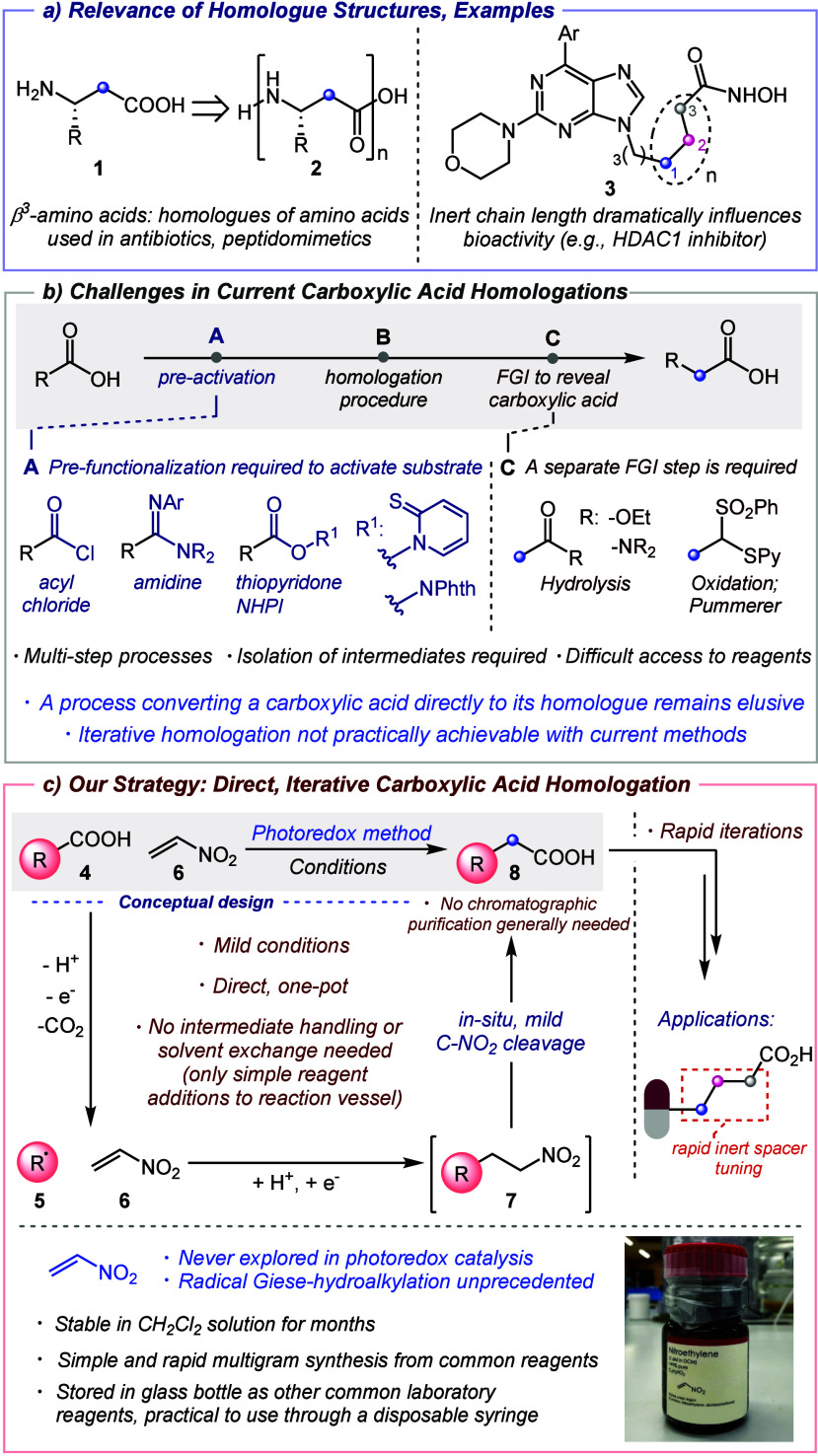
Carboxylic Acid Homologation, Relevance, Challenges, and This Strategy

The homologation reaction—i.e., the elongation of carbon chains by a single carbon unit—constitutes a fundamental strategy to access structural homologues of complex molecules.^7,8^ Given the exceptional abundance of carboxylic acids in natural products and medicinally relevant molecules,^9,10^ their homologation represents an attractive target, which has inspired significant research over the last century. The Arndt–Eistert synthesis and its variants have historically constituted the main route to homologate carboxylic acids,^11−19^ although their applicability is severely limited by drawbacks such as the use of highly reactive reagents, the multistep nature of the process, and the limited functional group tolerance. Various strategies based on classic radical chemistry,^20,21^ transition metal catalysis,^22^ and other photochemical methods^23−25^ have recently provided milder conditions for carboxylic acid homologation. Nevertheless, these procedures are often limited in scope, may require difficult-to-access reagents, are reliant on substrate preactivation, and provide products which require further chemical manipulation to reveal the desired homologated carboxylic acid (Scheme 1b). The resulting multistep sequences are cumbersome and have limited applicability, given that functional group compatibility issues often arise along the activation/deprotection line. Furthermore, the lengthy processes preclude iterative homologative synthesis, which would enable a versatile chemical canvas for the design of carbon chains with tailored length.

Thus, after almost a century from the discovery of the Arndt–Eistert synthesis,^7,8^ a direct method to homologate unmodified carboxylic acids remains elusive. Herein we outline our strategy to address this long-standing challenge, presenting a practical route for iterative one-carbon homologation that enables the practical design of carbon chains with user-defined length.

In our conceptual plan, we speculated that a photoredox decarboxylative radical generation,^26−30^ followed by addition to an appropriate radical acceptor,^31−33^ would provide a viable route for our goal. As presented in Scheme 1c, in our design plan, radical **5** would be generated upon the single-electron oxidation of carboxylic acid **4**. The open-shell species would then undergo addition to nitroethylene (**6**), a Michael acceptor which has found a relatively wide use in synthesis as an electrophile.^34−44^ Although sporadic examples of its use in radical chemistry are known, i.e., in thiohydroxamic acid ester group transfer reactions,^45−48^ its use in Giese hydroalkylation reactions is unprecedented. As a result of the planned radical addition, intermediate nitro compound **7** would be generated. We then speculated that mild conversion of this intermediate to a carboxylic acid^49^*in situ* in the reaction solution would enable direct access to the desired homologue product **8**, which ideally would be isolated through a simple acid–base extraction workup from the crude mixture. Since both the starting material **4** and its homologue product **8** bear the carboxylic acid functionality, practical iterations of our methodology would enable the tunable construction of carbon chains and application on complex substrates thanks to the mild conditions offered by photoredox catalysis.^50−54^ However, given that the use of nitroethylene is unprecedented in photocatalysis, we predicted that its high reactivity and propensity to polymerize with or without irradiation^55,56^ would constitute a significant challenge for our strategy.

Although basic reactivity and synthetic applicability studies of nitroethylene are known,^57^ information regarding its stability and storability are vague. Therefore, we commenced our investigation by performing a systematic study to assess the stability of nitroethylene (see the Supporting Information for details). Although spontaneous polymerization was observed in a variety of polar aprotic solvents and in the presence of weak bases, full stability was observed when nitroethylene was stored as a dichloromethane solution. Analogously to other common laboratory reagents, the in-house-prepared solution can be practically stored for months in a regular refrigerator (5–10 °C), with no decomposition detected, in a glass bottle kept under inert gas through a standard commercial rubber septum (Scheme 1c, bottom).

We then investigated the feasibility of our photocatalytic system (Table 1). Established iridium-,^28^ acridinium-,^58^ and cyanoarene-based^59^ photocatalytic methods (entries 1–3) led to traces or no formation of the desired nitroalkane adduct, with nitroethylene polymerization observed. This is consonant with our stability studies, which suggest nitroethylene incompatibility with the weak bases and polar solvents typically required for photoredox decarboxylative radical reactions. We therefore speculated that an acridine-based photocatalytic system relying on a proton-coupled electron transfer (PCET) radical generation from a neutral carboxylic acid,^60−64^ rather than a single-electron oxidation of its corresponding carboxylate conjugate base, would enable the radical reactivity envisioned.

**Table 1:**
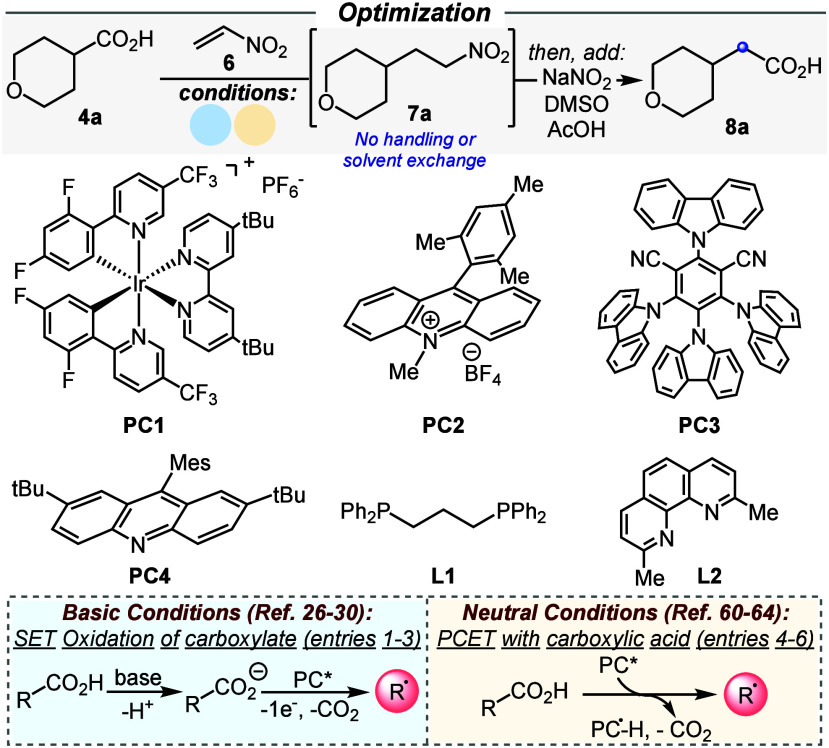
Optimization Studies

entry^a^	**PC**	base	ligand	**7a** (%)^b^	**8a** (%)^b^^,^^f^
1^c^	**PC1**	K_2_HPO_4_	–	<5	–
2^c^	**PC2**	Na_2_CO_3_	–	<5	–
3^c^	**PC3**	K_2_HPO_4_	–	0	–
4^d^	**PC4**	–	**L1**	75	–
5^d^^,^^e^	**PC4**	–	**L1**	83	–
6^d^^,^^e^	**PC4**	–	**L2**	95	95 (91)

a Reactions were carried out on a 0.1 mmol scale. Full experimental details are provided in the Supporting Information.

b ^1^H NMR yields using mesitylene as an internal standard. The value in parentheses is the yield of isolated material from a 0.2 mmol reaction.

c Reaction conditions from refs (28), (58), and (59).

d Reactions were conducted with **PC4** (10 mol %), Cu(MeCN)_4_BF_4_ (5 mol %), and **L1**–**2** (5 mol %) in CH_2_Cl_2_ (0.1 M) under 405 nm LED irradiation for 4 h.

e HFIP (10 mol %) was added.

f NaNO_2_ (6.0 equiv) and DMSO/AcOH (4:1) were added to the reaction vessel after irradiation followed by stirring at 35 °C for 24 h under open air. Mes: mesityl.

To our delight, when a methylene chloride solution of model carboxylic acid **4a** and nitroethylene **6** (1.1 equiv) was irradiated for 4 h with visible light (405 nm) in the presence of catalytic amounts of acridine **PC4**, Cu(MeCN)_4_BF_4_, and diphosphine ligand **L1**, the formation of the desired nitroalkane intermediate **7a** was observed in 75% yield (entry 4). An increase in yield to 83% was observed in the presence of 10 mol % hexafluoroisopropanol (HFIP) (entry 5), which was speculated to activate nitroethylene toward radical addition via H-bond activation^65^ and inhibit possible residual anionic polymerization decomposition pathways.^55,56^ Using neocuproine (**L2**) as a ligand, the yield of the intermediate was further increased to 95%. Addition of a 4:1 dimethyl sulfoxide/acetic acid solution of NaNO_2_^49^ to the unhandled reaction vessel just after irradiation, without any solvent evaporation or workup procedures, and stirring at 35 °C for 24 h under open air led to the quantitative conversion of intermediate **7a** to the desired carboxylic acid homologue **8a**. Remarkably, the product was isolated in 91% yield through a simple acid–base extraction workup, with no chromatographic purification involved (entry 6).

With the optimal conditions in hand, we explored the generality of our homologation strategy (Scheme 2). As per the model system, most of the substrates **4** investigated led to the desired homologated products **8** with >95% purity upon acid–base extraction workup, with silica purifications occasionally performed, mainly to remove traces of C–H or silicon grease impurities. A variety of model primary carboxylic acids undergo the homologation process in moderate to high yields (i.e., **8b**–**8i**). The process tolerates synthetically versatile alcohol or chloride handles, leading to products **8c** and **8d** in 81% and 76% yield. Terminal alkenes and alkynes can be accommodated in the substrate despite their tendency to react under radical conditions (**8e** and **8f**). Carboxylic acids bearing secondary amides, sulfones, and phosphonates can be successfully homologated, affording products **8g**–**8i**. A variety of carbocyclic as well as O- or N-heterocyclic secondary carboxylic acids undergo the desired process, with six-, five-, and even strained four-membered rings giving the desired homologues in good to excellent yields (**8j**–**8o**). Bulky tertiary carboxylic acids are equally effective substrates (e.g., adamantyl system **8p**), suggesting that steric hindrance does not hamper the desired process. A carboxylic acid bearing a cyclopropane ring successfully underwent homologation, leading to **8q** in excellent yield, corroborating the ability of particularly strained and highly reactive cyclopropyl radicals^66^ to participate to the desired process. Bicyclo[1.1.1]pentane and bicyclo[2.2.2]octane systems, valuable sp^3^-rich, nonplanar bioisosteres for aryl groups,^67−70^ undergo the homologation procedure in 47% and 70% yield, respectively (**8r** and **8s**). Bicyclic ketone **8t** was obtained in a moderate 37% yield. These results showcase the ability of highly reactive and strained bridgehead radical intermediates to undergo our desired process. The hyperlipidemia treatment drug gemfibrozil^71^ was successfully homologated to access compound **8u** in 51% yield. Lithocholic acid and oleanolic acid, respectively, primary or tertiary steroidal carboxylic acids bearing free alcohol and alkene functionalities, can be homologated to give **8v** and **8w** in 63% and 36% yield. These results further demonstrate the functional group compatibility and the generality of this homologation procedure.

**Scheme 2:**
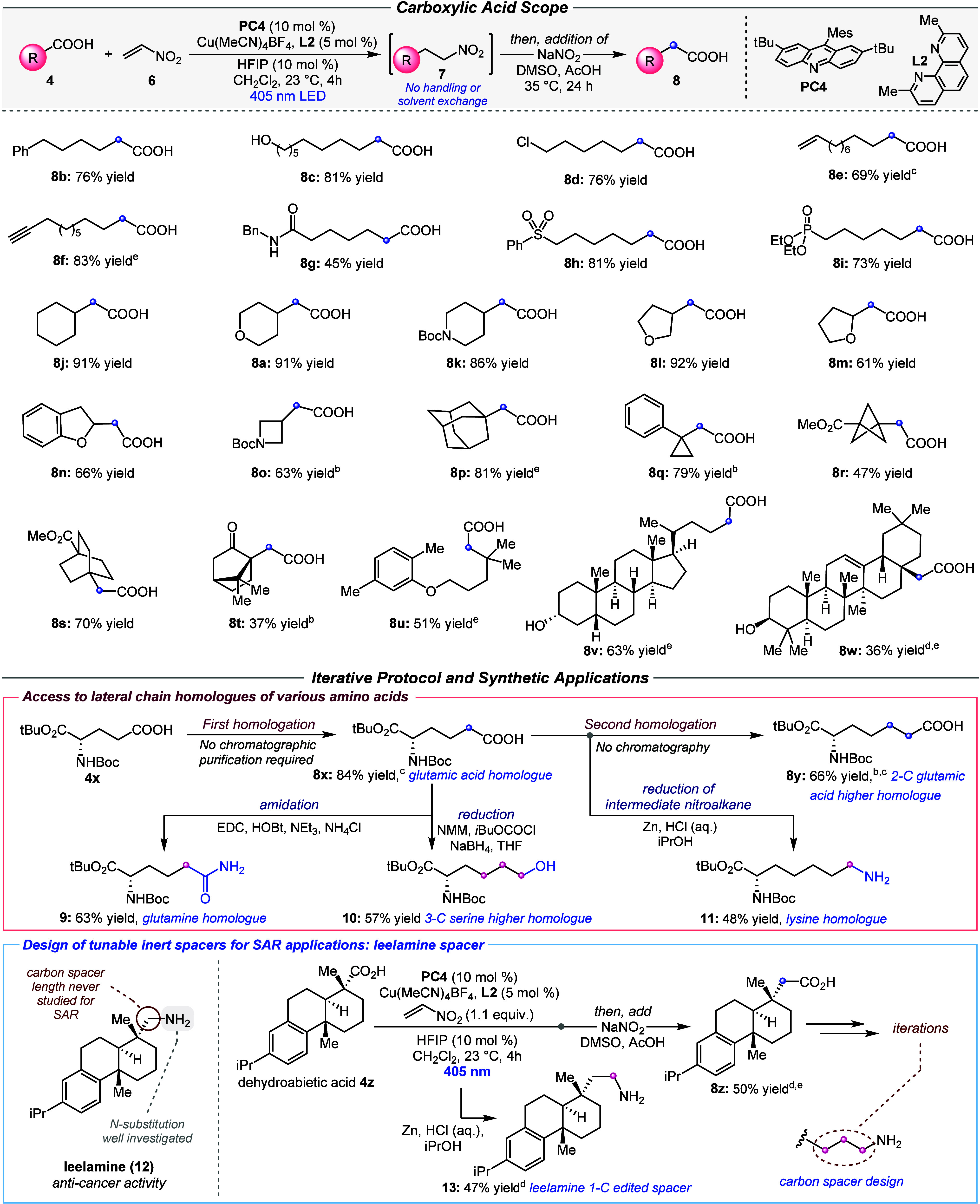
Scope and Examples of Synthetic Applications of the Carboxylic Acid Homologation Reactions were carried out on a 0.2 mmol scale with addition of NaNO_2_, DMSO, and AcOH to the reaction vessel, without further manipulations, just after irradiation. Yields refer to isolated materials. Full experimental details are provided in the Supporting Information. 15 mol % **PC4** was used. The second step was conducted for 48 h. >20:1 d.r. Aqueous acidic workup, followed by chromatographic purification. HFIP: hexafluoroisopropanol; Mes: mesityl; NMM, *N*-methylmorpholine; EDC: *N*-ethyl-*N*′-(3-(dimethylamino)propyl)carbodiimide hydrochloride; HOBt: *N*-hydroxybenzotriazole.

Next, we envisioned that practical iterations of this homologation procedure would enable the tunable construction of user-defined carbon chain spacers with various relevant synthetic applications (Scheme 2, bottom). We initially explored application in the synthesis of unnatural amino acids, valuable molecules in medicinal chemistry.^72−75^ Protected glutamic acid **4x** was homologated to its lateral chain glutamate 1-C homologue **8x** in 84% yield, with no chromatographic purifications involved in the process. Simple functional group interconversion of the pendant carboxylic acid of **8x** allowed access to other amino acid homologues, e.g., glutamine homologue **9** in 63% yield via simple amide coupling and serine 3-C higher homologue **10** via selective carboxylic acid reduction in 57% yield. An iteration of the homologation process led to glutamate 2-C homologue **8y** in 66% yield, again obtained without chromatographic purifications involved in the process. An “interrupted” version of this iteration (the intermediate nitro compound was reduced to the corresponding amine) gave lysine homologue **11** in 48% yield.

We then investigated the application of this methodology in the design of metabolically stable, tunable carbon spacers for medicinal chemistry applications. Leelamine **12** inhibits intracellular cholesterol transport and has been investigated as a potential treatment for melanoma and other types of cancer.^76−80^ Previous structure–activity relationship (SAR) studies only investigated amine derivatization,^76^ but the length of the amine spacer has never been studied. The use of traditional spacers, e.g., a glycine spacer connected via an amide bond, would introduce additional functionalities (potentially impacting target affinity and metabolic stability) and would not offer access to a fully tunable chain length. Subjecting dehydroabietic acid **4z** to the “interrupted” version of our homologation (reduction of the intermediate nitroalkane to amine) afforded leelamine homologue **13** in 47% yield. Performing the full homologation process led to the corresponding carboxylic acid homologue **8z**, which can undergo further iterations to enable the design of a highly tunable, inert carbon spacer for leelamine.

Based on previous knowledge on acridine photocatalysis^60−64^ and our mechanistic control experiments and spectroscopic investigations (see the Supporting Information), we propose the catalytic cycle shown in Scheme 3. Acridine **PC4** and substrate **4** form H-bonded complex **PC4-COOH** (detected spectroscopically; see the Supporting Information). Visible-light excitation of the complex promotes the decarboxylative generation of radical **5** and the reduced photocatalyst **PC4-H**^•^. Carbon-centered radical **5** then engages with nitroethylene **6** in the presence of Cu(I) cocatalyst **14**, leading to Cu(II)–nitronate **15**.^81^ Protonation of this species by **PC4-H**^+^—formed upon oxidation of **PC4-H**^•^ by Cu(II) species **16**—closes the catalytic cycle and affords nitroalkane **7** (generation of **16** for the first catalytic turnover is proposed to occur either by disproportionation of the Cu(I) precatalyst or by protonation of **15** by substrate **4**). Intermediate nitroalkane **7** is finally converted to the desired carboxylic acid homologue **8** upon *in situ* treatment with NaNO_2_/AcOH.^49^

**Scheme 3:**
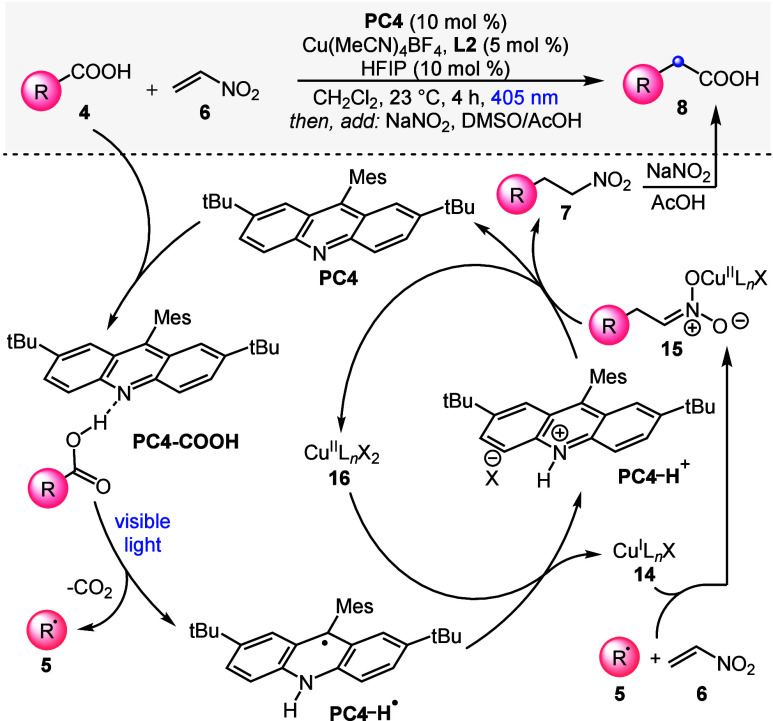
Proposed Mechanism

In conclusion, we have introduced a direct and general procedure for homologation of unmodified carboxylic acids. The methodology features a wide scope and enables late-stage homologation of complex molecules, opening the route to iterative homologation chemistry. The synthetic versatility of this methodology was demonstrated through the synthesis of a variety of unnatural amino acids and the tunable introduction of inert carbon spacers into bioactive molecules for biological chemistry applications. The homologation approach presented in this report is expected to provide a valuable tool in synthesis.

## Supporting Information Available

Spectral data for all compounds and additional experimental details, materials, and methods, including photographs of the experimental setup (PDF)
