# Supplementary material for: Iterative One-Carbon Homologation of Unmodified Carboxylic Acids
Source: J Am Chem Soc. 2024 Dec 10;146(50):34285–91. doi: 10.1021/jacs.4c13630 (PMC11664587; doi:10.1021/jacs.4c13630)

Supporting Information for

Iterative One-Carbon Homologation of Unmodified Carboxylic Acids

Emilie Wheatley[1,2†], Heorhii Melnychenko[1,2†], and Mattia Silvi[1,2*]

*Correspondence to: Mattia Silvi mattia.silvi@nottingham.ac.uk

[1] GlaxoSmithKline Carbon Neutral Laboratories for Sustainable Chemistry, University of Nottingham; Nottingham, NG7 2GA, United Kingdom.

[2] School of Chemistry, University of Nottingham; Nottingham, NG7 2RD, United Kingdom.

† These authors contributed equally to this work

Table of Contents

Materials and Methods....3

Solvents, reagents, and starting materials 3

Chromatography and instrumental analysis....3

Naming of compounds....4

LEDs and photoreactor specifications....4

Synthesis of nitroethylene....5

Supplementary Table S1. Nitroethylene stability investigation 6

Optimization studies....7

Investigating other photoredox decarboxylative methods 7

Supplementary Table S2. Optimization of the acridine photocatalytic system, investigation of photocatalysts....9

Supplementary Table S3. Investigation of the effect of ligands....10

Supplementary Table S4. Investigation of the effect of solvents 11

Supplementary Table S5. Investigation of the effect of copper sources....11

Supplementary Table S6. Control reactions 12

Supplementary Table S7. Optimization of Nef-type C-N cleavage 12

One-pot homologation reaction 12

General procedures & product characterization....13

General procedure A 13

General workup procedure (i)....13

Modified workup procedure (ii)....14

Modified workup procedure (iii)....14

Modified workup procedure (iv)....14

Supplementary Figure S1. Reaction set-up for irradiation of mixtures with 405 nm LEDs ...15

Supplementary Figure S2. Reaction set-up for Nef-type C-N bond cleavage....16

Reaction products and characterization....17

Mechanistic Insights 37

Spectroscopic investigation of the photoactive species....37

a) ^{1}H NMR studies....37

b) Control experiments with acridinium catalysts 38

c) UV/vis absorption studies....39

d) Reactivity versus wavelength studies 40
Investigation of the role of Cu/ligand cocatalyst system 41
a) Assessing the role of the copper ligand 41
b) Investigating the possible role of copper as a Lewis acid 42
c) UV-vis absorption studies to investigate the possible role of copper in the photoactivation 43
Probing the operation of a Cu(I/II) catalytic cycle 44
a) Mercury drop test 44
b) Reactivity with other Cu species 45
Proposed Catalytic Cycle 46
References and Notes 47
NMR Spectra 49

Materials and Methods

Solvents, reagents, and starting materials

All air and water-sensitive reactions were carried out in oven-dried glassware under argon atmosphere using standard Schlenk manifold technique. The solvents were degassed when needed by bubbling argon for ten minutes. Bulk solutions were evaporated under reduced pressure using a Büchi rotary evaporator. All solvents were commercially supplied or provided by the communal stills of the School of Chemistry, University of Nottingham. Commercially available compounds were purchased from Sigma Aldrich, Alfa Aesar, Acros, Fluorochem, TCI chemicals and used as received, unless otherwise stated. Dried solvents were purchased from Acros Organic, Extra Dry over molecular sieves, AcroSeal®.

Photocatalysts **PC4-7** were synthesised according to known literature procedures.[1] Compounds 7-(benzylamino)-7-oxoheptanoic acid[2], 6-(phenylsulfonyl)hexanoic acid[3], 6-(diethoxyphosphoryl)hexanoic acid[4] were synthesized following reported procedures.

Chromatography and instrumental analysis

Flash column chromatography (FCC) was carried out using Sigma-Aldrich silica gel LC60A-40 (63 μm). THF and Et_2O were distilled prior to use. All reactions were followed by thin-layer chromatography (TLC) when practical, using Merck Kieselgel 60 F_{254} fluorescent treated silica which was visualised under UV light, by staining with aqueous basic potassium permanganate, phosphomolybdic acid or with ninhydrin solution.

^{1}H-NMR and **^{13}C-NMR** spectra were recorded using Bruker broadband prodigy cryoprobe AV(III)500HD 500 MHz and Bruker AV(III)400HD 400 MHz spectrometers. Chemical shifts (δ) are given in parts per million (ppm) and coupling constants (*J*) are given in hertz (Hz). The **^{1}H-NMR** spectra are reported as follows: ppm (multiplicity, coupling constants, number of protons). High resolution mass spectra (**HRMS**) were recorded on a Bruker MicrOTOF II by Electrospray Ionisation (ESI) or on an Agilent 7890B gas chromatography system coupled with a Jeol AccuTOF GCx by Electron Ionisation (EI). **IR** spectra were recorded on a Bruker Vertex 70 FT-IR ATR as a thin film. Only selected absorption maxima (ν_{max}) are reported in wavenumbers (cm^{-1}). **Melting points** were recorded in degrees Celsius (°C), using a Stuart melting point SMP 20 microscope apparatus and are reported uncorrected. Optical rotation (**$[\alpha]_D^{25}$**) was recorded on an Anton Paar MCP 100 at 25 °C, in chloroform, with a concentration of 1g/100mL.

Naming of compounds

Compound names are generated by ChemDraw 20.0 software (PerkinElmer), following the IUPAC nomenclature.

LEDs and photoreactor specifications

Irradiation of reaction mixtures was performed using CUN0GF1A Seoul Viosys, UV LED, 405nm, 5.04W, 3.6 V LEDs purchased from RS (stock no. 247-1983). LEDs were soldered onto a primary circuit board and connected to a power supply. PCB and power supply were made by the University of Nottingham, School of Chemistry, Electronic Workshop. Power supply specifications: 1.4 A at 17 V by 2 channels. Reactions are irradiated from the bottom through the glass wall of a crystallizing basin and reactions are immersed in a temperature-controlled water-bath for rigorous control of temperature using a copper-coil connected to recirculating coolant. See Fig. S1 for visual details. The photoreactor lid and walls were custom-made using an Ultimaker S5 3D printer using Ultimaker brand polypropylene and PETG filaments.

For reactions performed at 440 nm, irradiation of reaction mixtures was performed using a Kessil lamp PR160L 440 nm LED Light 40W at 25% power. Reactions are irradiated from the side through the glass wall of a crystallizing basin and reactions are immersed in a temperature-controlled water-bath for rigorous control of temperature using an electric fan.

For reactions performed at 456 nm, irradiation of reaction mixtures was performed using LE B P0MQ-DSEQ-23 OSRAM OSTAR® Projection Power, 456 nm, 21 W, 3.80 V LEDs purchased from Mouser Electronics (ordering code Q65113A0547) at 25% power. LEDs were soldered onto a primary circuit board and connected to a power supply. PCB and power supply were made by the University of Nottingham, School of Chemistry, Electronic Workshop. Power supply specifications: 6 A at 15.2 V by 6 channels. Reactions are irradiated from the bottom through the glass wall of a crystallizing basin and reactions are immersed in a temperature-controlled water-bath for rigorous control of temperature using a copper-coil connected to recirculating coolant. See Fig. S1 for visual details. The photoreactor lid and walls were custom-made using an Ultimaker S5 3D printer using Ultimaker brand polypropylene and PETG filaments.

Synthesis of nitroethylene

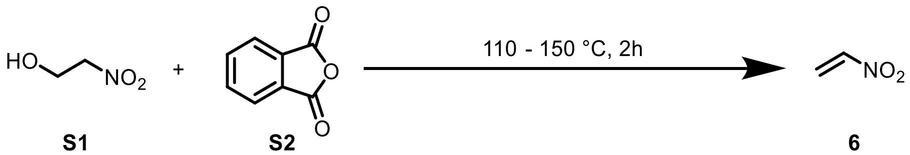

S1 (1.0 equiv.) S2 (1.3 equiv.) 6

Nitroethylene, was prepared according to a modified literature procedure.[5]

Phthalic anhydride (1.3 equiv.; 180 mmol; 26.7 g) was weighed into an oven-dried round bottom flask charged with magnetic stirrer bar. The solid was melted (~110 °C) before nitroethanol (1.0 equiv.; 140 mmol; 12.7 g) was added by syringe. The flask was then equipped with a vacuum distillation setup and the receiving flask was placed into a liquid nitrogen filled cooling bath. The apparatus was evacuated to 80 mbar and the temperature was raised to 150 °C. The pressure was reduced to 60 mbar once the temperature had been reached and the distillate was collected until distillation ceased (~2 hours) to give a pale-yellow solid in the receiving flask. The neat nitroethylene was thawed by warming the flask to room temperature and dry dichloromethane (40 mL) was added. Na_2SO_4 was added to the solution for further drying and the solution was transferred to an empty, oven-dried Acros glass bottle equipped with a new AcroSeal™ septum, under an argon atmosphere. The concentration of the nitroethylene stock solution can be measured via ^{1}H NMR analysis of an aliquot with dibromomethane as an internal standard and was found to be 1.6 M (overall 46% yield of nitroethylene). Nitroethylene as a solution in dry dichloromethane was found to be stable (no change in NMR) for at least 10 months when stored in a refrigerator.

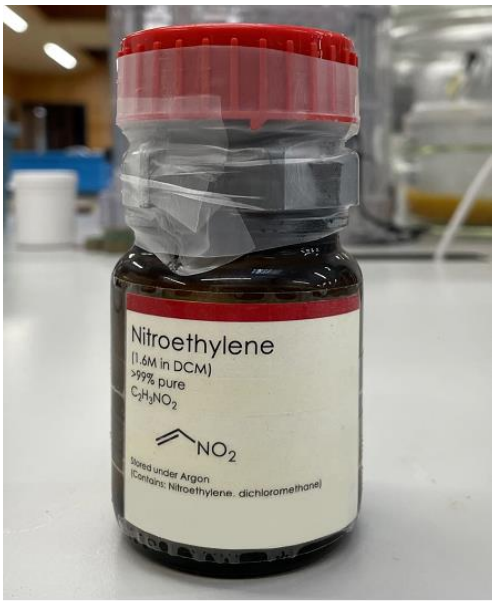

^{1}H NMR ($CDCl_3$, 500 MHz) δ (ppm): 7.17 – 7.07 (dd, J = 14.9, 7.4 Hz, 1H), 6.67 – 6.60 (dd, J = 14.8, 2.3 Hz, 1H), 5.93 – 5.86 (br d, J = 6.3 Hz, 1H); **^{13}C NMR** ($CDCl_3$, 126 MHz) δ (ppm): 145.6, 122.4. Spectral data match the ones reported.[5]

Supplementary Table S1. Nitroethylene stability investigation

Procedure for stability studies: Nitroethylene (0.2 mmol, 1.6 M in DCM) was placed in a 2 mL vial and diluted with solvent (0.1 M). The resulting solution was kept at 23 °C and the nitroethylene recovery was monitored via ^{1}H-NMR analysis of the mixture using dibromomethane as internal standard.

Entry[a]	Solvent	Base (1.0 equiv.)	Recovery of nitroethylene 6 (%)[b]	
			1h	16h
1	DMSO	-	0	-
2	DMF	-	0	-
3	MeCN	-	89	54
4	CH_2Cl_2	-	99	95
5	MeCN	K_3PO_4	53	0
6	MeCN	KF	90	8
7	MeCN	Cs_2CO_3	27	0
8	MeCN	CsF	58	0
9	MeCN	K_2HPO_4	62	0
10	MeCN	KOH	10	0
11	MeCN	2,6-lutidine	90	55
12	MeCN	Na_2HPO_4	72	12

[a] Reactions performed in 0.2 mmol scale, $[\text{nitroethylene}]_0 = 0.1$ M. [b] Unless otherwise stated, ^{1}H-NMR yield using dibromomethane as internal standard.

Optimization studies

Investigating other photoredox decarboxylative methods

a)

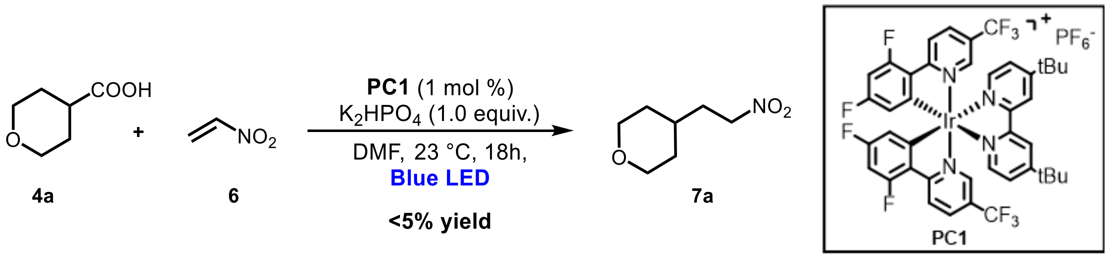

According to a literature procedure[6], carboxylic acid **4a** (1.0 equiv.; 0.1 mmol; 13mg), **PC1** (0.001 equiv.; 0.001 mmol; 1.12 mg), and K_2HPO_4 (1.0 equiv.; 0.1 mmol; 17.42 mg) were introduced into a Schlenk tube with a magnetic stirrer bar. The atmosphere was exchanged to argon and degassed, dry DMF (0.5 mL, 0.2 M, previously degassed through 10 min argon sparging) was introduced through a syringe. Nitroethylene **6** (1.1 equiv.; 0.11 mmol) was added to the reaction by microsyringe. The vessel was sealed and placed in a glass-wall water bath with cooling coil to keep the water temperature between 20 - 25 °C. The reaction was irradiated through the side glass wall with Kessil lamp A160WE Tuna Blue Saltwater LED Light 40W set to blue at maximum intensity for 18 h under moderate stirring (450 rpm). Upon completion, the reaction was passed through a celite plug and washed with DCM. The solution was concentrated by rotary evaporation.

^{1}H-NMR analysis of the crude reaction mixture using mesitylene as an internal standard revealed <5% NMR yield of product.

b)

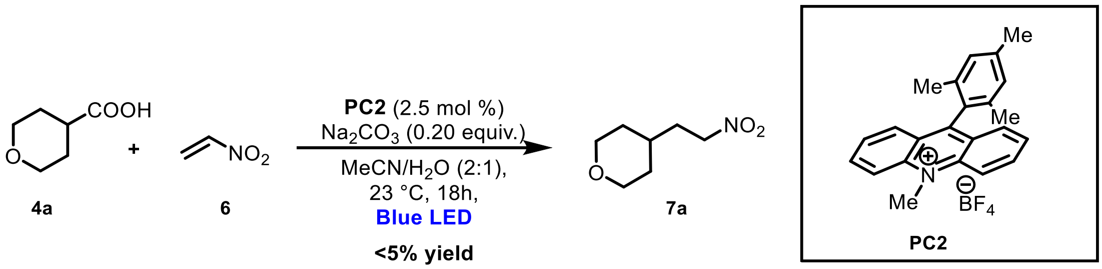

According to a literature procedure[7], carboxylic acid **4a** (1.0 equiv.; 0.1 mmol; 13mg), **PC2** (0.025 equiv.; 0.0025 mmol; 1.0 mg), and Na_2CO_3 (0.20 equiv.; 0.02 mmol; 2.12 mg) were introduced into a Schlenk tube with a magnetic stirrer bar. The atmosphere was exchanged to

argon, MeCN/H_2O (2.0 mL, 2:1, 0.2 M, previously degassed through 10 min argon sparging) was introduced through a syringe. Nitroethylene **6** (1.1 equiv.; 0.11 mmol) was added to the reaction by microsyringe. The vessel was sealed and placed in a glass-wall water bath with cooling coil to keep the water temperature between 20 - 25 °C. The reaction was irradiated through the side glass wall with Kessil lamp A160WE Tuna Blue Saltwater LED Light 40W set to blue at maximum intensity for 18 h under moderate stirring (450 rpm). Upon completion, the reaction was passed through a celite plug and washed with DCM. The solution was concentrated by rotary evaporation and the crude yield was measured using mesitylene as an internal standard.

^{1}H-NMR analysis of the crude reaction mixture using mesitylene as an internal standard revealed <5% NMR yield of product.

c)

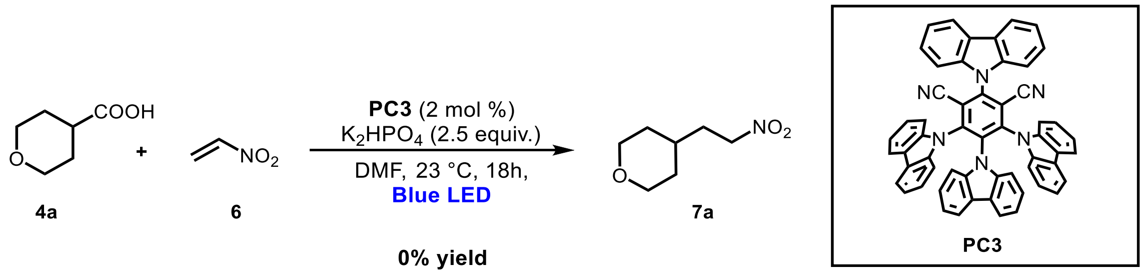

According to literature procedure[8], carboxylic acid **4a** (1.0 equiv.; 0.1 mmol; 13mg), **PC3** (0.002 equiv.; 0.002 mmol; 1.60 mg), and K_2HPO_4 (2.5 equiv.; 0.25 mmol; 43.55 mg) were introduced into a Schlenk tube with a magnetic stirrer bar. The atmosphere was exchanged to argon and dry DMF (0.5 mL, 0.2 M, previously degassed through 10 min argon sparging) was introduced through a syringe. Nitroethylene **6** (1.1 equiv.; 0.11 mmol) was added to the reaction by microsyringe. The vessel was sealed and placed in a glass-wall water bath with cooling coil to keep the water temperature between 20 - 25 °C. The reaction was irradiated through the side glass wall with Kessil lamp A160WE Tuna Blue Saltwater LED Light 40W set to blue at maximum intensity for 18 h under moderate stirring (450 rpm). Upon completion, the reaction was passed through a celite plug and washed with DCM. The solution was concentrated by rotary evaporation and the crude yield was measured using mesitylene as an internal standard.

^{1}H-NMR analysis of the crude reaction mixture using mesitylene as an internal standard revealed 0% NMR yield of product.

Supplementary Table S2. Optimization of the acridine photocatalytic system, investigation of photocatalysts

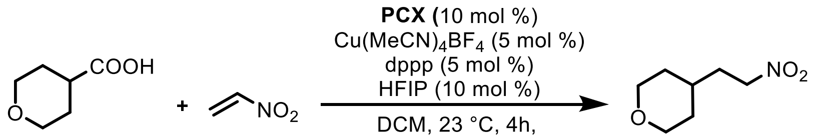

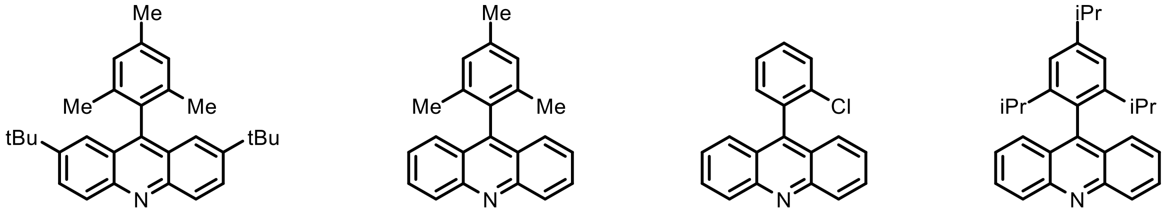

Entry[a]	Photocatalyst (PCX)	Yield 7a (%)[b]
1[c]	**PC4**	75
2	**PC4**	83
3	**PC5**	75
4	**PC6**	75
5	**PC7**	77

[a] Reactions performed in 0.1 mmol scale, using **4a** (1.0 equiv.), **6** (1.1 equiv.), [**4a**]$_0$ = 0.1 M. dppp: 1,3-Bis(diphenyl)phosphinopropane [b] Unless otherwise stated, ^{1}H-NMR yield using mesitylene as internal standard. [c]HFIP was omitted.

Supplementary Table S3. Investigation of the effect of ligands

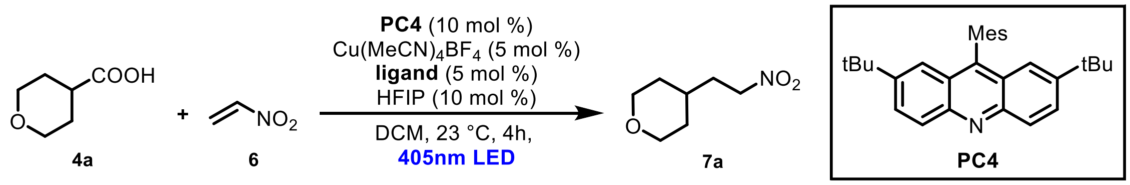

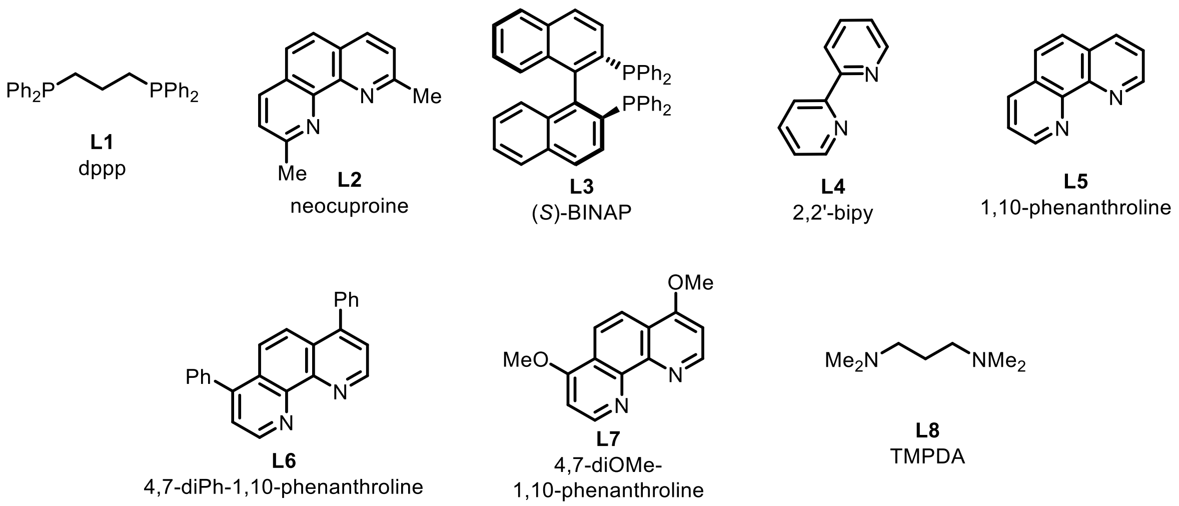

Entry[a]	Ligand	Yield 7a (%)[b]
1	**L1**	83
2	**L2**	95
3	**L3**	80
4	**L4**	89
5	**L5**	85
6	**L6**	84
7	**L7**	85
8	**L8**	83

[a] Reactions performed in 0.1 mmol scale, using **4a** (1.0 equiv.), **6** (1.1 equiv.), [**4a**]$_0$ = 0.1 M.

[b] Unless otherwise stated, ^{1}H-NMR yield using mesitylene as internal standard.

Supplementary Table S4. Investigation of the effect of solvents

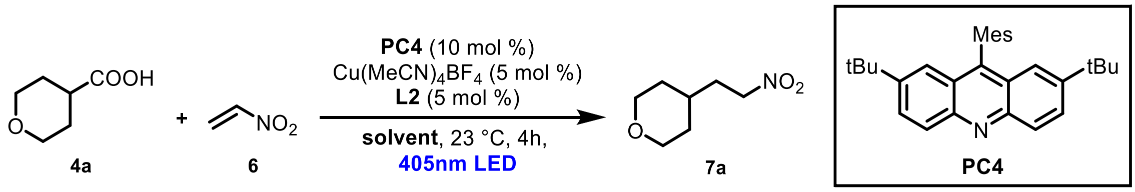

Entry[a]	Solvent	Yield 7a (%)[b]
1	**DCM**	95
2	**DCE**	86
3	**MeCN**	84
4	**EtOAc**	31
5	**Toluene**	0

[a] Reactions performed in 0.1 mmol scale, using **4a** (1.0 equiv.), **6** (1.1 equiv.), [**4a**]$_0$ = 0.1 M.

[b] Unless otherwise stated, ^{1}H-NMR yield using mesitylene as internal standard.

Supplementary Table S5. Investigation of the effect of copper sources

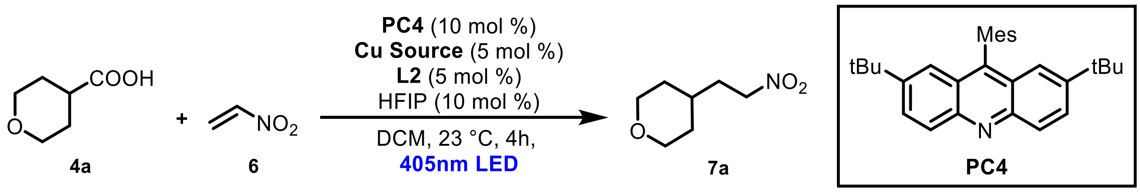

Entry[a]	Copper Source	Yield 7a (%)[b]
1	**$Cu(MeCN)_4BF_4$**	95
2	**$Cu(MeCN)_4PF_6$**	87
3	**CuI**	82
4	**Cu(TC)**	32

[a] Reactions performed in 0.1 mmol scale, using **4a** (1.0 equiv.), **6** (1.1 equiv.), [**4a**]$_0$ = 0.1 M.

[b] Unless otherwise stated, ^{1}H-NMR yield using mesitylene as internal standard.

Supplementary Table S6. Control reactions

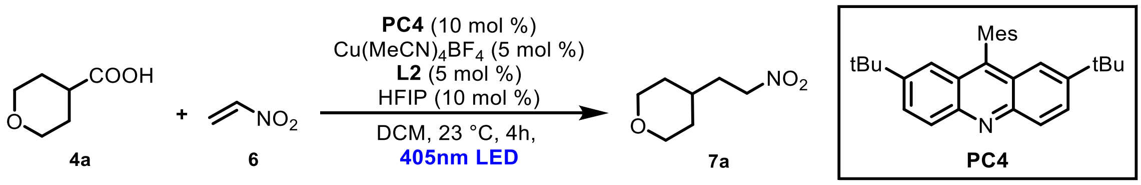

Entry[a]	Conditions	Yield 7a (%)[b]
1	**No photocatalyst**	0
2	**No Cu/L**	23
3	**Under air**	0
4	**In the dark**	0

[a] Reactions performed in 0.1 mmol scale, using **4a** (1.0 equiv.), **6** (1.1 equiv.) [**4a**]$_0$ = 0.1 M.

[b] Unless otherwise stated, ^{1}H-NMR yield using mesitylene as internal standards.

Supplementary Table S7. Optimization of Nef-type C-N cleavage

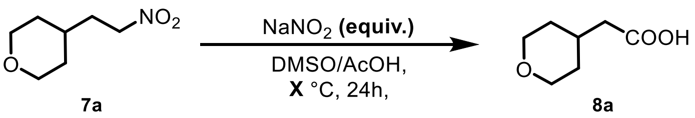

Entry[a]	$NaNO_2$ (equiv.)	DMSO/AcOH ratio	Temperature (°C)	Yield 8a (%)[b]
1	**3.0**	3:1	55	77
2	**3.0**	3:1	35	89
3	**6.0**	4:1	35	100

[a] Reactions performed in 0.1 mmol scale following the experimental procedure described in ref. 49 (maintext), using **7a** (1.0 equiv.), [**7a**]$_0$ = 0.1 M. [b] Unless otherwise stated, ^{1}H-NMR yield using mesitylene as internal standards.

One-pot homologation reaction

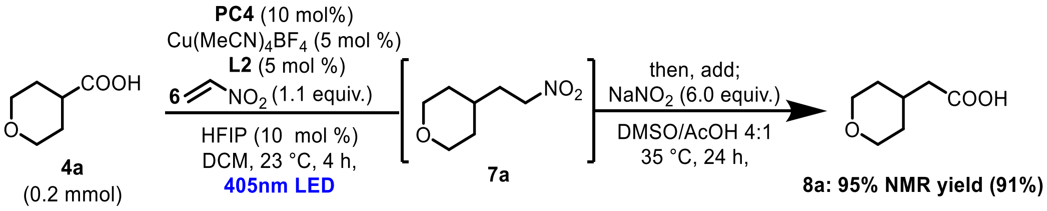

[a] Reaction performed in 0.2 mmol scale, [**4a**]$_0$ = 0.1 M. [b] ^{1}H-NMR yield using mesitylene as internal standards with yield, in parentheses yield of isolated material.

General procedures & product characterization

General procedure A

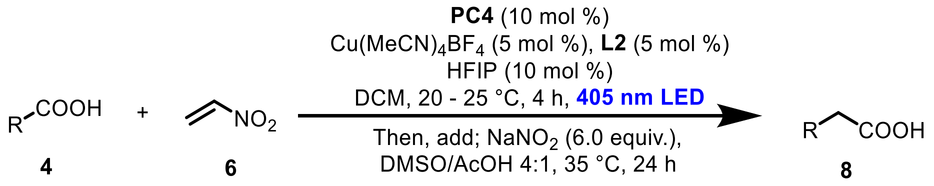

Carboxylic acid **4** (1.0 equiv.; 0.2 mmol), **PC4** (0.02 equiv.; 0.02 mmol; 8.2 mg), $Cu(MeCN)_4BF_4$ (0.01 equiv.; 0.01 mmol; 3.2 mg), and **L2** (0.01 equiv.; 0.01 mmol; 2.1 mg) were introduced into a Schlenk tube with a magnetic stirrer bar. The atmosphere was exchanged to argon and degassed, dry DCM (2.0 mL, 0.1 M, previously degassed through 10 min argon sparging) was introduced through a syringe. The reaction mixture was cooled to 0 °C (ice bath) before adding HFIP (0.02 equiv.; 0.02 mmol; 2 µL), and nitroethylene **6** (1.1 equiv.; 0.22 mmol). The vessel was sealed and placed in a glass-wall water bath with cooling coil to keep the water temperature between 20 - 25 °C. See Fig. **S1** for visual details of the reaction setup. The reaction was irradiated through the bottom glass wall with 405 nm LEDs for 4 h under moderate stirring (450 rpm). The vessel was then removed from the water bath and the cap was removed. DMSO (720 µL) and AcOH (180 µL) were added through a syringe, and $NaNO_2$ (6.0 equiv.; 1.2 mmol; 84 mg) was introduced into the vessel. The mixture was heated to 35 °C and stirred without irradiation for 24h under moderate stirring (450 rpm) with the cap off to allow gradual evaporation of dichloromethane. See Fig. **S2** for visual details.

General workup procedure (i)

Upon reaction completion, a saturated, aqueous solution of $NaHCO_3$ (10 mL) was added slowly followed by ethyl acetate (5 mL). The mixture was stirred vigorously for 1-2 minutes before transferring to a separatory funnel and extracting with saturated, aqueous $NaHCO_3$ (5 x 5 mL). The aqueous layer was then stirred with general purpose grade activated charcoal for 10 minutes and filtered through celite, rinsing with water (2 x 30 mL). The aqueous layer was then acidified with concentrated HCl and extracted with ethyl acetate (4 x 20 mL) and washed with brine (1 x 50 mL). The organic layer was dried with Na_2SO_4, filtered, and concentrated *in vacuo*. If any DMSO or AcOH are observed after workup they can be removed with further brine washes (3 x 10 mL) from ethyl acetate. If further purification was required, the crude material was subjected to chromatographic purification on silica gel to afford final compounds if required.

Modified workup procedure (ii)

Upon reaction completion, a saturated, aqueous solution of $NaHCO_3$ (10 mL) was added slowly followed by hexanes (5 mL). The mixture was stirred vigorously for 1-2 minutes before transferring to a separatory funnel and extracting with saturated, aqueous $NaHCO_3$ (5 x 5 mL). The aqueous layer was then acidified with concentrated HCl and extracted with ethyl acetate (4 x 20 mL) and washed with brine (1 x 50 mL). The organic layer was dried with Na_2SO_4, filtered, and concentrated *in vacuo*. If any DMSO or AcOH are observed after workup they can be removed with further brine washes (3 x 10 mL) from ethyl acetate. If further purification was required, the crude material was subjected to chromatographic purification on silica gel to afford final compounds if required.

Modified workup procedure (iii)

Upon reaction completion, a saturated, aqueous solution of $NaHCO_3$ (10 mL) was added slowly followed by ethyl acetate (5 mL). The mixture was stirred vigorously for 1-2 minutes before transferring to a separatory funnel and extracting with saturated, aqueous $NaHCO_3$ (5 x 5 mL). The aqueous layer was then stirred with charcoal for 10 minutes and filtered through celite, rinsing with water (2 x 30 mL). The aqueous layer was then acidified with an aqueous solution of H_2SO_4 (2M) to pH 2 and extracted with ethyl acetate (4 x 20 mL) and washed with brine (1 x 50 mL). The organic layer was dried with Na_2SO_4, filtered, and concentrated *in vacuo*. If any DMSO or AcOH are observed after workup they can be removed with further brine washes (3 x 10 mL) from ethyl acetate. If further purification was required, the crude material was subjected to chromatographic purification on silica gel to afford final compounds if required.

Modified workup procedure (iv)

Upon reaction completion, an aqueous solution of HCl (1M, 3 mL) was added followed by ethyl acetate (5 mL) and brine (5 mL). The mixture was stirred vigorously for 5 minutes before transferring to a separatory funnel and extracting with ethyl acetate (4 x 10 mL). The organic layer was dried with Na_2SO_4, filtered, and concentrated *in vacuo*. The crude material was subjected to chromatographic purification on silica gel to afford final compounds.

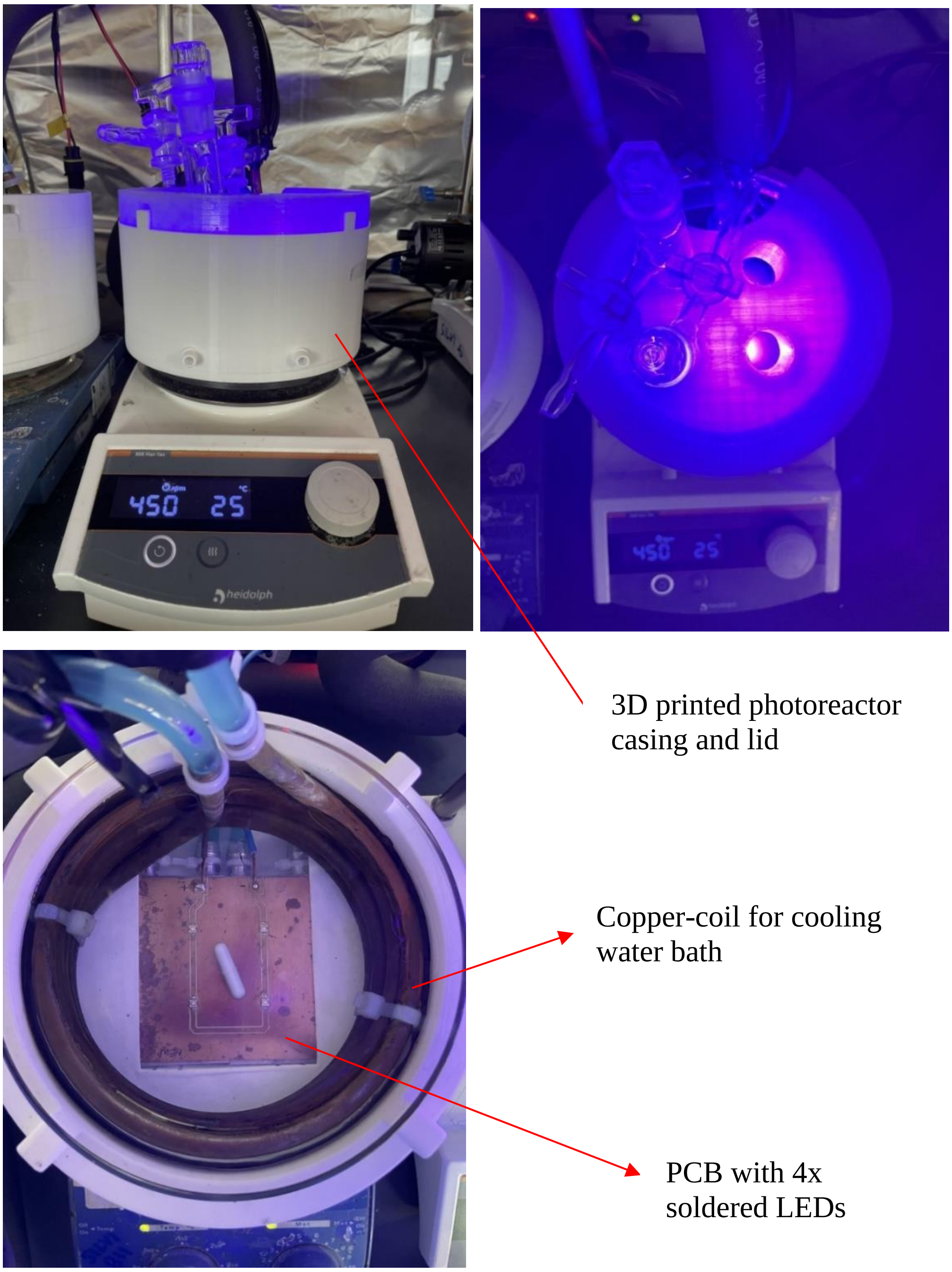

Supplementary Figure S1. Reaction set-up for irradiation of mixtures with 405 nm LEDs

Visual details of the photochemical reaction set-up: front, top, and inside photoreactor. See Materials and Methods for technical details.

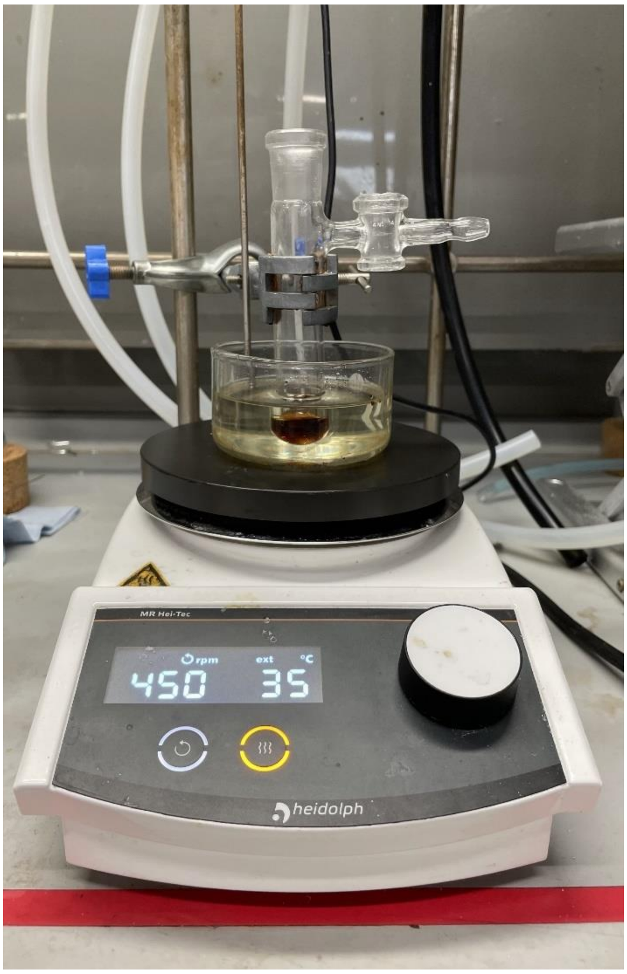

Supplementary Figure S2. Reaction set-up for Nef-type C-N bond cleavage

Visual details of the Nef-type C-N bond cleavage: After the photocatalytic step, the cap is removed from the reaction vessel and reaction is stirred for the stated time and temperature after reagents addition.

Reaction products and characterization

6-phenylhexanoic acid (8b)

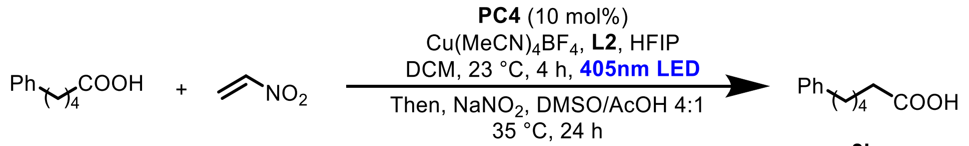

Prepared following general procedure A, using 5-phenylpentanoic acid **4b** (1.0 equiv.; 0.2 mmol; 35.6 mg), nitroethylene **6** (1.1 equiv.; 0.21 mmol), **PC4** (0.10 equiv.; 0.02 mmol; 8.2 mg), $Cu(MeCN)_4BF_4$ (0.01 equiv.; 0.01 mmol; 3.2 mg), **L2** (0.01 equiv.; 0.01 mmol; 2.1 mg), HFIP (0.02 equiv.; 0.02 mmol; 2 µL), and $NaNO_2$ (6.0 equiv.; 1.2 mmol; 84 mg). The reaction was worked up according to general workup procedure **(ii)** to afford compound **8b** (29.03 mg; 76%) as a yellow oil. **IR** (film) ν_{max}/cm^{-1}: 3027, 2933, 2858, 1700, 1496, 1454, 1412, 1285, 746, 699; **^{1}H NMR** (500 MHz, $CDCl_3$) δ (ppm): 7.34 – 7.27 (m, 2H), 7.24 – 7.17 (m, 3H), 2.68 – 2.61 (m, 2H), 2.38 (t, J = 7.5 Hz, 2H), 1.69 (ddt, J = 15.4, 13.6, 7.6 Hz, 4H), 1.47 – 1.38 (m, 2H); **^{13}C NMR** (126 MHz, $CDCl_3$) δ (ppm): 180.0, 142.6, 128.5, 128.4, 125.8, 35.8, 34.1, 31.2, 28.8, 24.7; **HRMS** (ESI-TOF) mass calculated for $[M-H]^-$ ($C_{12}H_{15}O_2^-$) expected *m/z* 191.1078; found *m/z* 191.1076.

11-Hydroxyundecanoic acid (8c)

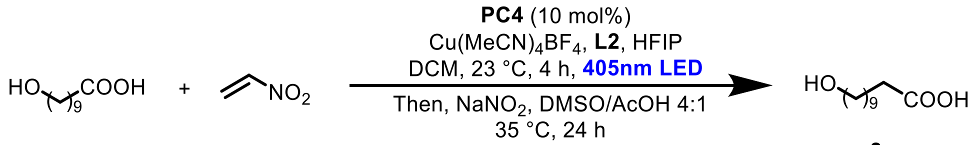

Prepared following general procedure **A**, using 10-hydroxydecanoic acid **4c** (1.0 equiv.; 0.2 mmol; 37.7 mg), nitroethylene **6** (1.1 equiv.; 0.21 mmol), **PC4** (0.10 equiv.; 0.02 mmol; 8.2 mg), $Cu(MeCN)_4BF_4$ (0.01 equiv.; 0.01 mmol; 3.2 mg), **L2** (0.01 equiv.; 0.01 mmol; 2.1 mg), HFIP (0.02 equiv.; 0.02 mmol; 2 µL), and $NaNO_2$ (6.0 equiv.; 1.2 mmol; 84 mg). The reaction was worked up according to general workup procedure **(i)** and the crude residue was purified by flash column chromatography (SiO_2; gradient 9:1:0.1 to 7:3:0.1 hexanes:EtOAc:AcOH; eluent removed using a rotary evaporator, maintaining the water bath at 35 °C and vacuum pressure 130 – 80 mbar) to afford compound **8c** (32.8 mg; 81%) as a white solid. **R_f** (8:2:0.1 hexanes:EtOAc:AcOH) = 0.09. **IR** (film) ν_{max}/cm^{-1}: 2925, 2853, 1708, 1465, 1410, 1275, 1057, 722; **^{1}H NMR** ($CDCl_3$, 500 MHz) δ (ppm): 3.64 (t, J = 6.6 Hz, 2H), 2.34 (t, J = 7.5 Hz, 2H),

1.67 – 1.53 (m, 4H), 1.40 – 1.22 (m, 12H); **^{13}C NMR** ($CDCl_3$, 126 MHz) δ (ppm): 179.5, 63.2, 34.1, 32.8, 29.6, 29.5, 29.4, 29.3, 29.1, 25.8, 24.8; **HRMS** (ESI-TOF) mass calculated for [M-H]$^-$ ($C_{11}H_{21}O_3^-$) expected *m/z* 201.1496; found *m/z* 201.1499.

5-chloropentanoic acid (**8d**)

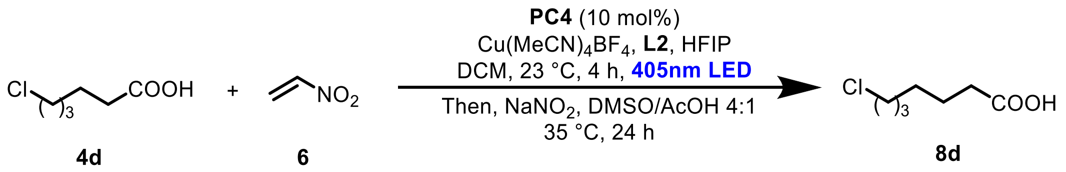

Prepared following general procedure A, using 6-chlorohexanoic acid **4d** (1.0 equiv.; 0.2 mmol; 30.1 mg), nitroethylene **6** (1.1 equiv.; 0.21 mmol), **PC4** (0.10 equiv.; 0.02 mmol; 8.2 mg), $Cu(MeCN)_4BF_4$ (0.01 equiv.; 0.01 mmol; 3.2 mg), **L2** (0.01 equiv.; 0.01 mmol; 2.1 mg), HFIP (0.02 equiv.; 0.02 mmol; 2 µL), and $NaNO_2$ (6.0 equiv.; 1.2 mmol; 84 mg). The reaction was worked up according to general workup procedure **(i)** to afford compound **8d** (24.9 mg; 76%) as a yellow oil. **IR** (film) ν_{max}/cm^{-1}: 2935, 2860, 1705, 1412, 1288, 1229, 1089, 935, 731, 651, 474. **^{1}H NMR** (400 MHz, $CDCl_3$) δ 3.55 (t, *J* = 6.7 Hz, 2H), 2.39 (t, *J* = 7.4 Hz, 2H), 1.80 (dd, *J* = 8.1, 6.6 Hz, 2H), 1.68 (p, *J* = 7.4 Hz, 2H), 1.56 – 1.34 (m, 4H); **^{13}C NMR** (101 MHz, $CDCl_3$) δ 180.17, 45.08, 34.03, 32.47, 28.4, 26.62, 24.58. **HRMS** (ESI-TOF) mass calculated for [2M-2H+Na]$^-$ ($C_{14}H_{24}Cl_2O_4Na^-$) expected *m/z* 349.0955; found *m/z* 349.0946.

dodec-11-enoic acid (**8e**)

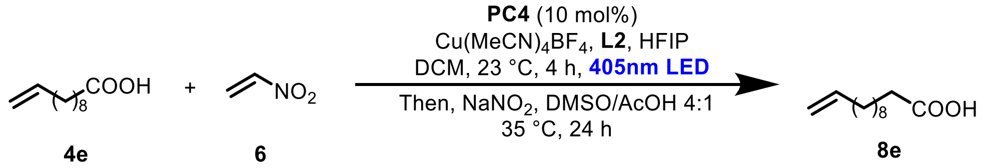

Prepared following general procedure A, using undec-10-enoic acid **4e** (1.0 equiv.; 0.2 mmol; 37.7 mg), nitroethylene **6** (1.1 equiv.; 0.21 mmol), **PC4** (0.10 equiv.; 0.02 mmol; 8.2 mg), $Cu(MeCN)_4BF_4$ (0.01 equiv.; 0.01 mmol; 3.2 mg), **L2** (0.01 equiv.; 0.01 mmol; 2.1 mg), HFIP (0.02 equiv.; 0.02 mmol; 2 µL), and $NaNO_2$ (6.0 equiv.; 1.2 mmol; 84 mg). The reaction was worked up according to general workup procedure **(ii)** and the crude residue was purified by flash column chromatography (SiO_2; gradient 9:1:0.1 to 7:3:0.1 hexanes:EtOAc:AcOH; eluent removed using a rotary evaporator, maintaining the water bath at 35 °C and vacuum pressure 130 – 80 mbar) to afford compound **8e** (27.4 mg; 69%) as a colourless oil. **R_f** (9:1:0.1 hexanes:EtOAc:AcOH) = 0.21. **IR** (film) ν_{max}/cm^{-1}: 2927, 2856, 2369, 2356, 2342, 2175, 2108, 2035, 1740, 1710, 1460, 1426, 908; **^{1}H NMR** ($CDCl_3$, 500 MHz) δ (ppm): 5.87 – 5.72 (ddt, J

= 16.9, 10.2, 6.6 Hz, 1H), 5.03 – 4.89 (m, 2H), 2.38 – 2.31 (t, J = 7.5 Hz, 2H), 2.08 – 1.99 (m, 2H), 1.66 – 1.58 (p, J = 7.4 Hz, 2H), 1.42 – 1.24 (m, 14H); **^{13}C NMR** ($CDCl_3$, 126 MHz) δ (ppm): 179.9, 139.4, 114.3, 34.1, 34.0, 29.6, 29.5, 29.4, 29.24, 29.19, 29.1, 24.8; **HRMS** (ESI-TOF) mass calculated for $[M-H]^-$ ($C_{12}H_{21}O_2^-$) expected *m/z* 197.1547; found *m/z* 197.1542.

dodec-11-ynoic acid (**8f**)

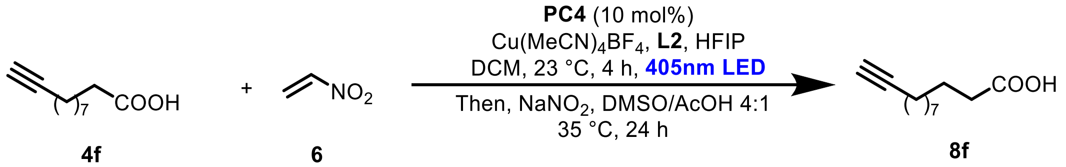

Prepared following general procedure A, using 6-chlorohexanoic acid **4f** (1.0 equiv.; 0.2 mmol; 36.4 mg), nitroethylene **6** (1.1 equiv.; 0.21 mmol), **PC4** (0.10 equiv.; 0.02 mmol; 8.2 mg), $Cu(MeCN)_4BF_4$ (0.01 equiv.; 0.01 mmol; 3.2 mg), **L2** (0.01 equiv.; 0.01 mmol; 2.1 mg), HFIP (0.02 equiv.; 0.02 mmol; 2 μL), and $NaNO_2$ (6.0 equiv.; 1.2 mmol; 84 mg). The reaction was worked up according to general workup procedure **(iv)** and purified by flash column chromatography (SiO_2; DCM, then 4:1:0.1 hexanes:EtOAc:AcOH) to afford compound **8f** (32.5 mg; 83%) as a yellow solid. **R_f** (4:1:0.1 hexanes:EtOAc:AcOH)=0.5. **IR** (film) ν_{max}/cm^{-1}: 3308, 2926, 2855, 1705, 1412, 1288, 1233, 936, 712, 628. **^{1}H NMR** (500 MHz, $CDCl_3$) δ 2.37 (t, *J* = 7.5 Hz, 2H), 2.20 (td, *J* = 7.1, 2.7 Hz, 2H), 1.96 (t, *J* = 2.6 Hz, 1H), 1.64 (q, *J* = 7.3 Hz, 2H), 1.54 (p, *J* = 7.1 Hz, 2H), 1.45 – 1.33 (m, 2H), 1.35 – 1.26 (m, 8H). **^{13}C NMR** (126 MHz, $CDCl_3$) δ 180.31, 84.89, 68.22, 34.18, 29.39, 29.29, 29.15, 28.83, 28.59, 24.78, 18.52. **HRMS** (ESI-TOF) mass calculated for $[M-H]^-$ ($C_{12}H_{19}O_2^-$) expected *m/z* 195.1391; found *m/z* 195.1390.

7-(benzylamino)-7-oxoheptanoic acid (**8g**)

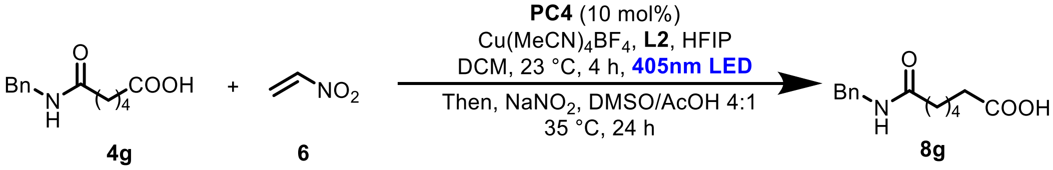

Prepared following general procedure **A**, using 6-(benzylamino)-6-oxohexanoic acid **4g** (1.0 equiv.; 0.2 mmol; 47.06 mg), nitroethylene **6** (1.1 equiv.; 0.21 mmol), **PC4** (0.10 equiv.; 0.02 mmol; 8.2 mg), $Cu(MeCN)_4BF_4$ (0.01 equiv.; 0.01 mmol; 3.2 mg), **L2** (0.01 equiv.; 0.01 mmol; 2.1 mg), HFIP (0.02 equiv.; 0.02 mmol; 2 µL), and $NaNO_2$ (6.0 equiv.; 1.2 mmol; 84 mg). The reaction was worked up according to general workup procedure **(i)** to afford compound **8g** (21.2 mg; 45%) as a white solid with no further purification required. **IR** (film) ν_{max}/cm^{-1}: 2978, 2932, 1705, 1671, 1522, 1456, 1392, 1367, 1251, 1154, 1052, 847; **M.P.** = 82 °C; **^{1}H NMR** ($CDCl_3$, 500 MHz) δ (ppm): 7.38 – 7.29 (m, 2H), 7.30 – 7.22 (m, 3H), 5.89 – 5.81 (t, J = 5.9 Hz, 1H), 4.46 – 4.40 (d, J = 5.6 Hz, 2H), 2.39 – 2.29 (t, J = 7.4 Hz, 2H), 2.28 – 2.18 (t, J = 7.5 Hz, 2H), 1.75 – 1.58 (m, 4H), 1.45 – 1.32 (m, 2H); **^{13}C NMR** ($CDCl_3$, 126 MHz) δ (ppm): 178.6, 173.1, 138.4, 128.9, 128.0, 127.7, 43.8, 36.5, 33.9, 28.7, 25.4, 24.5; **HRMS** (ESI-TOF) mass calculated for $[M-H]^-$ ($C_{14}H_{18}NO_3^-$) expected *m/z* 248.1292; found *m/z* 248.1295.

7-(phenylsulfonyl)heptanoic acid (**8h**)

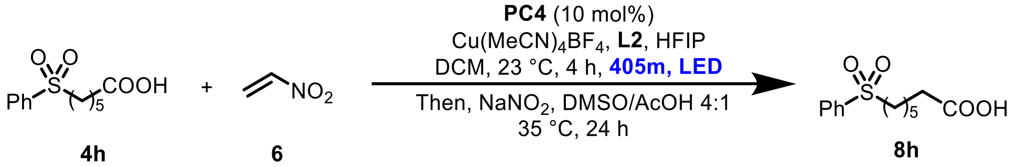

Prepared following general procedure **A**, using 6-(phenylsulfonyl)hexanoic acid **4h** (1.0 equiv.; 0.2 mmol; 51.3 mg), nitroethylene **2b** (1.1 equiv.; 0.21 mmol), **PC4** (0.10 equiv.; 0.02 mmol; 8.2 mg), $Cu(MeCN)_4BF_4$ (0.01 equiv.; 0.01 mmol; 3.2 mg), **L2** (0.01 equiv.; 0.01 mmol; 2.1 mg), HFIP (0.02 equiv.; 0.02 mmol; 2 µL), and $NaNO_2$ (6.0 equiv.; 1.2 mmol; 84 mg). The reaction was worked up according to general workup procedure **(ii)** to afford compound **8h** (43.8 mg; 81%) as a yellow solid with no further purification required. **IR** (film) ν_{max}/cm^{-1}: 2938, 2865, 1703, 1447, 1405, 1291, 1229, 1145, 1085, 795, 752, 688, 634; **^{1}H NMR** ($CDCl_3$, 500 MHz) δ (ppm): 7.96 – 7.89 (m, 2H), 7.72 – 7.65 (m, 1H), 7.64 – 7.56 (dd, J = 8.4, 7.1 Hz, 2H), 3.16 – 3.07 (m, 2H), 2.37 – 2.31 (t, J = 7.4 Hz, 2H), 1.80 – 1.68 (m, 2H), 1.67 – 1.57 (m, 2H), 1.48 – 1.32 (m, 4H); **^{13}C NMR** ($CDCl_3$, 126 MHz) δ (ppm): 179.1, 139.3, 133.8, 129.4,

128.2, 56.3, 33.8, 28.5, 28.0, 24.3, 22.6; **HRMS** (ESI-TOF) mass calculated for $[M\text{-}H]^-$ ($C_{13}H_{17}O_4S^-$) expected *m/z* 269.0853; found *m/z* 269.0852.

7-(diethoxyphosphoryl)heptanoic acid (**8i**)

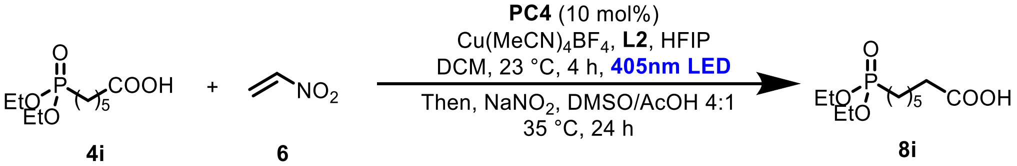

Prepared following general procedure **A**, using 6-(diethoxyphosphoryl)hexanoic acid **4i** (1.0 equiv.; 0.2 mmol; 50.5 mg), nitroethylene **6** (1.1 equiv.; 0.21 mmol), **PC4** (0.10 equiv.; 0.02 mmol; 8.2 mg), $Cu(MeCN)_4BF_4$ (0.01 equiv.; 0.01 mmol; 3.2 mg), **L2** (0.01 equiv.; 0.01 mmol; 2.1 mg), HFIP (0.02 equiv.; 0.02 mmol; 2 µL), and $NaNO_2$ (6.0 equiv.; 1.2 mmol; 84 mg). The reaction was worked up according to general workup procedure **(i)** to afford compound **8i** (39.0 mg; 73%) as a colourless oil with no further purification required. **IR** (film) ν_{max}/cm^{-1}:2982, 2934, 2866, 1724, 1394, 1193, 1098, 1053, 1026, 966, 822; **^{1}H NMR** ($CDCl_3$, 500 MHz) δ (ppm): 4.16 – 3.99 (m, 4H), 2.35 – 2.28 (t, J = 7.4 Hz, 2H), 1.79 – 1.67 (m, 2H), 1.66 – 1.52 (m, 4H), 1.45 – 1.32 (m, 2H), 1.32 – 1.27 (t, J = 7.1 Hz, 6H); **^{13}C NMR** ($CDCl_3$, 126 MHz) δ (ppm): 178.0, 61.8 (d, J = 6.6 Hz), 34.1, 30.3 (d, J = 16.9 Hz), 28.6, 25.6 (d, J = 140.7 Hz), 24.6, 22.3 (d, J = 5.3 Hz), 16.6 (d, J = 6.0 Hz); **HRMS** (ESI-TOF) mass calculated for $[M\text{-}H]^-$ ($C_{11}H_{22}O_5P^-$) expected *m/z* 265.1210; found *m/z* 265.1211.

2-cyclohexylacetic acid (**8j**)

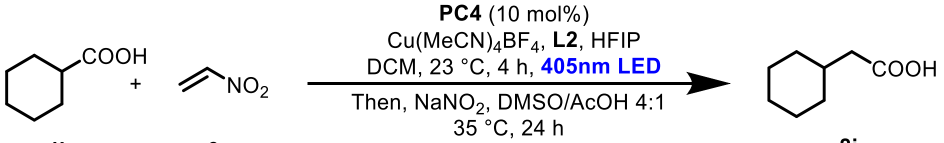

Prepared following general procedure A, using cyclohexanecarboxylic acid **4j** (1.0 equiv.; 0.2 mmol; 25.6 mg), nitroethylene **6** (1.1 equiv.; 0.21 mmol), **PC4** (0.10 equiv.; 0.02 mmol; 8.2 mg), $Cu(MeCN)_4BF_4$ (0.01 equiv.; 0.01 mmol; 3.2 mg), **L2** (0.01 equiv.; 0.01 mmol; 2.1 mg), HFIP (0.02 equiv.; 0.02 mmol; 2 µL), and $NaNO_2$ (6.0 equiv.; 1.2 mmol; 84 mg). The reaction was worked up according to general workup procedure **(i)** to afford compound **8j** (25.9 mg; 91%) as a white solid with no further purification required. **IR** (film) ν_{max}/cm^{-1}: 2923, 2852, 1701, 1449, 1410, 1296, 1236, 1193m 1121, 941; **^{1}H NMR** ($CDCl_3$, 500 MHz) δ (ppm): 2.25 – 2.20 (d, J = 6.8 Hz, 2H), 1.84 – 1.61 (m, 6H), 1.34 – 1.22 (m, 2H), 1.21 – 1.09 (m, 1H), 1.05 – 0.92 (m, 2H); **^{13}C NMR** ($CDCl_3$, 126 MHz) δ (ppm): 178.5, 41.8, 34.8, 33.1, 26.2, 26.1;

HRMS (ESI-TOF) mass calculated for $[2M-2H+Na]^-$ ($C_{16}H_{26}O_4Na^-$) expected *m/z* 305.1734; found *m/z* 305.1724.

2-(tetrahydro-2H-pyran-4-yl)acetic acid (8a)

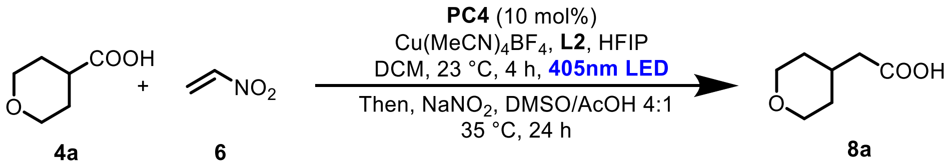

Prepared following general procedure **A**, using tetrahydro-2H-pyran-4-carboxylic acid **4a** (1.0 equiv.; 0.2 mmol; 26.0 mg), nitroethylene **6** (1.1 equiv.; 0.21 mmol), **PC4** (0.10 equiv.; 0.02 mmol; 8.2 mg), $Cu(MeCN)_4BF_4$ (0.01 equiv.; 0.01 mmol; 3.2 mg), **L2** (0.01 equiv.; 0.01 mmol; 2.1 mg), HFIP (0.02 equiv.; 0.02 mmol; 2 µL), and $NaNO_2$ (6.0 equiv.; 1.2 mmol; 84 mg). The reaction was worked up according to general workup procedure **(i)** to afford compound **8a** (25.8 mg; 91%) as a white solid with no further purification required. **IR** (film) ν_{max}/cm^{-1}: 2933, 2850, 1708, 1445, 1389, 1276, 1246, 1184, 1134, 1087, 884; **M.P.** = 55 °C; **^{1}H NMR** ($CDCl_3$, 500 MHz) δ (ppm): 4.01 – 3.93 (m, 2H), 3.47 – 3.37 (td, *J* = 11.9, 2.1 Hz, 2H), 2.32 – 2.27 (d, *J* = 7.1 Hz, 2H), 2.09 – 1.96 (ttt, *J* = 11.1, 7.3, 3.8 Hz, 1H), 1.72 – 1.64 (ddt, *J* = 13.2, 4.2, 2.2 Hz, 2H), 1.43 – 1.30 (dtd, *J* = 13.4, 11.8, 4.5 Hz, 2H); **^{13}C NMR** ($CDCl_3$, 126 MHz) δ (ppm): 178.2, 67.8, 41.2, 32.7, 32.0; **HRMS** (ESI-TOF) mass calculated for $[2M-2H+Na]^-$ ($C_{14}H_{22}O_6Na^-$) expected *m/z* 309.1320; found *m/z* 309.1313.

2-(1-(tert-butoxycarbonyl)piperidin-4-yl)acetic acid (8k)

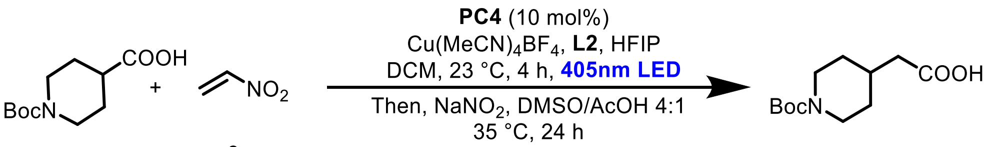

Prepared following general procedure **A**, using 1-(tert-butoxycarbonyl)piperidine-4-carboxylic acid **4k** (1.0 equiv.; 0.2 mmol; 45.86 mg), nitroethylene **6** (1.1 equiv.; 0.21 mmol), **PC4** (0.10 equiv.; 0.02 mmol; 8.2 mg), $Cu(MeCN)_4BF_4$ (0.01 equiv.; 0.01 mmol; 3.2 mg), **L2** (0.01 equiv.; 0.01 mmol; 2.1 mg), HFIP (0.02 equiv.; 0.02 mmol; 2 µL), and $NaNO_2$ (6.0 equiv.; 1.2 mmol; 84 mg). The reaction was worked up according to general workup procedure **(i)** to afford compound **8k** (41.8 mg; 86%) as a white solid with no further purification required. **IR** (film) ν_{max}/cm^{-1}: 2975, 2927, 1736, 1693, 1667, 1428, 1367, 1282, 1246, 1161, 1123, 974, 863, 770; **M.P.** = 97 °C; **^{1}H NMR** ($CDCl_3$, 500 MHz) δ (ppm): 4.16 – 4.02 (m, 2H), 2.76 – 2.66 (t, J = 12.8 Hz, 2H), 2.31 – 2.26 (d, J = 7.0 Hz, 2H), 2.00 – 1.85 (ttt, J = 10.9, 7.2, 3.7 Hz, 1H), 1.76

– 1.69 (m, 2H), 1.44 (s, 9H), 1.23 – 1.11 (qd, J = 12.3, 4.4 Hz, 2H); **^{13}C NMR** ($CDCl_3$, 126 MHz) δ (ppm): 178.1, 155.0, 79.7, 43.8, 40.9, 33.0, 31.9, 28.6; **HRMS** (ESI-TOF) mass calculated for $[M-H]^-$ ($C_{12}H_{20}NO_4^-$) expected *m/z* 242.1398; found *m/z* 242.1398.

2-(tetrahydrofuran-3-yl)acetic acid (**8l**)

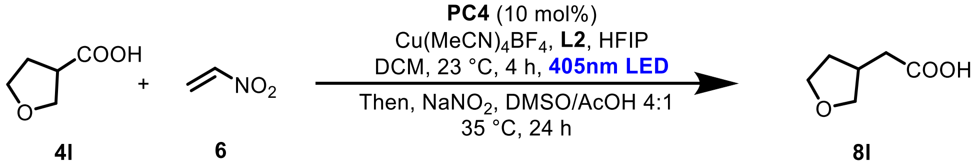

Prepared following general procedure **A**, using tetrahydrofuran-3-carboxylic acid **4l** (1.0 equiv.; 0.2 mmol; 23.2 mg; 19µl), nitroethylene **6** (1.1 equiv.; 0.21 mmol), **PC4** (0.1 equiv.; 0.02 mmol; 8.2 mg), $Cu(MeCN)_4BF_4$ (0.05 equiv.; 0.01 mmol; 3.2 mg), **L2** (0.05 equiv.; 0.01 mmol; 2.1 mg), HFIP (0.1 equiv.; 0.02 mmol; 2 µL), and $NaNO_2$ (6.0 equiv.; 1.2 mmol; 84 mg). The crude residue was subjected to general work-up procedure **(i)** and was purified by column chromatography (SiO_2; 4:1 hexanes:EtOAc) to afford compound **8l** (23.7 mg; 92%) as a colourless oil. **R_f** (4:1 hexanes:EtOAc)=0.2; **IR** (film) ν_{max}/cm^{-1}: 2921, 2150, 2026, 2008, 1713, 1412, 1275, 1210, 1175, 1039, 978, 895, 629, 479, 448; **^{1}H NMR** (500 MHz, CDCl3): δ 3.99 (dd, J = 8.7, 7.0 Hz, 1H), 3.91 (td, J = 8.3, 5.1 Hz, 1H), 3.80 (dt, J = 8.5, 7.4 Hz, 1H), 3.47 (dd, J = 8.6, 6.4 Hz, 1H), 2.71 – 2.60 (m, 1H), 2.52 – 2.45 (m, 2H), 2.18 (dtd, J = 12.7, 7.7, 5.2 Hz, 1H), 1.62 (ddt, J = 12.4, 8.0, 7.0 Hz, 1H).; **^{13}C NMR** (126 MHz, $CDCl_3$): δ 177.67, 72.96, 67.80, 37.63, 35.35, 32.14. **HRMS** (ESI-TOF) mass calculated for $[2M-2H+Na]^-$ ($C_{12}H_{18}O_6Na^-$) expected *m/z* 281.1007; found *m/z* 281.1005.

2-(tetrahydrofuran-2-yl)acetic acid (**8m**)

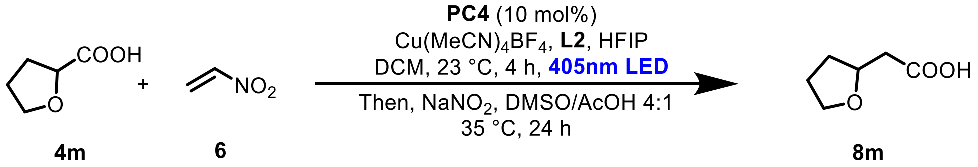

Prepared following general procedure **A**, using tetrahydrofuran-2-carboxylic acid **4m** (1.0 equiv.; 0.2 mmol; 23.2 mg; 19.2µl), nitroethylene **6** (1.1 equiv.; 0.21 mmol), **PC4** (0.1 equiv.; 0.02 mmol; 8.2 mg), $Cu(MeCN)_4BF_4$ (0.05 equiv.; 0.01 mmol; 3.2 mg), **L2** (0.05 equiv.; 0.01 mmol; 2.1 mg), HFIP (0.1 equiv.; 0.02 mmol; 2 µL), and $NaNO_2$ (6.0 equiv.; 1.2 mmol; 84 mg). The crude residue was subjected to general work-up procedure **(i)** and purified by flash column chromatography (SiO_2; 4:1 hexanes:EtOAc) to afford compound **8m** (15.9 mg; 61%)

as pale yellow oil. **IR** (film) ν_{max}/cm^{-1} : 2957, 2878, 1708, 1403, 1280, 1197, 1167, 1051, 1017, 922, 877, 827, 644, 489. **^{1}H NMR** (500 MHz, $CDCl_3$) δ 4.28 (qd, *J* = 7.3, 5.7 Hz, 1H), 3.98 – 3.88 (m, 1H), 3.86 – 3.78 (m, 1H), 2.69 – 2.54 (m, 2H), 2.14 (dddd, *J* = 12.1, 8.2, 6.6, 5.2 Hz, 1H), 2.04 – 1.88 (m, 2H), 1.60 (ddt, *J* = 12.3, 8.7, 7.5 Hz, 1H). **^{13}C NMR** (126 MHz, $CDCl_3$) δ 176.09, 75.16, 68.28, 40.36, 31.38, 25.67. **HRMS** (ESI-TOF) mass calculated for [2M-2H+Na]$^-$ ($C_{12}H_{18}O_6Na^-$) expected *m/z* 281.1007; found *m/z* 281.0998.

2-(2,3-dihydrobenzofuran-2-yl)acetic acid (8n)

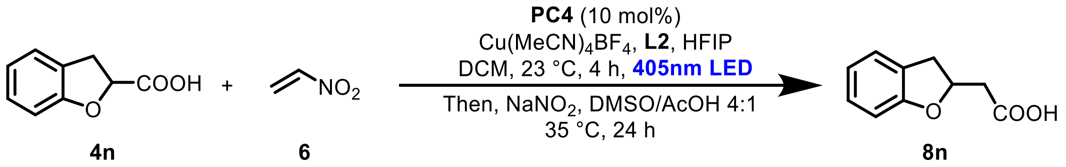

Prepared following general procedure **A**, using 2,3-dihydrobenzofuran-2-carboxylic acid **4n** (1.0 equiv.; 0.2 mmol; 23.2 mg; 19.2µl), nitroethylene **6** (1.1 equiv.; 0.21 mmol), **PC4** (0.1 equiv.; 0.02 mmol; 8.2 mg), $Cu(MeCN)_4BF_4$ (0.05 equiv.; 0.01 mmol; 3.2 mg), **L2** (0.05 equiv.; 0.01 mmol; 2.1 mg), HFIP (0.1 equiv.; 0.02 mmol; 2 µL), and $NaNO_2$ (6.0 equiv.; 1.2 mmol; 84 mg). The crude residue was subjected to general work-up procedure **(i)** and purified by flash column chromatography (SiO_2; 4:1 hexanes:EtOAc) to afford compound **8n** (23.5 mg; 66%) as a pale yellow oil; **IR** (film) ν_{max}/cm^{-1}: 3049, 2923, 1708, 1597, 1483, 1463, 1436, 1290, 1239, 1216, 1175, 1149, 1100, 1006, 928, 877, 831, 792, 746, 712, 664, 508, 444, 419; **^{1}H NMR** (500 MHz, $CDCl_3$) δ 7.20 (dt, J = 7.2, 1.3 Hz, 1H), 7.15 (td, J = 7.8, 1.3 Hz, 1H), 6.89 (td, J = 7.4, 1.0 Hz, 1H), 6.82 (d, J = 8.0 Hz, 1H), 5.21 (dtd, J = 9.1, 7.2, 6.0 Hz, 1H), 3.47 (dd, J = 15.6, 9.1 Hz, 1H), 2.97 (ddd, J = 16.2, 12.8, 7.3 Hz, 2H), 2.77 (dd, J = 16.2, 6.0 Hz, 1H).; **^{13}C NMR** (126 MHz, $CDCl_3$) δ 175.92, 159.00, 128.38, 126.01, 125.15, 120.91, 109.84, 78.45, 40.57, 35.49. **HRMS** (ESI-TOF) mass calculated for [2M-2H+Na]$^-$ ($C_{20}H_{18}O_6Na^-$) expected *m/z* 377.1007; found *m/z* 377.0994.

2-(2,3-dihydrobenzofuran-2-yl)acetic acid (8o)

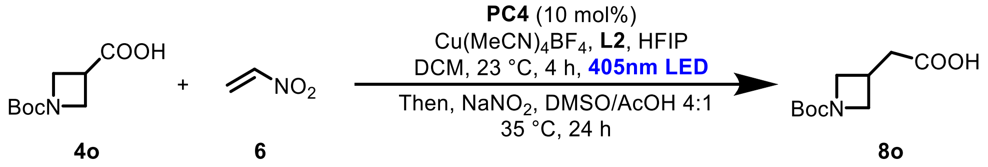

Prepared following general procedure **A**, using 1-(tert-butoxycarbonyl)azetidine-3-carboxylic acid **4o** (1.0 equiv.; 0.2 mmol; 40.24 mg), nitroethylene **6** (1.1 equiv.; 0.21 mmol), **PC4** (0.15 equiv.; 0.03 mmol; 12.3 mg), $Cu(MeCN)_4BF_4$ (0.05 equiv.; 0.01 mmol; 3.2 mg), **L2** (0.05

equiv.; 0.01 mmol; 2.1 mg), HFIP (0.1 equiv.; 0.02 mmol; 2 µL), and $NaNO_2$ (6.0 equiv.; 1.2 mmol; 84 mg). The crude residue was subjected to general work-up procedure **(i)** and purified by flash column chromatography (SiO_2; 4:1:0.1 hexanes:EtOAc:AcOH) to afford compound **8o** (27 mg; 63%) as a white solid. $\mathbf{R_f}$ (4:1:0.1 hexanes:EtOAc:AcOH)=0.35; **IR** (film) ν_{max}/cm^{-1}: 2975, 2887, 1700, 1674, 1479,1417, 1367, 1296, 1250, 1146, 1009, 933, 856, 771, 656, 564, 464, 421; **M.P.** = 107 °C; ^{1}**H NMR** (500 MHz, $CDCl_3$) δ 4.15 – 4.09 (m, 2H), 3.65 (dd, J = 8.9, 5.4 Hz, 2H), 2.90 (pt, J = 8.1, 5.4 Hz, 1H), 2.70 (d, J = 7.8 Hz, 2H), 1.45 (s, 9H); ^{13}**C NMR** (126 MHz, $CDCl_3$) δ 176.53, 156.51, 79.81, 54.33, 38.24, 28.53, 25.03. **HRMS** (ESI-TOF) mass calculated for $[M-H]^-$ ($C_{10}H_{16}NO_4^-$) expected *m/z* 214.1085; found *m/z* 214.1084.

1-Adamantaneacetic acid (8p)

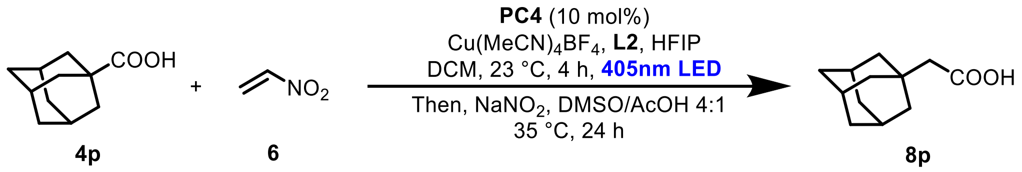

Prepared following general procedure **A**, using adamantane carboxylic acid **4p** (1.0 equiv.; 0.2 mmol; 36 mg), nitroethylene **6** (1.1 equiv.; 0.21 mmol), **PC4** (0.1 equiv.; 0.02 mmol; 8.2 mg), $Cu(MeCN)_4BF_4$ (0.05 equiv.; 0.01 mmol; 3.2 mg), **L2** (0.05 equiv.; 0.01 mmol; 2.1 mg), HFIP (0.1 equiv.; 0.02 mmol; 2 µL), and $NaNO_2$ (6.0 equiv.; 1.2 mmol; 84 mg). The crude residue was subjected to general work-up procedure **(iv)** and purified by flash column chromatography (SiO_2; 4:1:0.1 hexanes:EtOAc:AcOH) to afford compound **8p** (31.4 mg; 81%) as a white solid. $\mathbf{R_f}$ (4:1:0.1 hexanes:EtOAc:AcOH)=0.6. **IR** (film) ν_{max}/cm^{-1}: 2901, 2848, 2657, 1692, 1450, 1413, 1364, 1339, 1319, 1292, 1273, 1232, 1207, 1158, 1101, 965, 705, 650, 633, 466; ^{1}**H NMR** (500 MHz, CDC13) δ 2.13 (s, 2H), 2.01 (p, J = 3.1 Hz, 3H), 1.73 (dt, J = 12.3, 3.0 Hz, 3H), 1.68 (d, J = 3.0 Hz, 9H).; ^{13}**C NMR** (126 MHz, $CDCl_3$) δ 177.68, 48.74, 42.43, 36.84, 32.83, 28.75. **HRMS** (ESI-TOF) mass calculated for $[M-H]^-$ ($C_{12}H_{17}O_2^-$) expected *m/z* 193.1234; found *m/z* 193.1234.

2-(1-phenylcyclopropyl)acetic acid (8q)

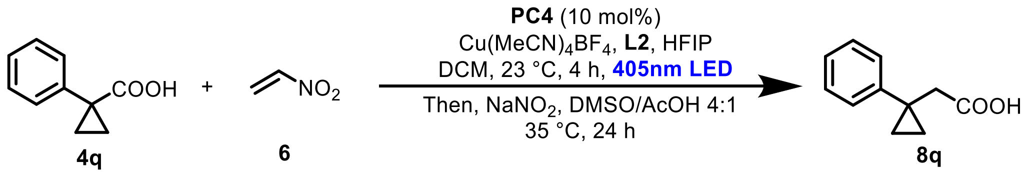

Prepared following general procedure **A**, using 1-phenylcyclopropane-1-carboxylic acid **4q** (1.0 equiv.; 0.2 mmol; 32.4 mg), nitroethylene **6** (1.1 equiv.; 0.21 mmol), **PC4** (0.15 equiv.;

0.03 mmol; 12.3 mg), $Cu(MeCN)_4BF_4$ (0.05 equiv.; 0.01 mmol; 3.2 mg), **L2** (0.05 equiv.; 0.01 mmol; 2.1 mg), HFIP (0.1 equiv.; 0.02 mmol; 2 µL), and $NaNO_2$ (6.0 equiv.; 1.2 mmol; 84 mg). The crude residue was subjected to general work-up procedure **(i)** and purified by flash column chromatography (SiO_2; 4:1:0.1 hexanes:EtOAc:AcOH) to afford compound **8q** (27.8 mg; 79%) as a white solid. $\mathbf{R_f}$ (4:1:0.1 hexanes:EtOAc:AcOH)=0.42. **IR** (film) ν_{max}/cm^{-1}: 3025, 1706, 1603, 1498, 1445, 1413, 1290, 1222, 1104, 1077, 1026, 995, 935, 849, 759, 699, 548. ^{1}**H NMR** (500 MHz, $CDCl_3$) δ 7.33 – 7.3 (m, 2H), 7.29 – 7.25 (m, 2H), 7.21 – 7.16 (m, 1H), 2.61 (s, 2H), 1. – 0.88 (m, 4H). ^{13}**C NMR** (126 MHz, $CDCl_3$) δ 177.67, 144.00, 128.41, 128.37, 126.48, 44.46, 22.01, 13.94. **HRMS** (ESI-TOF) mass calculated for [2M-2H+Na]$^-$ ($C_{22}H_{22}O_4^-$) expected *m/z* 373.1421; found *m/z* 373.1413.

2-(3-(methoxycarbonyl)bicyclo[1.1.1]pentan-1-yl)acetic acid (8r)

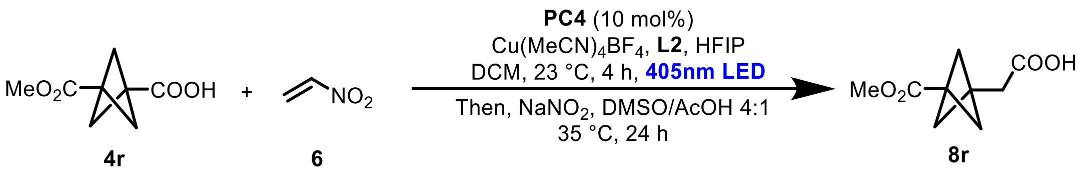

Prepared following general procedure **A**, using 3-(methoxycarbonyl)bicyclo[1.1.1]pentane-1-carboxylic acid **4r** (1.0 equiv.; 0.2 mmol; 34.03 mg), nitroethylene **6** (1.1 equiv.; 0.21 mmol), **PC4** (0.10 equiv.; 0.02 mmol; 8.2 mg), $Cu(MeCN)_4BF_4$ (0.01 equiv.; 0.01 mmol; 3.2 mg), **L2** (0.01 equiv.; 0.01 mmol; 2.1 mg), HFIP (0.02 equiv.; 0.02 mmol; 2 µL), and $NaNO_2$ (6.0 equiv.; 1.2 mmol; 84 mg). The reaction was worked up according to general workup procedure **(iii)** to afford compound **8r** (17.3 mg; 47%) as a white solid with no further purification required. **IR** (film) ν_{max}/cm^{-1}: 2954, 2919, 2871, 1722, 1700, 1457, 1432, 1408, 1289, 1240, 1113, 1067, 1011, 957; **M.P.** = 113 °C; ^{1}**H NMR** ($CDCl_3$, 500 MHz) δ (ppm): 3.67 (s, 3H), 2.58 (s, 2H), 2.08 (s, 6H); ^{13}**C NMR** ($CDCl_3$, 126 MHz) δ (ppm): 176.8, 170.3, 52.6, 51.8, 38.4, 36.7, 35.8; **HRMS** (ESI-TOF) mass calculated for [M-H]$^-$ ($C_9H_{11}O_4^-$) expected *m/z* 183.0663; found *m/z* 183.0665.

2-(4-(methoxycarbonyl)bicyclo[2.2.2]octan-1-yl)acetic acid (8s)

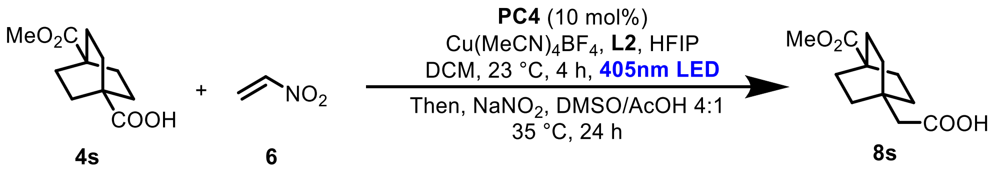

Prepared following general procedure **A**, using 4-(methoxycarbonyl)bicyclo[2.2.2]octane-1-carboxylic acid **4s** (1.0 equiv.; 0.2 mmol; 42.5 mg), nitroethylene **6** (1.1 equiv.; 0.21 mmol),

PC4 (0.10 equiv.; 0.02 mmol; 8.2 mg), $Cu(MeCN)_4BF_4$ (0.01 equiv.; 0.01 mmol; 3.2 mg), **L2** (0.01 equiv.; 0.01 mmol; 2.1 mg), HFIP (0.02 equiv.; 0.02 mmol; 2 µL), and $NaNO_2$ (6.0 equiv.; 1.2 mmol; 84 mg). The reaction was worked up according to general workup procedure **(i)** to afford compound **8s** (31.8 mg; 70%) as a white solid with no further purification required. **IR** (film) ν_{max}/cm^{-1}: 2966, 2918, 2872, 2859, 1722, 1701, 1458, 1432, 1409, 1290, 1240 1171, 1139, 1066; **M.P.** = 94 °C; **^{1}H NMR** ($CDCl_3$, 500 MHz) δ (ppm): 3.63 (s, 3H), 2.14 (s, 2H), 1.83 – 1.76 (m, 6H), 1.61 – 1.54 (m, 6H); **^{13}C NMR** ($CDCl_3$, 126 MHz) δ (ppm): 178.4, 178.1, 51.8, 45.5, 38.7, 30.8, 30.5, 28.5; **HRMS** (ESI-TOF) mass calculated for $[M-H]^-$ ($C_{12}H_{17}O_4^-$) expected *m/z* 225.1132; found *m/z* 225.1134.

2-((1S,4R)-7,7-dimethyl-2-oxobicyclo[2.2.1]heptan-1-yl)acetic acid (8t)

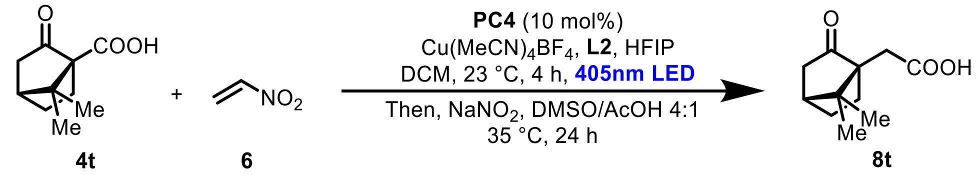

Prepared following general procedure **A**, using (1S)-(+)-Ketopinic acid **4t** (1.0 equiv.; 0.2 mmol; 36.4 mg), nitroethylene **6** (1.1 equiv.; 0.21 mmol), **PC4** (0.15 equiv.; 0.03 mmol; 12.3 mg), $Cu(MeCN)_4BF_4$ (0.05 equiv.; 0.01 mmol; 3.2 mg), **L2** (0.05 equiv.; 0.01 mmol; 2.1 mg), HFIP (0.1 equiv.; 0.02 mmol; 2 µL), and $NaNO_2$ (6.0 equiv.; 1.2 mmol; 84 mg). The crude residue was subjected to general work-up procedure **(i)** and purified by column chromatography (SiO_2; 4:1:0.1 hexanes:EtOAc:AcOH) to afford compound **8t** (14.5 mg; 37%) as a white solid. **R_f** (4:1:0.1 hexanes:EtOAc:AcOH)=0.38. **$[\alpha]_D^{25}$** = –16 (c = 1.00 CHCl3); **IR** (film) ν_{max}/cm^{-1}: 2959, 1739, 1707, 1454, 1415, 1392, 1375, 1314, 1293, 1198, 1173, 1132, 1104, 1070, 1052, 1033, 936, 853, 787, 670, 619, 591, 537, 449. **^{1}H NMR** (500 MHz, $CDCl_3$) δ 2.61 (d, *J* = 14.6 Hz, 1H), 2.49 (ddd, *J* = 18.8, 4.9, 2.4 Hz, 1H), 2.29 (d, *J* = 14.6 Hz, 1H), 2.16 (t, *J* = 4.2 Hz, 1H), 2.11 – 1.95 (m, 3H), 1.76 – 1.66 (m, 1H), 1.50 – 1.40 (m, 1H), 1.02 (s, 3H), 0.93 (s, 3H); **^{13}C NMR** (126 MHz, $CDCl_3$) δ 221.31, 174.3, 59.25, 48.18, 43.35, 43.07, 32.19, 26.87, 26.63, 19.96, 19.37. **HRMS** (ESI-TOF) mass calculated for $[M-H]^-$ ($C_{11}H_{15}O_3^-$) expected *m/z* 195.1027; found *m/z* 195.1024.

6-(2,5-dimethylphenoxy)-3,3-dimethylhexanoic acid (8u)

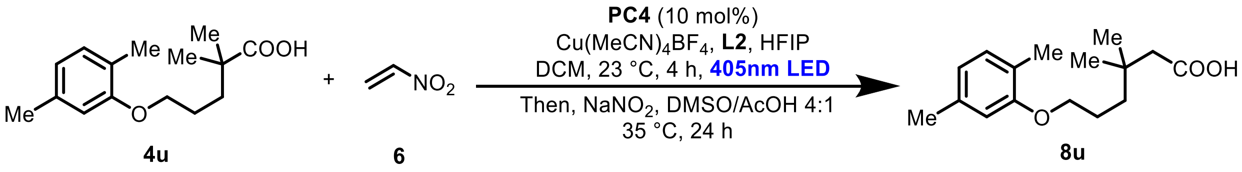

Prepared following general procedure **A**, using Gemfibrozil **4u** (1.0 equiv.; 0.2 mmol; 50.1 mg), nitroethylene **6** (1.1 equiv.; 0.21 mmol), **PC4** (0.10 equiv.; 0.02 mmol; 8.2 mg), $Cu(MeCN)_4BF_4$ (0.01 equiv.; 0.01 mmol; 3.2 mg), **L2** (0.01 equiv.; 0.01 mmol; 2.1 mg), HFIP (0.02 equiv.; 0.02 mmol; 2 µL), and $NaNO_2$ (6.0 equiv.; 1.2 mmol; 84 mg). The reaction was worked up according to general workup procedure **(iv)** and the crude residue was purified by flash column chromatography (SiO_2; gradient 9:1:0.1 to 7:3:0.1 hexanes:EtOAc:AcOH; eluent removed using a rotary evaporator, maintaining the water bath at 35 °C and vacuum pressure 130 – 80 mbar) to afford compound **8u** (27.1 mg; 51%) as a pale yellow oil. **IR** (film) ν_{max}/cm^{-1}:2957, 2161, 2051, 2032, 1704, 1615, 1585, 1509, 1413, 1390, 1265, 1158, 1130, 804; **^{1}H NMR** ($CDCl_3$, 500 MHz) δ (ppm): 7.04 – 6.98 (d, J = 7.4 Hz, 1H), 6.69 – 6.64 (d, J = 7.5 Hz, 1H), 6.63 (s, 1H), 3.99 – 3.89 (t, J = 6.4 Hz, 2H), 2.32 (s, 3H), 2.30 (s, 2H), 2.18 (s, 3H), 1.86 – 1.75 (m, 2H), 1.59 – 1.50 (m, 2H), 1.09 (s, 6H); **^{13}C NMR** ($CDCl_3$, 126 MHz) δ (ppm): 178.7, 157.1, 136.6, 130.4, 123.7, 120.8, 112.1, 68.4, 45.9, 38.4, 33.2, 27.4, 24.5, 21.5, 15.9; **HRMS** (ESI-TOF) mass calculated for $[M-H]^-$ ($C_{16}H_{23}O_3^-$) expected *m/z* 263.1653; found *m/z* 263.1648.

(R)-5-((3R,5R,8R,9S,10S,13R,14S,17R)-3-hydroxy-10,13-dimethylhexadecahydro-1H-cyclopenta[a]phenanthren-17-yl)hexanoic acid (8v)

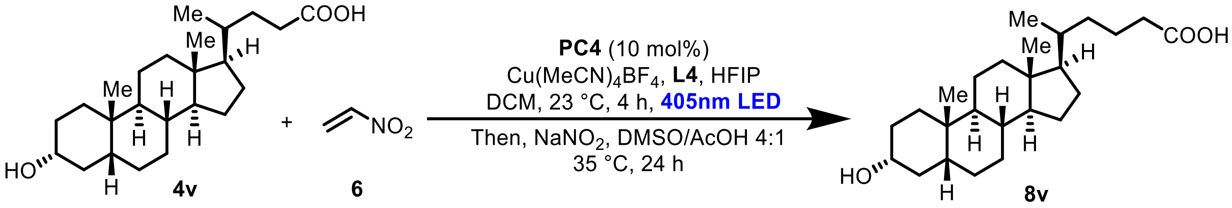

Prepared following general procedure **A**, using Lithocholic acid **4v** (1.0 equiv.; 0.2 mmol; 75.3 mg), nitroethylene **6** (1.1 equiv.; 0.21 mmol), **PC4** (0.10 equiv.; 0.02 mmol; 8.2 mg), $Cu(MeCN)_4BF_4$ (0.01 equiv.; 0.01 mmol; 3.2 mg), **L2** (0.01 equiv.; 0.01 mmol; 2.1 mg), HFIP (0.02 equiv.; 0.02 mmol; 2 µL), and $NaNO_2$ (6.0 equiv.; 1.2 mmol; 84 mg). The crude residue was subjected to general work-up procedure **(iv)** and purified by flash column chromatography

(SiO_2; 4:1:0.1 hexanes:EtOAc:AcOH) to afford compound **8v** (49 mg; 63%) as a pale yellow solid. **R_f** (4:1:0.1 hexanes:EtOAc:AcOH)=0.42. **$[\alpha]_D^{25}$** = +76 (c = 1.00 CHCl3); **IR** (film) ν_{max}/cm^{-1}: 3351, 2929, 2864, 1708, 1449, 1376, 1262, 1089, 1067, 1033, 1013, 945, 738, 606, 476; **M.P.** = 118 °C; **^{1}H NMR** ($CDCl_3$, 101 MHz) δ (ppm): δ 6.23 – 5.21 (m, 1H), 3.65 (tt, *J* = 10.8, 4.7 Hz, 1H), 2.41 – 2.22 (m, 2H), 2.00 – 1.92 (m, 1H), 1.89 – 1.62 (m, 6H), 1.61 – 1.45 (m, 3H), 1.47 – 1.30 (m, 8H), 1.30 – 0.99 (m, 10H), 0.98 – 0.95 (m, 1H), 0.93 (s, 3H), 0.91 (s, 3H), 0.64 (s, 3H); **^{13}C NMR** ($CDCl_3$, 126 MHz) δ (ppm): 179.57, 77.4, 72.1, 56.6, 56.1, 42.8, 42.2, 40.6, 40.3, 36.4, 36.0, 35.7, 35.5, 34.7, 34.6, 30.55, 28.4, 27.4, 26.6, 24.4, 23.5, 21.6, 21.0, 18.7, 12.2; **HRMS** (ESI-TOF) mass calculated for $[M-H]^-$ ($C_{25}H_{41}O_3^-$) expected *m/z* 389.3061; found *m/z* 389.3050.

2-((4aR,6aS,6bR,8aR,10S,12aR,12bR,14bR)-10-hydroxy-2,2,6a,6b,9,9,12a-heptamethyl-1,3,4,5,6,6a,6b,7,8,8a,9,10,11,12,12a,12b,13,14b-octadecahydropicen-4a(2H)-yl)acetic acid (8w)

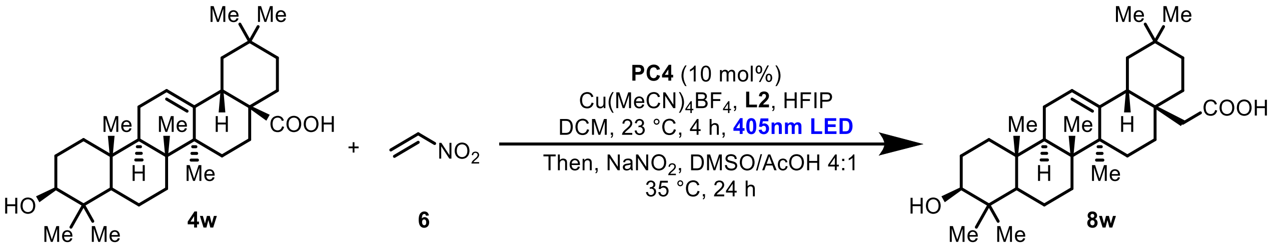

Prepared following general procedure **A**, using Oleanolic acid **8w** (1.0 equiv.; 0.2 mmol; 91.34 mg), nitroethylene **6** (1.1 equiv.; 0.21 mmol), **PC4** (0.10 equiv.; 0.02 mmol; 8.2 mg), $Cu(MeCN)_4BF_4$ (0.01 equiv.; 0.01 mmol; 3.2 mg), **L2** (0.01 equiv.; 0.01 mmol; 2.1 mg), HFIP (0.02 equiv.; 0.02 mmol; 2 µL), and $NaNO_2$ (6.0 equiv.; 1.2 mmol; 84 mg). The reaction was worked up according to general workup procedure **(iv)** and the crude residue was purified by flash column chromatography (SiO_2; gradient 9:1:0.1 to 7:3:0.1 hexanes:EtOAc:AcOH; eluent removed using a rotary evaporator, maintaining the water bath at 35 °C and vacuum pressure 130 – 80 mbar) to afford compound **8w** (33.9 mg; 36%, >20:1 dr) as a pale yellow oil that crystallizes on standing. The relative configuration of the chiral center involved in the reactivity is assigned according to previously reported Giese-type radical addition, assuming analogous substrate control.[9] **$[\alpha]_D^{25}$** = +58 (c = 1.00 CHCl3); **IR** (film) ν_{max}/cm^{-1}: 3440, 2970, 2944, 1738, 1447, 1366, 1229, 1217, 1093, 1031, 991; **^{1}H NMR** ($CDCl_3$, 500 MHz) δ (ppm): 5.26 – 5.19 (t, J = 3.6 Hz, 1H), 3.27 – 3.18 (dd, J = 11.3, 4.7 Hz, 1H), 2.61 – 2.54 (d, J = 13.7 Hz, 1H), 2.13 – 2.03 (dd, J = 13.7, 4.5 Hz, 1H), 2.01 – 1.95 (d, J = 13.7 Hz, 2H), 1.92 – 1.79 (m, 3H), 1.67 – 1.49 (dtd, J = 17.8, 10.9, 10.5, 4.6 Hz, 9H), 1.43 – 1.21 (m, 6H), 1.20 – 1.11 (m, 5H),

1.03 – 0.95 (m, 9H), 0.93 (s, 3H), 0.88 (s, 6H), 0.79 (s, 3H); **^{13}C NMR** ($CDCl_3$, 126 MHz) δ (ppm): 178.1, 144.0, 123.02 79.2, 55.3, 47.7, 46.8, 46.5, 44.1, 41.7, 40.0, 38.9, 38.7, 37.1, 36.2, 34.4, 33.3, 32.5, 31.1, 31.0, 28.2, 27.3, 26.4, 26.1, 23.8, 23.73, 23.65, 18.5, 16.8, 15.7, 15.6; **HRMS** (ESI-TOF) mass calculated for $[M-H]^-$ ($C_{31}H_{49}O_3^-$) expected *m/z* 469.3687; found *m/z* 469.3679.

(S)-6-(tert-butoxy)-5-((tert-butoxycarbonyl)amino)-6-oxohexanoic acid (8x)

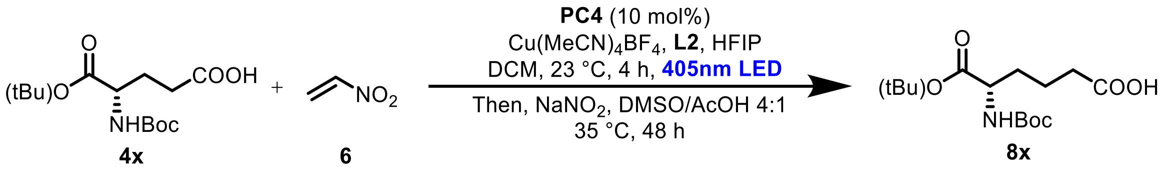

Prepared following general procedure **A,** using (S)-5-(tert-butoxy)-4-((tert-butoxycarbonyl)amino)-5-oxopentanoic acid **4x** (1.0 equiv.; 0.2 mmol; 60.7 mg), nitroethylene **6** (1.1 equiv.; 0.21 mmol), **PC4** (0.15 equiv.; 0.02 mmol; 12.3 mg), $Cu(MeCN)_4BF_4$ (0.01 equiv.; 0.01 mmol; 3.2 mg), **L2** (0.01 equiv.; 0.01 mmol; 2.1 mg), HFIP (0.02 equiv.; 0.02 mmol; 2 μL), and $NaNO_2$ (6.0 equiv.; 1.2 mmol; 84 mg). The reaction was worked up according to general workup procedure **(iii)** to afford compound **8x** (53.3 mg; 84%) as a white solid with no further purification required. **$[\alpha]_D^{25}$** = +14 (c = 1.00 CHCl3); **IR** (film) ν_{max}/cm^{-1}: 2978, 2934, 1713, 1507y, 1456, 1394, 1368, 1250, 1164, 1056, 846, 781; **M.P.** = 74 °C; **^{1}H NMR** ($CDCl_3$, 500 MHz) δ (ppm): 6.02 – 5.03 (m, 1H), 4.30 – 3.86 (m, 1H), 2.46 – 2.31 (m, 2H), 1.91 – 1.75 (m, 1H), 1.73 – 1.62 (m, 3H), 1.46 (s, 9H), 1.43 (s, 9H); **^{13}C NMR** ($CDCl_3$, 126 MHz) δ (ppm): 178.6, 171.9, 155.6, 82.2, 79.9, 53.7, 33.5, 32.3, 28.5, 28.1, 20.5; **HRMS** (ESI-TOF) mass calculated for $[M-H]^-$ ($C_{15}H_{26}NO_6^-$) expected *m/z* 316.1766; found *m/z* 316.1759.

(S)-7-(tert-butoxy)-6-((tert-butoxycarbonyl)amino)-7-oxoheptanoic acid (8y)

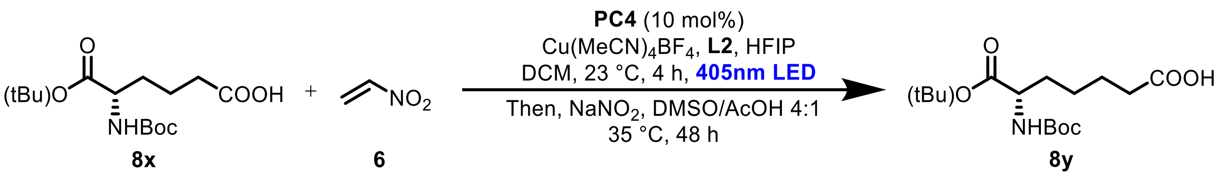

Prepared following general procedure **A,** using (S)-6-(tert-butoxy)-5-((tert-butoxycarbonyl)amino)-6-oxohexanoic acid **8x** (1.0 equiv.; 0.132 mmol; 41.9mg), nitroethylene **6** (1.1 equiv.; 0.21 mmol), **PC4** (0.10 equiv.; 0.02 mmol; 8.2 mg), $Cu(MeCN)_4BF_4$ (0.01 equiv.; 0.01 mmol; 3.2 mg), **L2** (0.01 equiv.; 0.01 mmol; 2.1 mg), HFIP (0.02 equiv.; 0.02 mmol; 2 μL), and $NaNO_2$ (6.0 equiv.; 1.2 mmol; 84 mg). The reaction was

worked up according to general workup procedure **(iii)** to afford compound **8y** (28.9 mg; 66%) as a white solid with no further purification required. **[α]$_D$^{25}** = +8 (c = 1.00 CHCl3); **IR** (film) ν_{max}/cm^{-1}: 3318, 2978, 2934, 1714, 1507 1457, 1394, 1368, 1252, 1155, 1063, 848; **^{1}H NMR** ($CDCl_3$, 500 MHz) δ (ppm): 5.62 – 4.99 (m, 1H), 4.24 – 3.90 (m, 1H), 2.40 – 2.30 (t, J = 7.4 Hz, 2H), 1.85 – 1.72 (m, 1H), 1.72 – 1.57 (m, 3H), 1.47 – 1.34 (m, 20H); **^{13}C NMR** ($CDCl_3$, 126 MHz) δ (ppm): 178.7, 172.1, 155.6, 82.1, 79.8, 53.9, 33.8, 32.8, 28.5, 28.1, 24.8, 24.5; **HRMS** (ESI-TOF) mass calculated for [M-H]$^-$ ($C_{16}H_{28}NO_6^-$) expected *m/z* 330.1922; found *m/z* 330.1917.

tert-butyl (S)-6-amino-2-((tert-butoxycarbonyl)amino)-6-oxohexanoate (9)

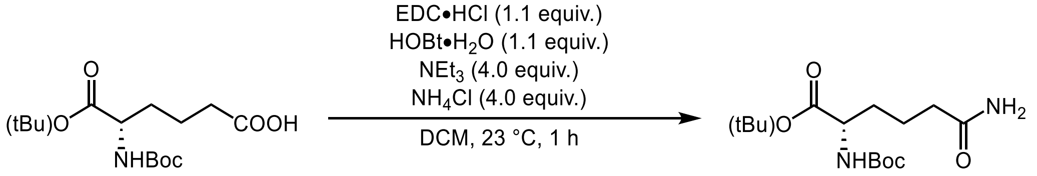

8x **9**

Amide **9** was prepared according to a modified literature procedure.[10]

Carboxylic acid **8x** (1.0 equiv.; 0.1 mmol; 30.4 mg) was weighed into a round bottom flasked charged with magnetic stirrer bar. EDC•HCl (1.1 equiv.; 0.11 mmol; 17.1 mg), HOBT•H_2O (1.1 equiv.; 0.11 mmol, 14.9 mg), and NH_4Cl (4.0 equiv.; 0.4 mmol; 21.4 mg) were weighed and added to the round bottom flask. The reagents were dissolved in DCM (0.5 mL) and NEt_3 (4.0 equiv.; 0.4 mmol; 55.8 µL) was added by microsyringe. The reaction was stirred at room temperature for 16 hours. Upon completion, the reaction was diluted with ethyl acetate and washed with HCl (0.5 M), water, saturated aqueous $NaHCO_3$, and brine. The organic layer was separated, dried (Na_2SO_4), filtered, and concentrated in vacuo. The crude residue was purified by flash column chromatography (SiO_2; 3:1 DCM:Acetone; eluent removed using a rotary evaporator, maintaining the water bath at 35 °C and vacuum pressure 350 – 250 mbar) to afford compound **9** (19.9 mg; 63%) as a white solid.

[α]$_D$^{25} = +20 (c = 1.00 CHCl3); **IR** (film) ν_{max}/cm^{-1}: 3347, 2978, 2932, 1705, 1671, 1522, 1456, 1392, 1367, 1251, 1154, 1052, 847; **M.P.** = 93 °C; **^{1}H NMR** ($CDCl_3$, 500 MHz) δ (ppm): 5.73 (br s, 1H), 5.59 (br s, 1H), 5.21 – 4.98 (d, J = 8.3 Hz, 1H), 4.24 – 3.86 (q, J = 3.0 Hz, 1H), 2.38 – 2.28 (m, 1H), 2.28 – 2.14 (m, 1H), 1.86 – 1.59 (m, 4H), 1.46 (s, 9H), 1.43 (s, 9H); **^{13}C NMR** ($CDCl_3$, 126 MHz) δ (ppm): 175.3, 171.9, 155.8, 82.2, 79.9, 53.3, 35.0, 32.6, 28.5, 28.1, 21.5; **HRMS** (ESI-TOF) mass calculated for [M+Na]$^+$ ($C_{15}H_{28}N_2O_5Na^+$) expected *m/z* 339.1890; found *m/z* 339.1885.

tert-butyl (S)-2-((tert-butoxycarbonyl)amino)-6-hydroxyhexanoate (**10**)

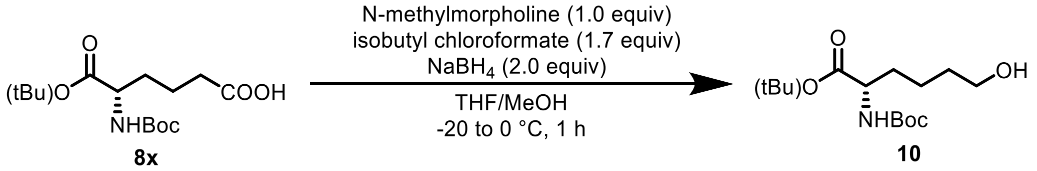

Alcohol **10** was prepared according to a modified literature procedure.[11] An oven-dried round bottom flask charged with carboxylic acid **8x** (1.0 equiv.; 0.088 mmol; 28.3 mg) and a magnetic stirrer bar was evacuated and refilled with argon three times. The acid was dissolved in THF (300 µL) and cooled to -20 °C. N-methyl morpholine (1.0 equiv.; 0.088 mmol; 10 µL), was added by microsyringe followed by slow addition of isobutyl chloroformate (1.7 equiv.; 0.149 mmol; 11.4 µL) by microsyringe. The reaction mixture was stirred at -20 °C for 10 minutes before warming to room temperature. The flask was opened to air and the solution was taken up in a syringe, leaving behind the precipitate, and transferred to a second oven-dried round bottom flask charged with magnetic stirrer bar, the precipitant was rinsed with THF (0.7 mL), and this was also transferred to the new round bottom flask. Methanol (0.1 mL) was added followed by $NaBH_4$ (2.0 equiv.; 0.177 mmol; 6.7 mg). The reaction was stirred at 0 °C for 1 hour. Upon completion the reaction was diluted with diethyl ether and HCl aq. (1M). The organic layer was separated, washed with saturated aq. $NaHCO_3$ and brine, and dried (Na_2SO_4), filtered, and concentrated *in vacuo*.

The crude residue was purified by flash column chromatography (SiO_2; gradient 8:2 to 1:1 EtOAc:DCM; eluent removed using a rotary evaporator, maintaining the water bath at 35 °C and vacuum pressure 250 – 80 mbar) to afford compound **10** (15.2 mg; 57%) as a colourless oil. **$[\alpha]_D^{25}$** = +8 (c = 1.00 CHCl3); **IR** (film) ν_{max}/cm^{-1}: 3475, 3460, 3441, 2978, 2932, 2872, 2612, 1715, 1508, 1457, 1392, 1367, 1251, 1155, 1058, 1024, 694; **^{1}H NMR** ($CDCl_3$, 500 MHz) δ (ppm) : 5.14 – 4.61 (d, J = 8.3 Hz, 1H), 4.28 – 3.88 (d, J = 6.7 Hz, 1H), 3.73 – 3.59 (td, J = 6.4, 2.3 Hz, 2H), 1.90 – 1.74 (m, 1H), 1.73 – 1.53 (m, 4H), 1.50 – 1.37 (m, 20H); **^{13}C NMR** ($CDCl_3$, 126 MHz) δ (ppm): 172.2, 155.6, 81.9, 79.8, 62.7, 53.9, 32.9, 32.3, 28.5, 28.2, 21.5; **HRMS** (ESI-TOF) mass calculated for $[2M+Na]^+$ ($C_{30}H_{58}N_2O_{10}Na^+$) expected *m/z* 629.3984; found *m/z* 629.3978.

tert-butyl (S)-7-amino-2-((tert-butoxycarbonyl)amino)heptanoate (11)

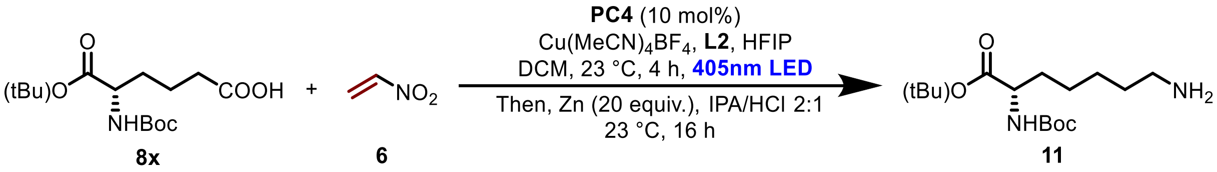

Prepared following a modified general procedure **A**, using carboxylic acid **8x** (1.0 equiv.; 0.117 mmol; 37.0 mg), nitroethylene **6** (1.1 equiv.; 0.129 mmol), **PC4** (0.02 equiv.; 0.012 mmol; 4.8 mg), $Cu(MeCN)_4BF_4$ (0.01 equiv.; 0.01 mmol; 1.9 mg), and **L2** (0.01 equiv.; 0.01 mmol; 1.2 mg) were introduced into a Schlenk tube with a magnetic stirrer bar. The atmosphere was exchanged to argon and degassed, dry DCM (1.2 mL, 0.1 M, previously degassed through 10 min argon sparging) was introduced through a syringe. The reaction mixture was cooled to 0 °C (ice bath) before adding HFIP (0.02 equiv.; 0.012 mmol; 1.2 µL), and nitroethylene **6** (1.1 equiv.; 0.129 mmol). The vessel was sealed and placed in a glass-wall water bath with cooling coil to keep the water temperature between 20 - 25 °C. See Fig. S1 for visual details of the reaction setup. The reaction was irradiated through the bottom glass wall with 405 nm LEDs for 4 h under moderate stirring (450 rpm). The vessel was then removed from the water bath. The crude reaction mixture was passed through a silica plug and rinsed with dichloromethane into a round bottom flask. The solution was concentrated by rotary evaporation and a magnetic stirrer bar was added. The crude residue was dissolved in isopropanol (2 mL) and HCl aq. (1M) (1 mL) was added. Zinc dust (20 equiv.; 130mg) was weighed and added to the reaction flask. The reaction was stirred at room temperature for 16 hours. Upon completion, the solvent was removed by rotary evaporation. Saturated aq. Na_2CO_3, and DCM was added. The organic layer was separated, dried (Na_2SO_4), filtered and concentrated *in vacuo*. The crude residue was purified by flash column chromatography (SiO_2; 8:2 DCM/MeOH; eluent removed using a rotary evaporator, maintaining the water bath at 35 °C and vacuum pressure 130 – 80 mbar) to afford compound **11** (17.8 mg; 48%) as a colourless oil. **IR** (film) ν_{max}/cm^{-1}: 3368, 2977, 2930, 2861, 1712, 1498, 1457, 1391, 1366, 1251, 1154, 1049, 1021, 848; **^{1}H NMR** ($CDCl_3$, 500 MHz) δ (ppm): 5.08 – 4.65 (d, J = 8.6 Hz, 1H), 4.29 – 3.94 (q, J = 7.2 Hz, 1H), 2.71 – 2.64 (t, J = 6.9 Hz, 2H), 1.82 – 1.68 (m, 1H), 1.63 – 1.54 (m, 1H), 1.49 – 1.42 (m, 22H), 1.39 – 1.28 (m, 4H); **^{13}C NMR** ($CDCl_3$, 126 MHz) δ (ppm): 172.1, 155.4, 81.7, 79.6, 53.9, 42.1, 33.6, 33.0, 28.4, 28.0, 26.5, 25.0; **HRMS** (ESI-TOF) mass calculated for $[M+H]^+$ ($C_{16}H_{33}N_2O_4^+$) expected *m/z* 317.2435; found *m/z* 317.2434.

2-((1S,4aS)-7-isopropyl-1,4a-dimethyl-1,2,3,4,4a,9,10,10a-octahydrophenanthren-1-yl)acetic acid (**8z**)

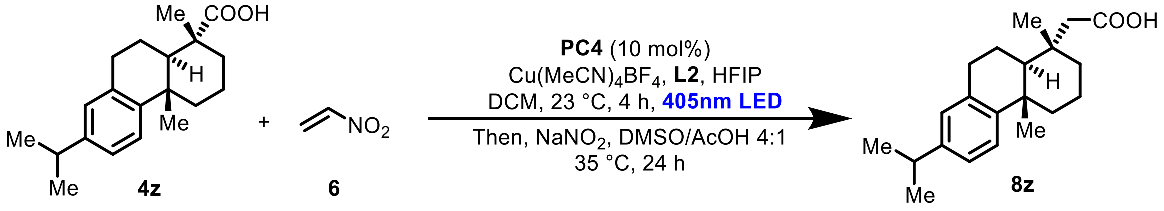

Prepared following general procedure **A**, using Dehydroabietic acid **4z** (1.0 equiv.; 0.2 mmol; 60.0 mg), nitroethylene **6** (1.1 equiv.; 0.21 mmol), **PC4** (0.10 equiv.; 0.02 mmol; 8.2 mg), $Cu(MeCN)_4BF_4$ (0.01 equiv.; 0.01 mmol; 3.2 mg), **L2** (0.01 equiv.; 0.01 mmol; 2.1 mg), HFIP (0.02 equiv.; 0.02 mmol; 2 µL), and $NaNO_2$ (6.0 equiv.; 1.2 mmol; 84 mg). The reaction was worked up according to general workup procedure **(iv)** and the crude residue was purified by flash column chromatography (SiO_2; 100% DCM; eluent removed using a rotary evaporator, maintaining the water bath at 35 °C and vacuum pressure 130 – 80 mbar) to afford compound **8z** (31.4 mg; 50%, >20:1 dr) as a white solid. The relative configuration of the chiral center involved in the reactivity is assigned according to previously reported Giese-type radical addition, assuming analogous substrate control.[12] **$[\alpha]_D^{25}$** = +12 (c = 1.00 CHCl3). **IR** (film) ν_{max}/cm^{-1}: 2927, 1738, 1703, 1497, 1442, 1366, 1264, 1224, 1217, 896, 821, 731, 703; **M.P.** = 127 °C; **^{1}H NMR** ($CDCl_3$, 500 MHz) δ (ppm): 7.19 – 7.14 (d, J = 8.2 Hz, 1H), 7.02 – 6.96 (dd, J = 8.2, 2.1 Hz, 1H), 6.91 – 6.87 (d, J = 2.0 Hz, 1H), 2.96 – 2.77 (m, 3H), 2.32 (s, 2H), 2.31 – 2.24 (dt, J = 12.9, 3.3 Hz, 1H), 1.91 – 1.82 (ddt, J = 13.0, 6.4, 2.3 Hz, 1H), 1.81 – 1.69 (m, 2H), 1.69 – 1.58 (m, 3H), 1.58 – 1.51 (dd, J = 12.2, 2.2 Hz, 1H), 1.46 – 1.36 (td, J = 13.0, 3.8 Hz, 1H), 1.23 (s, 3H), 1.22 (s, 6H), 1.08 (s, 3H); **^{13}C NMR** ($CDCl_3$, 126 MHz) δ (ppm): 178.1, 147.3, 145.7, 134.9, 127.0, 124.4, 124.0, 48.4, 48.3, 38.4, 38.0, 37.6, 37.5, 33.6, 30.4, 25.5, 24.1, 20.8, 19.5, 19.0; **HRMS** (ESI-TOF) mass calculated for $[M-H]^-$ ($C_{21}H_{29}O_2^-$) expected *m/z* 313.2173; found *m/z* 313.2171.

2-((1S,4aS)-7-isopropyl-1,4a-dimethyl-1,2,3,4,4a,9,10,10a-octahydrophenanthren-1-yl)ethan-1-amine (**13**)

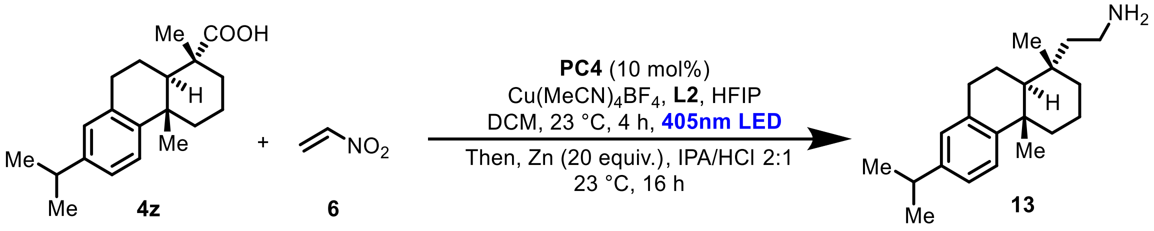

Prepared following a modified general procedure **A**, using dehydroabietic acid **4z** (1.0 equiv.; 0.2 mmol; 60.0 mg), nitroethylene **6** (1.1 equiv.; 0.22 mmol), **PC4** (0.02 equiv.; 0.02 mmol; 8.2 mg), $Cu(MeCN)_4BF_4$ (0.01 equiv.; 0.01 mmol; 3.2 mg), and **L2** (0.01 equiv.; 0.01 mmol; 2.1 mg) were introduced into a Schlenk tube with a magnetic stirrer bar. The atmosphere was exchanged to argon and degassed, dry DCM (2.0 mL, 0.1 M, previously degassed through 10 min argon sparging) was introduced through a syringe. The reaction mixture was cooled to 0 °C (ice bath) before adding HFIP (0.02 equiv.; 0.02 mmol; 2 μL), and nitroethylene **2b** (1.1 equiv.; 0.22 mmol). The vessel was sealed and placed in a glass-wall water bath with cooling coil to keep the water temperature between 20 - 25 °C. See Fig. S1 for visual details of the reaction setup. The reaction was irradiated through the bottom glass wall with 405 nm LEDs for 4 h under moderate stirring (450 rpm). The vessel was then removed from the water bath. The crude reaction mixture was passed through a silica plug and rinsed with dichloromethane into a round bottom flask. The solution was concentrated by rotary evaporation and a magnetic stirrer bar was added. The crude residue was dissolved in isopropanol (2 mL) and HCl aq. (1M) (1 mL) was added. Zinc dust (20 equiv.; 130mg) was weighed and added to the reaction flask. The reaction was stirred at room temperature for 16 hours. Upon completion, the solvent was removed by rotary evaporation. Saturated aq. Na_2CO_3, and DCM was added. The organic layer was separated, dried (Na_2SO_4), filtered and concentrated *in vacuo*. The crude residue was purified by flash column chromatography (SiO_2; 9:1 DCM/MeOH; eluent removed using a rotary evaporator, maintaining the water bath at 35 °C and vacuum pressure 130 – 80 mbar) to afford compound **13** (28.2 mg; 47%, >20:1 dr) as a colourless oil that crystallizes on standing. The relative configuration of the chiral center involved in the reactivity is assigned according to previously reported Giese-type radical addition, assuming analogous substrate control.[12] $[\alpha]_D^{25}$ = +46 (c = 1.00 CHCl3). **IR** (film) ν_{max}/cm^{-1}: 2957, 2925, 2866, 1572, 1497, 1459, 1381, 1057, 883, 821; **^{1}H NMR** ($CDCl_3$, 500 MHz) δ (ppm): 7.19 – 7.14 (d, J = 8.2Hz, 1H), 7.02 – 6.95 (dd, J = 8.2, 2.1 Hz, 1H), 6.91 – 6.86 (d, J = 2.0 Hz, 1H), 2.96 – 2.75 (m, 3H), 2.74 – 2.66

(t, J = 7.9 Hz, 1H), 2.35 – 2.18 (m, 3H), 1.88 – 1.79 (m, 1H), 1.78 – 1.58 (m, 3H), 1.55 – 1.46 (m, 2H), 1.42 – 1.29 (m, 5H), 1.23 (s, 3H), 1.22 (s, 3H), 1.20 (s, 3H), 0.95 (s, 3H); **^{13}C NMR** ($CDCl_3$, 126 MHz) δ (ppm): 147.7, 145.7, 134.9, 127.0, 124.4, 124.0, 48.2, 47.6, 38.7, 37.72, 37.66, 37.1, 35.8, 33.6, 30.5, 25.5, 24.1, 20.9, 19.1, 19.0; **HRMS** (ESI-TOF) mass calculated for $[M+H]^+$ ($C_{21}H_{34}N^+$) expected *m/z* 300.2686; found *m/z* 300.2683.

Mechanistic Insights

Spectroscopic investigation of the photoactive species

a) ¹H NMR studies

¹H NMR studies were conducted using a standard Norell® Select Series™ 5 mm NMR tube. Solutions were prepared at the desired concentration in $CDCl_3$ using a standard volumetric flask and were analysed immediately at room temperature (20 °C). Additions of tetrahydropyran carboxylic acid **4a** were performed directly to the NMR tube using a Hamilton micro syringe.

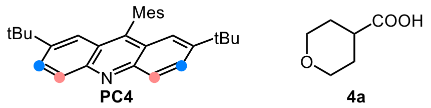

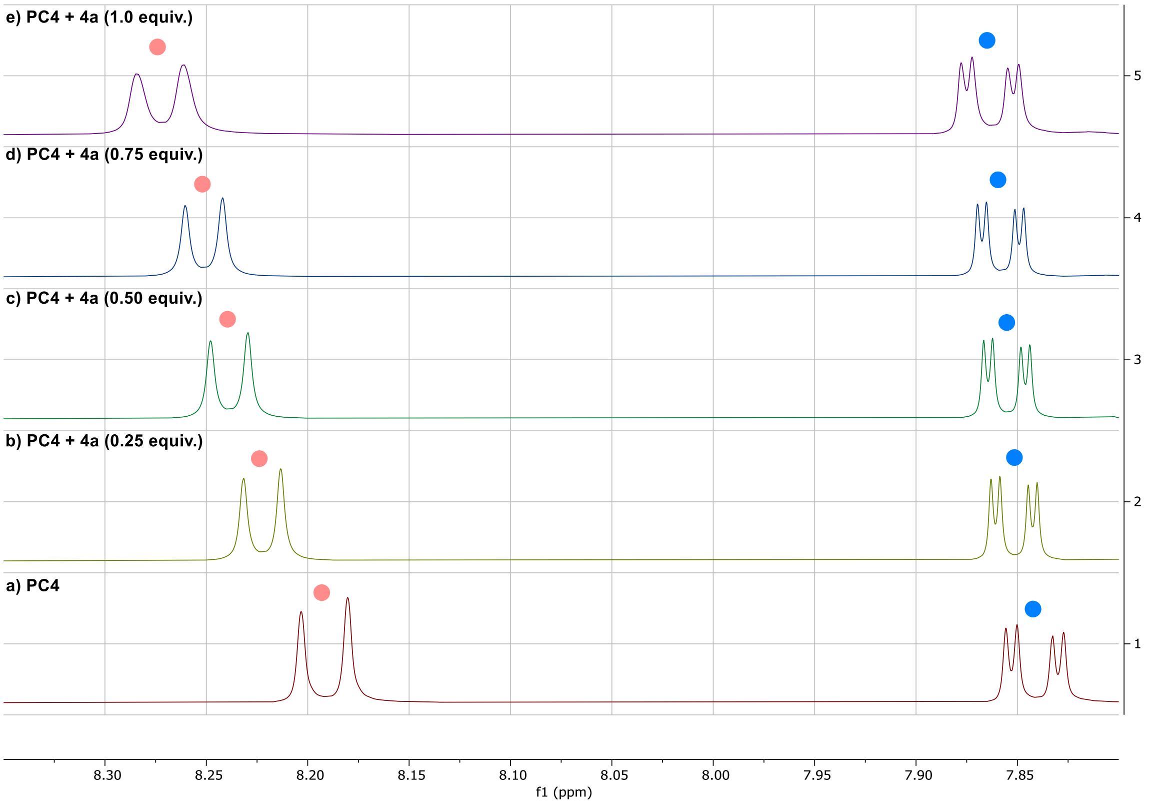

Supplementary Figure S3. Magnification of aromatic region of ¹H NMR spectra of acridine photocatalyst **PC4** ([**PC4**] = 0.01 M in $CDCl_3$) in the presence of increasing concentrations of substrate **4a**. a) no **4a** added; b) 0.25 equiv. **4a** added; c) 0.50 equiv. **4a** added; d) 0.75 equiv. **4a** added; e) 1.0 equiv. **4a** added.

A detectable shift was observed in the ¹H NMR spectrum of the acridine photocatalyst **PC4** upon addition of increasing amounts of tetrahydropyran carboxylic acid **4a** (Supplementary Figure S3). In organic solvents, proton transfer between carboxylic acids and acridine bases is thermodynamically unfavorable at such an extent that any ionization equilibrium is negligible (acridine $pK_{a(B\text{-}H^+)}$ = 12.7 in MeCN, acetic acid pK_a = 21.6 in MeCN).[13,14] Thus, the shift

observed can be rationalized by H-bonding between **4a** and **PC4**. Such pre-complexation is in agreement with the operation of a PCET mechanism.

b) Control experiments with acridinium catalysts

To further support a PCET as the radical generation event, a control experiment was performed subjecting photocatalyst **PC2** under the reaction conditions as below.

Acridinium catalyst **PC2** (0.1 equiv.; 0.01 mmol; 4.0 mg) was employed in the reaction under standard conditions with tetrahydropyran carboxylic acid **4a** (1.0 equiv.; 0.1 mmol; 13.0 mg), nitroethylene **6** (1.1 equiv.; 0.11 mmol), $Cu(MeCN)_4BF_4$ (0.005 equiv.; 0.005 mmol; 1.6 mg), **L2** (0.005 equiv.; 0.005 mmol; 1.04 mg), and HFIP (0.10 equiv.; 0.01 mmol; 1 µL). The reaction yielded no nitroalkane **7a**.

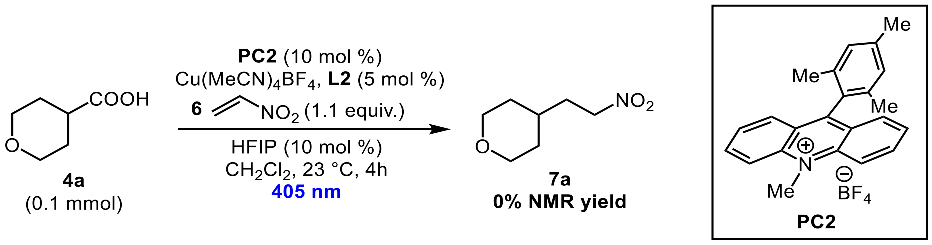

Reaction performed in 0.1 mmol scale, using **4a** (1.0 equiv.), **6** (1.1 equiv.), [**4a**]$_0$ = 0.1 M. ^{1}H-NMR yield using mesitylene as internal standard.

Given that **PC2** is a strong oxidant which lacks the molecular requirements to form an H-bonded complex and undergo a PCET process with a carboxylic acid, the lack of reactivity observed is in agreement with the proposed mechanism.

c) UV/vis absorption studies

General procedure for UV-vis absorption experiments

Absorption spectra were obtained on a UV-vis NIR Agilent Cary Spectrometer. Solutions were prepared at the specified concentrations using dry, spectrophotometric grade dichloromethane (CH_2Cl_2) and the spectra were recorded in Hellma Analytics high precision cell quartz glass cuvettes, 1 cm path length. To all measurements, a blank sample was subtracted.

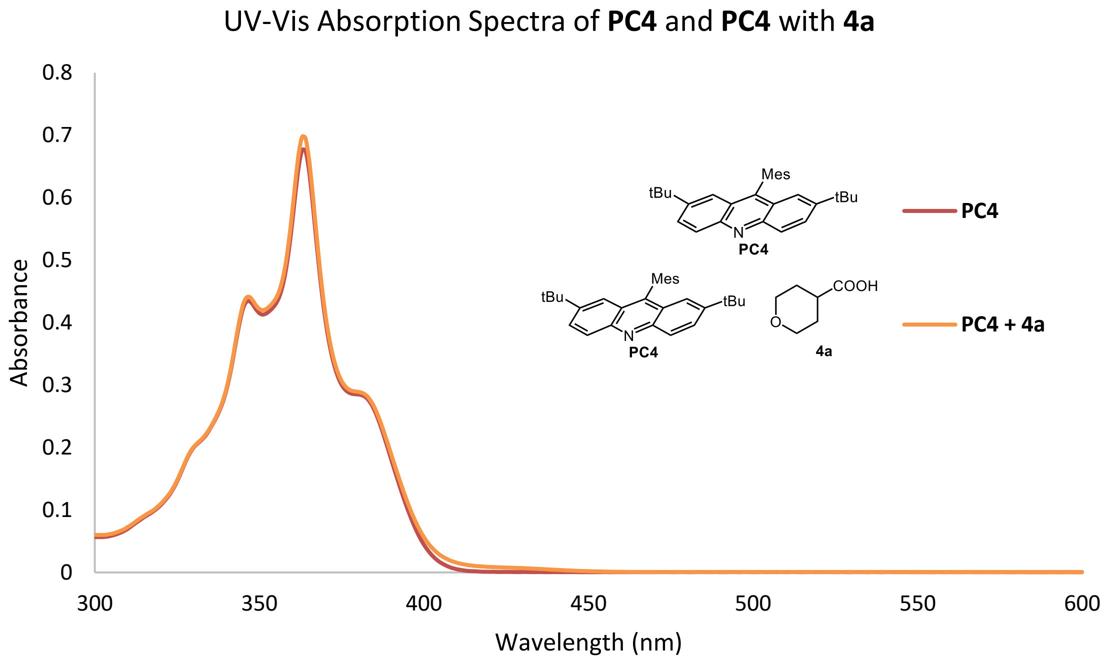

Supplementary Figure S4. Absorption spectra of acridine photocatalyst **PC4** in dichloromethane at $5.0x10^{-5}$ M (red line) and a mixture of acridine photocatalyst **PC4** ($5.0x10^{-5}$ M) and tetrahydropyran carboxylic acid **4a** ($5.0x10^{-4}$ M) in dichloromethane (orange line).

The UV-vis spectrum of a CH_2Cl_2 solution of the acridine photocatalyst shows absorption maxima in the near UV, with shoulder fading in the visible region (400 – 410 nm, Supplementary Figure S4, red line). Upon addition of acid substrate **4a** (10:1, according to reaction stoichiometry), slight spectral variations were observed (orange line) and a bathochromic shift of the residual absorption in the visible region is observed. Analogous bathochromic shifts have been observed in previous reports upon mixing acridine with carboxylic acids and are ascribable to the formation of photoactive H-bonded complexes between the two species.[15]

d) Reactivity versus wavelength studies

Reactions were set up according to general procedure **A**, with tetrahydropyran carboxylic acid **4a** (1.0 equiv.; 0.1 mmol; 13.0 mg), nitroethylene **6** (1.1 equiv.; 0.11 mmol), **PC4** (0.10 equiv.; 0.01 mmol; 4.1 mg), $Cu(MeCN)_4BF_4$ (0.005 equiv.; 0.005 mmol; 1.6 mg), **L2** (0.005 equiv.; 0.005 mmol; 1.04 mg), HFIP (0.10 equiv.; 0.01 mmol; 1 µL). Refer to section: **LEDs and photoreactor specifications** for information on the light sources used at 440 nm and 456 nm.

A)

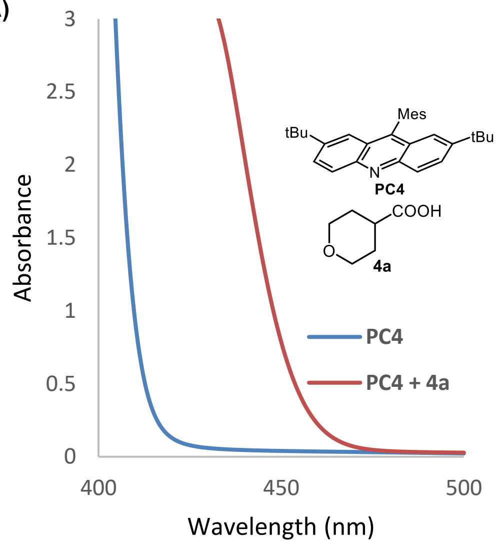

B)

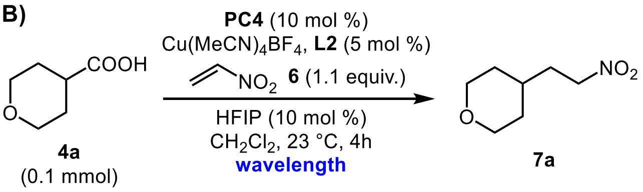

Entry[a]	Wavelength (nm)	Yield 7a (%)[b]
1	**405**	95
2	**440**	50
3	**456**	37

[a] Reactions performed in 0.1 mmol scale, using **4a** (1.0 equiv.), **6** (1.1 equiv.) $[\mathbf{4a}]_0$ = 0.1 M. [b] Unless otherwise stated, ^{1}H-NMR yield using mesitylene as internal standards.

Supplementary Figure S5. A) Magnification of the visible region of a UV-vis absorption spectrum of the mixture of acridine photocatalyst **PC4** (1.0×10^{-2} M) and carboxylic acid **4a** (1.0×10^{-1} M), at reaction concentrations, in CH_2Cl_2. **B)** Reactivity assessment at different wavelengths. The wavelengths reported corresponds to the emission maximum of the LED source used.

A clear trend of reactivity is observed by varying the wavelength used to perform the reaction. While almost quantitative yield of intermediate nitroalkane is observed at 405 nm, irradiation at 440 nm and 456 nm led to decreased yields (Supplementary Figure S5, B). The trend is consonant with the absorption profile observed in a solution of photocatalyst **PC4** and carboxylic acid substrate **4a** at operating reaction concentrations (Supplementary Figure S5, A). The formation of product at 456 and 440 nm, where H-bonded complex between **PC4** and **4a** can absorb light but absorption of **PC4** alone is minimal, suggests that the photochemical event occurs from excitation of the H-bonded complex,[15] leading to PCET radical generation (see also ref. 60–64, main text).

Investigation of the role of Cu/ligand cocatalyst system

To investigate the role of the copper/ligand cocatalyst system in the reaction, control experiments were run (below).

a) Assessing the role of the copper ligand

To probe the role of the ligand under standard reaction conditions, the decarboxylative Giese addition to nitroethylene was assessed in the presence and absence of neocuproine **L2**.

Supplementary Table S8. Investigation of the role of the ligand

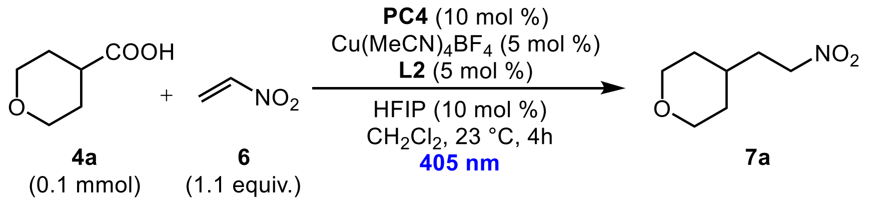

Entry[a]	Ligand (5 mol %)	Yield 7a (%)[b]
1	**L2**	95
2	**No ligand**	75

[a] Reactions performed in 0.1 mmol scale, using **4a** (1.0 equiv.), **6** (1.1 equiv.) $[\mathbf{4a}]_0 = 0.1$ M.

[b] Unless otherwise stated, ^{1}H-NMR yield using mesitylene as internal standards.

While the optimal conditions that include **L2** afford **7a** in 95% NMR yield (Table S8, entry 1), only a 20% yield decrease was observed in the total absence of ligands (entry 2). Precipitates were observed after irradiation, in the reaction when the ligand was omitted. The results above suggest that the ligand does not play a crucial role in this reactivity, but it is likely enhancing stability of the metal co-catalyst throughout the reaction.

b) Investigating the possible role of copper as a Lewis acid

Experiments to assess the possibility of copper acting as a Lewis acid for nitroethylene activation were conducted. $Zn(OTf)_2$ or $Ni(OTf)_2$ were employed under standard reaction conditions in 5 mol % (Table S9, entry 1 and 2) with tetrahydropyran carboxylic acid **4a** (1.0 equiv.; 0.1 mmol; 13.0 mg), nitroethylene **6** (1.1 equiv.; 0.11 mmol), **PC4** (0.10 equiv.; 0.01 mmol; 4.1 mg), **L2** (0.005 equiv.; 0.005 mmol; 1.04 mg), HFIP (0.10 equiv.; 0.01 mmol; 1 µL).

Supplementary Table S9. Investigation of other Lewis acids

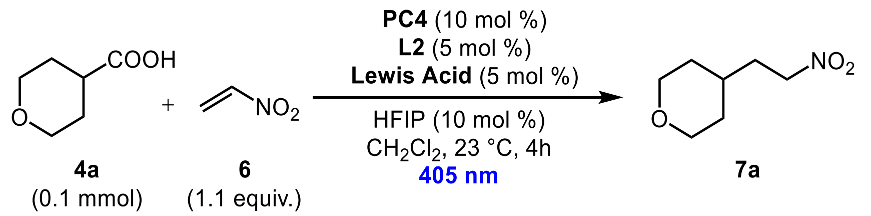

Entry[a]	Lewis Acid (5 mol %)	Yield 7a (%)[b]
1	**$Zn(OTf)_2$**	14
2	**$Ni(OTf)_2$**	22
3	**None/No L2**	23
4	**Standard conditions, $Cu(MeCN)_4BF_4$**	95

[a] Reactions performed in 0.1 mmol scale, using **4a** (1.0 equiv.), **6** (1.1 equiv.) $[\mathbf{4a}]_0 = 0.1$ M.
[b] Unless otherwise stated, ^{1}H-NMR yield using mesitylene as internal standards.

When the copper co-catalyst was replaced by other Zn or Ni Lewis acids, a significant reduction in yield of the nitroalkane **7a** was observed (Table S9, entries 1 and 2) compared to 95% yield with $Cu(MeCN)_4BF_4$ (entry 4). Under these conditions, the yield is comparable to that obtained in the total absence of transition metal co-catalysts (entry 3, 23% NMR yield), suggesting that a Lewis acidic role of the copper catalyst in the reaction is unlikely.

c) UV-vis absorption studies to investigate the possible role of copper in the photoactivation

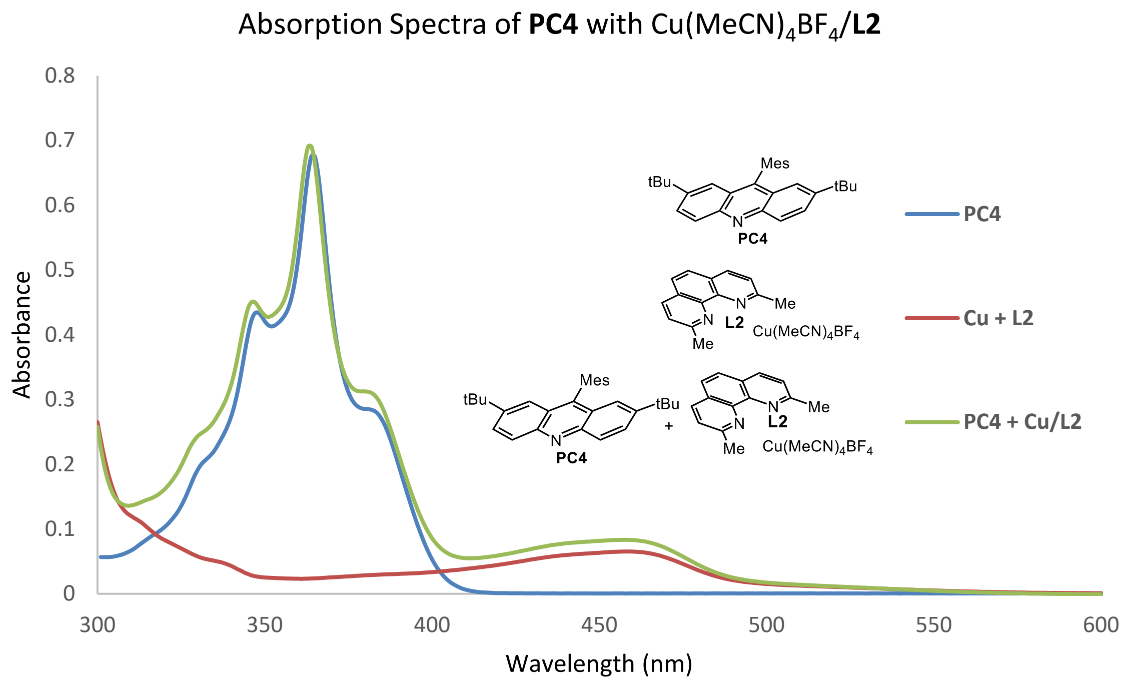

Supplementary Figure S6. Absorption spectra of acridine photocatalyst (**PC4**) in dichloromethane at 5.0x10^{-5} M (**blue**), Cu(MeCN)$_4$BF$_4$/**L2** in dichloromethane at 2.5x10^{-5} M (**red**), and acridine photocatalyst (**PC4**) in dichloromethane at 5.0x10^{-5} M in the presence of Cu(MeCN)$_4$BF$_4$/**L2** (0.5 equiv., 2.5x10^{-5} M) (**green**).

Upon analyzing a mixture of acridine catalyst and copper co-catalyst, minimal or no spectral variations were observed in the UV region (variation in absorption are mostly due to the additive effect of the single components). A weak absorption band with maximum in the visible region (440 - 460 nm) was observed, due to the formation of copper complexes. It is relevant to recall that control experiments performed at different wavelengths showed considerable reduction in yield when the reaction was performed at these wavelengths (see Supplementary Figure S5, B), and the reactivity was instead observed to reflect the absorption band of the acridine-substrate complex. These results suggest that the copper catalyst used is likely not involved in the photoactivation event but plays a role in following steps of the mechanistic manifold.

Probing the operation of a Cu(I/II) catalytic cycle

Both Cu(0/I) and Cu(I/II) catalytic cycles have been invoked in previous examples of acridine photocatalysis.[16,17] The type of operating catalytic cycle may be dictated by the nature of the radical trap and the ligand used (the use of electron-rich aliphatic amines as ligands have been proposed to facilitate the generation of copper(0) species in these reactions).[16] Therefore, we performed control experiments to investigate the operation of a Cu(0/I) or a Cu(I/II) catalytic cycle in our reaction.

a) Mercury drop test

Reactions were conducted using standard conditions with 1 g of mercury added, tetrahydropyran carboxylic acid **4a** (1.0 equiv.; 0.15 mmol; 19.5 mg), nitroethylene **6** (1.1 equiv.; 0.17 mmol), **PC4** (0.10 equiv.; 0.015 mmol; 6.2 mg), $Cu(MeCN)_4BF_4$ (0.005 equiv.; 0.008 mmol; 2.4 mg), **L2** (when used: 0.005 equiv.; 0.008 mmol; 1.6 mg), HFIP (0.10 equiv.; 0.015 mmol; 1.5 µL).

Supplementary Table S10. Mercury Drop Test

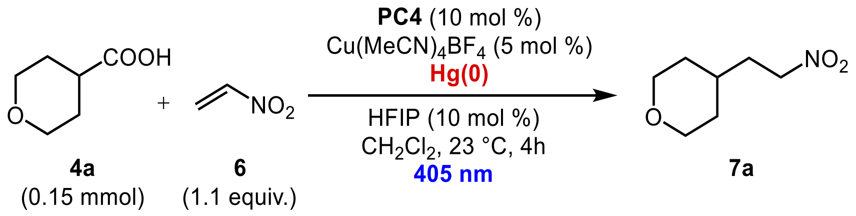

Entry[a]	Ligand (5 mol %)	Hg(0) (equiv.)	Yield 7a (%)[b]
1	**L2**	**3.3**	92
2	**L2**	**0**	95
3	**None**	**3.3**	74
4	**None**	**0**	75

[a] Reactions performed in 0.15 mmol scale, using **4a** (1.0 equiv.), **6** (1.1 equiv.) $[\mathbf{4a}]_0$ = 0.1 M.
[b] Unless otherwise stated, ^{1}H-NMR yield using mesitylene as internal standards.

In previous reports, the observation of a decreased reactivity in a mercury drop test was presented as evidence for Cu(0) species participating in copper-acridine photocatalysis.[16]

Running our reaction in the presence or in the absence of mercury (Table S10) led to comparable yields of product **7a** (both in the presence or in the absence of neocuproine ligand). These results point against the involvement of heterogeneous Cu(0) species in this reactivity.

b) Reactivity with other Cu species

$Cu(OTf)_2$ or Cu(0) sources were employed under standard reaction conditions in 5 mol % (Table S11) with tetrahydropyran carboxylic acid **4a** (1.0 equiv.; 0.1 mmol; 13.0 mg), nitroethylene **6** (1.1 equiv.; 0.11 mmol), **PC4** (0.10 equiv.; 0.01 mmol; 4.1 mg), **L2** (0.005 equiv.; 0.005 mmol; 1.04 mg), HFIP (0.10 equiv.; 0.01 mmol; 1 µL). When Cu(0) nanopowder was used, solids were weighed in a glove box.

Supplementary Table S11. Reactivity with other Cu species

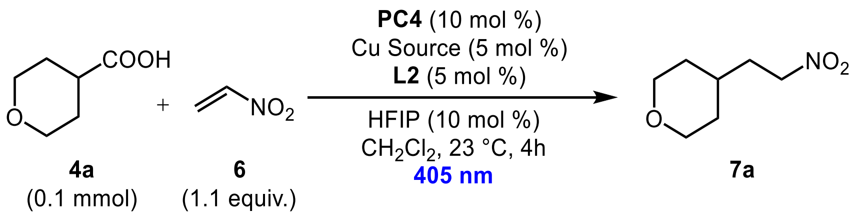

Entry[a]	Cu Source (5 mol %)	Yield 7a (%)[b]
1	**Cu(0) nanopowder, 25 nm particle size (TEM)**	20
2	**Cu(0) powder (150 mesh)**	23
3	**$Cu(OTf)_2$**	96
4	**Standard conditions, $Cu(MeCN)_4BF_4$**	95
5	**None**	23

[a] Reactions performed in 0.1 mmol scale, using **4a** (1.0 equiv.), **6** (1.1 equiv.) [**4a**]$_0$ = 0.1 M.
[b] Unless otherwise stated, ^{1}H-NMR yield using mesitylene as internal standards.

In previous reports, the observation of efficient reactivity using copper(0) nanopowder, 25 nm particle size (TEM), as co-catalyst was suggested as evidence for a Cu(0/I) catalytic cycle.[16]
Running our standard reaction with the same or other Cu(0) sources led to product **7a** in yields which are comparable to experiments run in the absence of a copper catalyst (compare entries 1, 2 with entry 5). In contrast, $Cu(OTf)_2$ was found to be an equally effective catalyst compared to our optimal catalyst $Cu(CH_3CN)_4BF_4$, suggesting that Cu(II) species are likely to be involved in the catalytic cycle, and can act as an efficient catalyst for this reaction.

Taken together, the control experiments run above support the involvement of a Cu(I/II) catalytic cycle in this reactivity, as presented in the proposed mechanism below (Supplementary Figure S7).

Proposed Catalytic Cycle

Based on the results of the mechanistic experiments outlined above and literature precedents (see ref. 60–64, main text), a proposed mechanism for the acridine promoted decarboxylative Giese addition to nitroethylene is shown below.

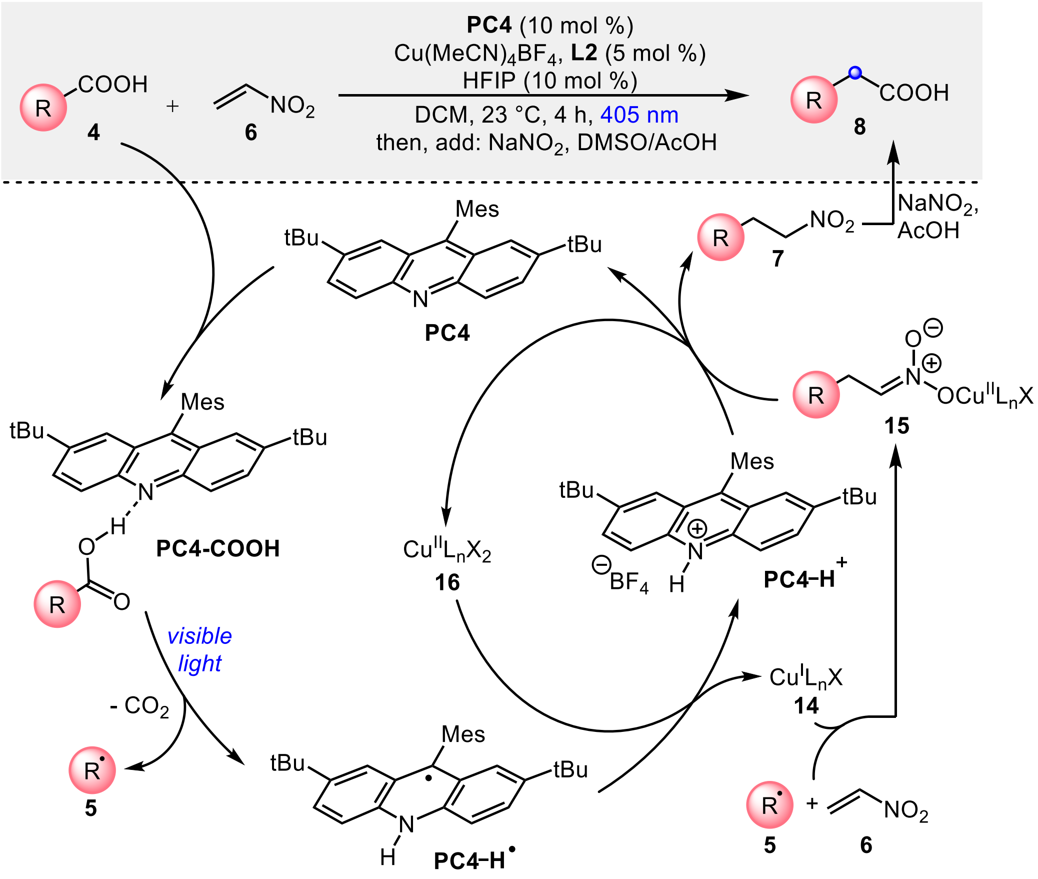

Supplementary Figure S7. Proposed Catalytic Cycle

Following formation of H-bonded complex **PC4-COOH**, visible light-induced PCET leads to the decarboxylative generation of radical **5** and to reduced photocatalyst **PC4-H•**. Carbon-centered radical **5** then engages with nitroethylene **6** in the presence of the Cu(I) co-catalyst **14** leading to Cu(II)-nitronate **15**. Protonation of this species by **PC4-H**$^+$—generated upon oxidation of **PC4-H•** by Cu(II) species **16**—closes the catalytic cycle and affords nitroalkane **7** (generation of **16** in the first catalytic turnover is proposed either by disproportionation of Cu(I) pre-catalyst or by protonation of **15** by the carboxylic acid substrate **4**). Intermediate nitroalkane **7** is finally converted to desired carboxylic acid homologue **8** upon *in-situ* treatment with $NaNO_2$/AcOH.

References and Notes

[1] Zhilyaev, K. A.; Lipilin, D. L.; Kosobokov, M. D.; Samigullina, A. I.; Dilman, A. D. Preparation and Evaluation of Sterically Hindered Acridine Photocatalysts. *Adv. Synth. Catal.* **2022**, *364*, 3295-3301.

[2] Lin, Y.-H.; Lai, C.-C.; Liu, Y.-H.; Peng, S.-M.; Chiu, S.-H. Sodium Ions Template the Formation of Rotaxanes from BPX26C6 and Nonconjugated Amide and Urea Functionalities. *Angew. Chem. Int. Ed.* **2013**, *52*, 10231-10236.

[3] Flood, D. T.; Zhang, X.; Fu, X.; Zhao, Z.; Asai, S.; Sanchez, B. B.; Sturgell, E. J.; Vantourout, J. C.; Richardson, P.; Flanagan, M. E.; Piotrowski, D. W.; Kölmel, D. K.; Wan, J.; Tsai, M.-H.; Chen, J. S.; Baran, P. S.; Dawson, P. E. RASS-Enabled S/P-C and S-N Bond Formation for DEL Synthesis. *Angew. Chem. Int. Ed.* **2020**, *59*, 7377-7383.

[4] Ganivada, M. N.; Kumar, V. R. N. P.; Bhattacharya, S.; Shunmugam, R. Efficient Approach to Produce Multi-Functional Copolymers for Effective DNA Binding. *Polym. Adv. Technol.* **2017**, *28*, 271-280.

[5] Chi, Y.; Guo, L.; Kopf, N. A.; Gellman, S. H. Enantioselective Organocatalytic Michael Addition of Aldehydes to Nitroethylene: Efficient Access to γ^2-Amino Acids. *J. Am. Chem. Soc.* **2008**, *130*, 5608-5609.

[6] Chu, L.; Ohta, C.; Zuo, Z.; MacMillan, D. W. C. Carboxylic Acids as A Traceless Activation Group for Conjugate Additions: A Three-Step Synthesis of (±)-Pregabalin. *J. Am. Chem. Soc.* **2014**, *136*, 10886–10889.

[7] Ramirez, N. P.; Gonzalez-Gomez, J. C. Decarboxylative Giese-Type Reaction of Carboxylic Acids Promoted by Visible Light: A Sustainable and Photoredox-Neutral Protocol. *Eur. J. Org. Chem.* **2017**, *2017*, 2154–2163.

[8] Zhang, O.; Schubert, J. W. Derivatization of Amino Acids and Peptides via Photoredox-Mediated Conjugate Addition. *J. Org. Chem.* **2020**, *85*, 6225–6232.

[9] Qin, T.; Malins, L. R.; Edwards, J. T.; Merchant R. R.; Novak, A. J. E.; Zhong, J. Z.; Mills, R. B.; Yan, M.; Yuan, C.; Eastgate, M. D.; Baran, P. S. Nickel-Catalyzed Barton Decarboxylation and Giese Reactions: A Practical Take on Classic Transforms. *Angew. Chem. Int. Ed.* **2016**, *56*, 260-265.

[10] Ribic, R.; Habjanec, L.; Vranesic, B.; Frkanec, R.; Tomic, S. Synthesis and Immunostimulating Properties of Novel Adamant-1-yl Tripeptides. *Chemistry and Biodiversity.* **2012**, *9*, 777-788.

[11] Tilvawala, R.; Cammarata, M.; Adediran, S. A.; Brodbelt, J. S.; Pratt, R. F. A New Covalent Inhibitor of Class C β-Lactamases Reveals Extended Active Site Specificity. *Biochemistry.* **2015**, *54*, 7375-7384.

[12] Noble, A.; Mega, R. S.; Pflasterer, D.; Myers, E. L.; Aggarwal, V. K. Visible-Light-Mediated Decarboxylative Radical Additions to Vinyl Boronic Esters: Rapid Access to γ-Amino Boronic Esters. *Angew. Chem. Int. Ed.* **2018**, *57*, 2155-2159.

[13] Lõkov, M.; Tshepelevitsh, S.; Heering, A.; Plieger, P. G.; Vianello, R.; Leito, I. On the Basicity of Conjugated Nitrogen Heterocycles in Different Media. *Eur. J. Org. Chem.* **2017**, *2017*, 4475-4489.

[14] Kozak, A.; Czaja, M.; Chmurzyński, L. Investigations of (acid + base) equilibria in systems modelling interactions occurring in biomolecules. *J. Chem. Thermodynamics.* **2006**, *38*, 599-605.

[15] Okada, K.; Okubo, K.; Oda, M. A simple and convenient photodecarboxylation method of intact carboxylic acids in the presence of aza aromatic compounds. *J. Photochem. Photobiol. A: Chem.* **1991**, *57*, 265–277.

[16] Dang, H. T.; Haug, G. C.; Nguyen, V. T.; Vuong, N. T. H.; Nguyen, V. D.; Arman, H. D.; Larionov, O. V. Acridine Photocatalysis: Insights into the Mechanism and Development of a Dual-Catalytic Direct Decarboxylative Conjugate Addition. *ACS Catal.* **2020**, *10,* 11448–11457

[17] Bhatt, K.; Adili, A.; Tran, A. H.; Elmallah, K. M.; Ghiviriga, I.; Seidel, D. Photocatalytic Decarboxylative Alkylation of Cyclic Imine–BF3 Complexes: A Modular Route to Functionalized Azacycles. *J. Am. Chem. Soc.* **2024**, *146*, 26331–26339.

NMR Spectra

6-phenylhexanoic acid (8b)

^{1}H-NMR ($CDCl_3$, 500 MHz)

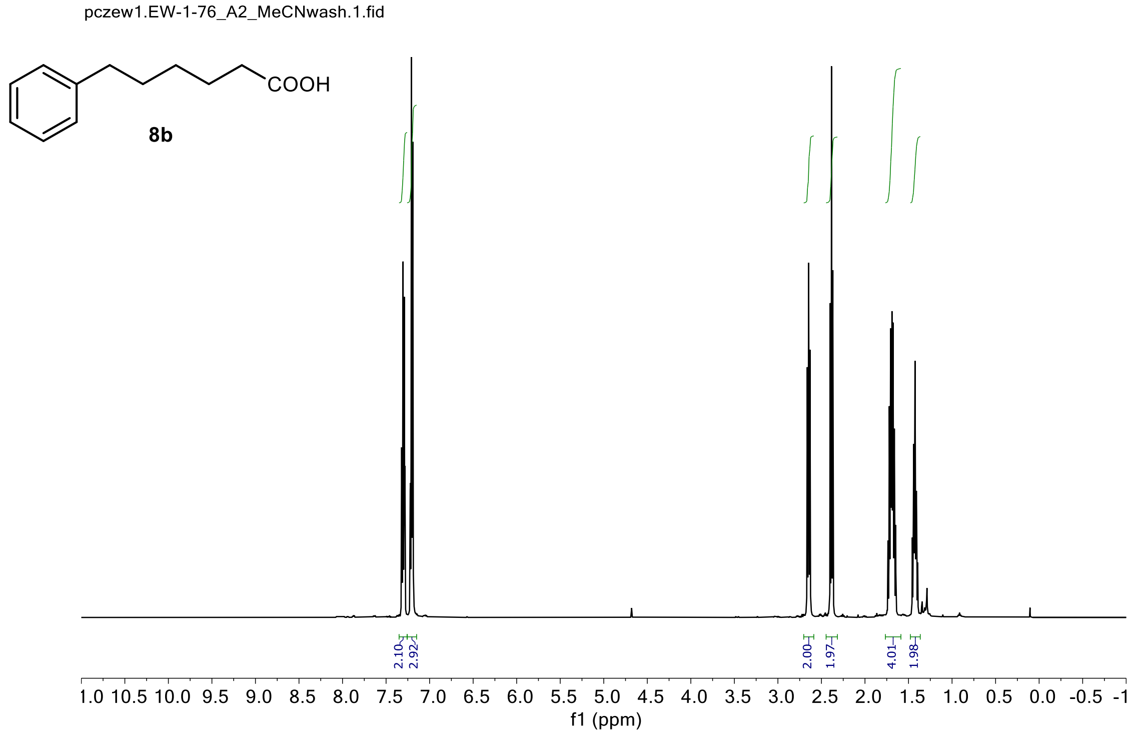

^{13}C-NMR ($CDCl_3$, 126 MHz)

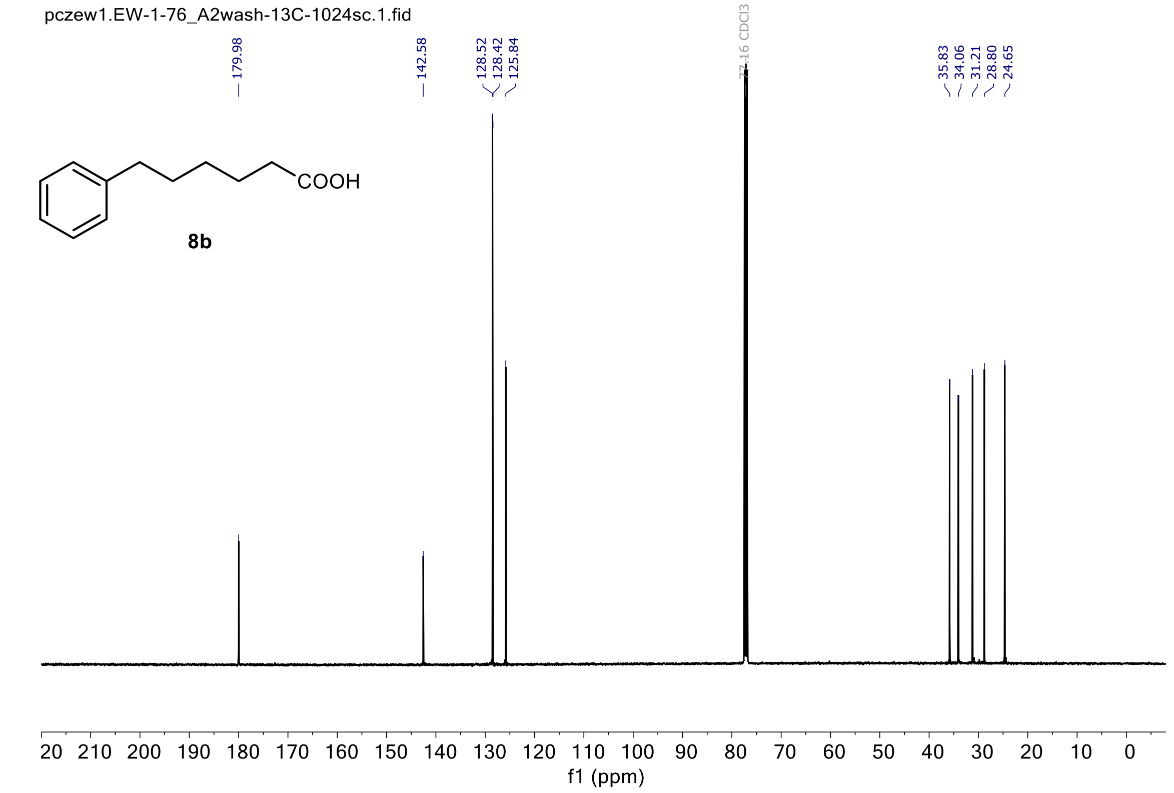

11-hydroxyundecanoic acid (8c)

^{1}H-NMR ($CDCl_3$, 500 MHz)

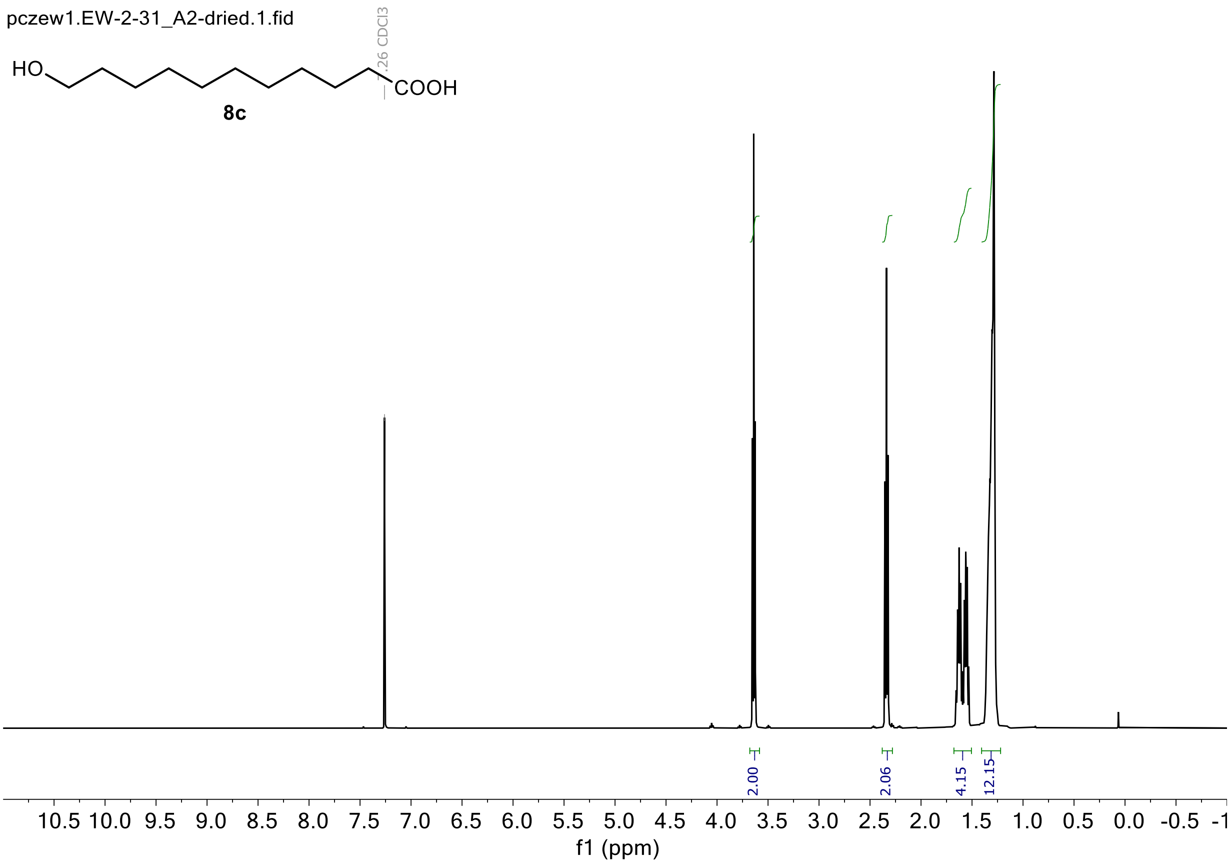

^{13}C-NMR ($CDCl_3$, 126 MHz)

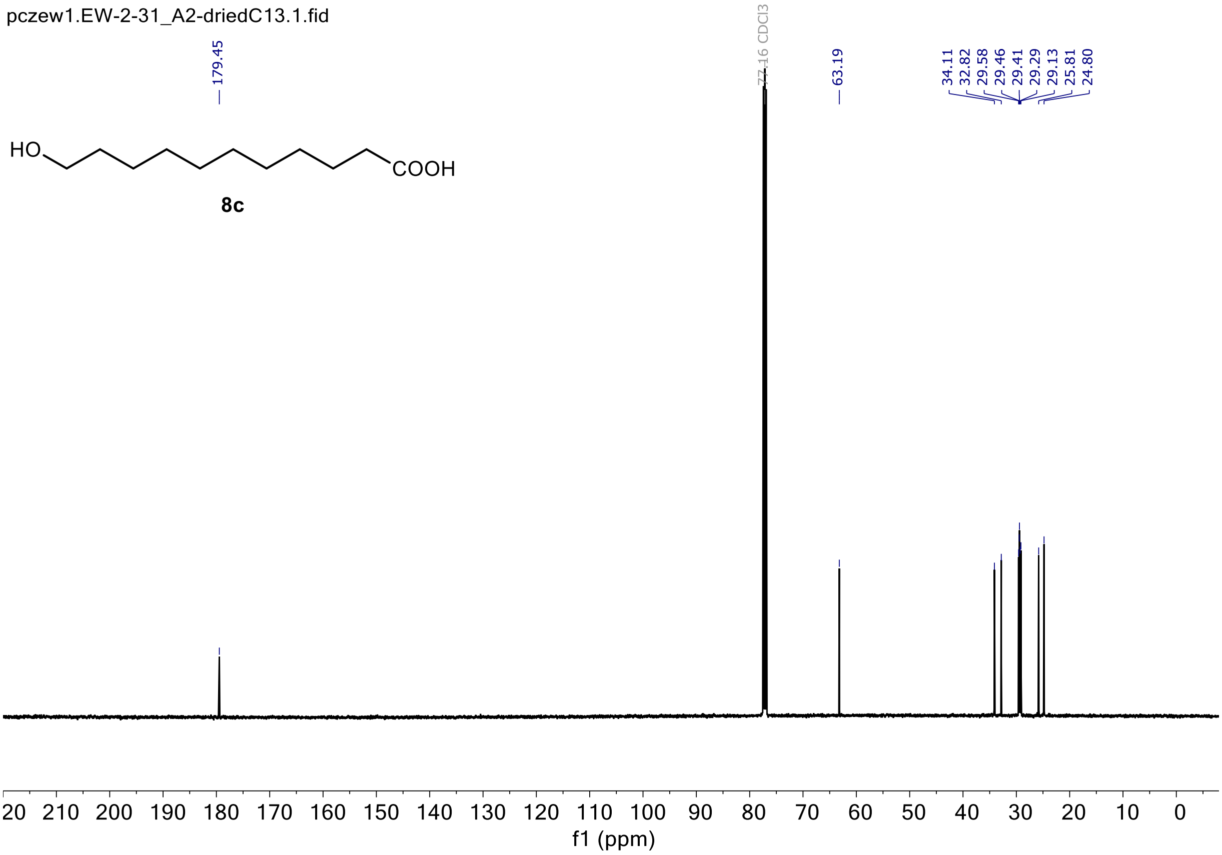

7-chloroheptanoic acid (8d)

^{1}H-NMR ($CDCl_3$, 400 MHz)

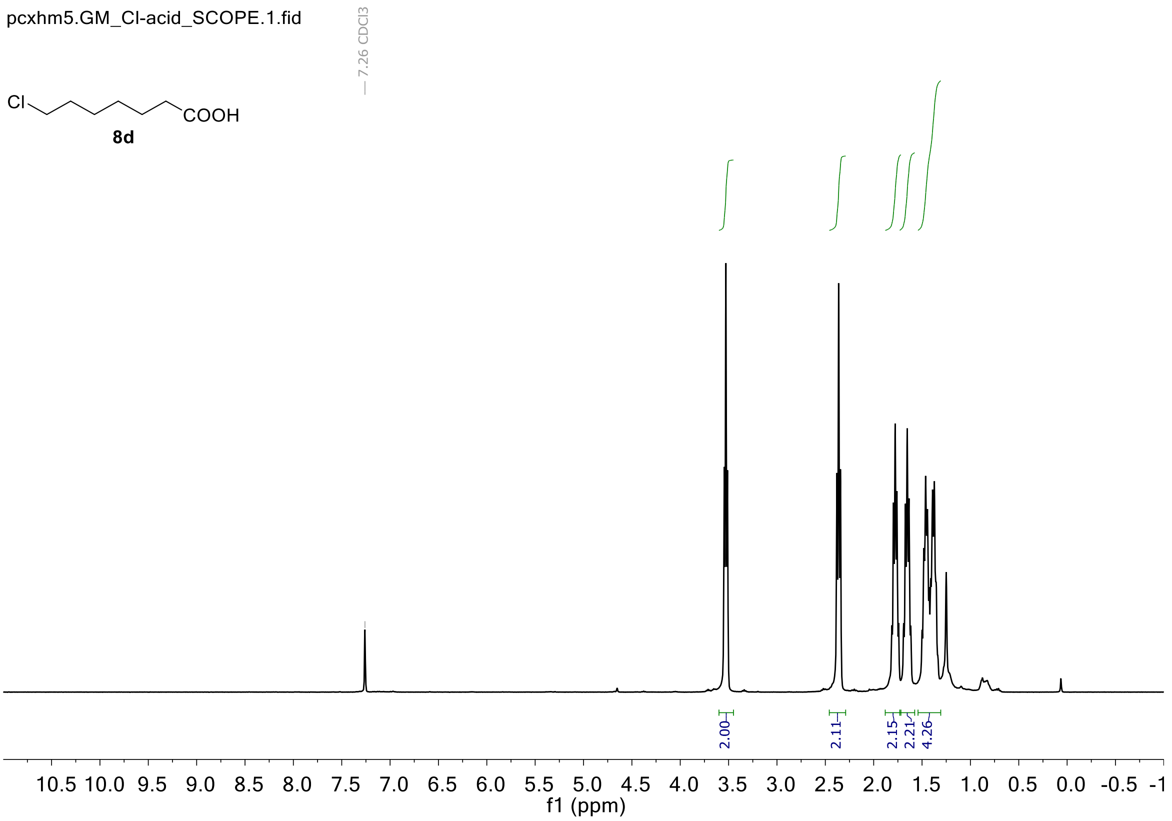

^{13}C-NMR ($CDCl_3$, 101 MHz)

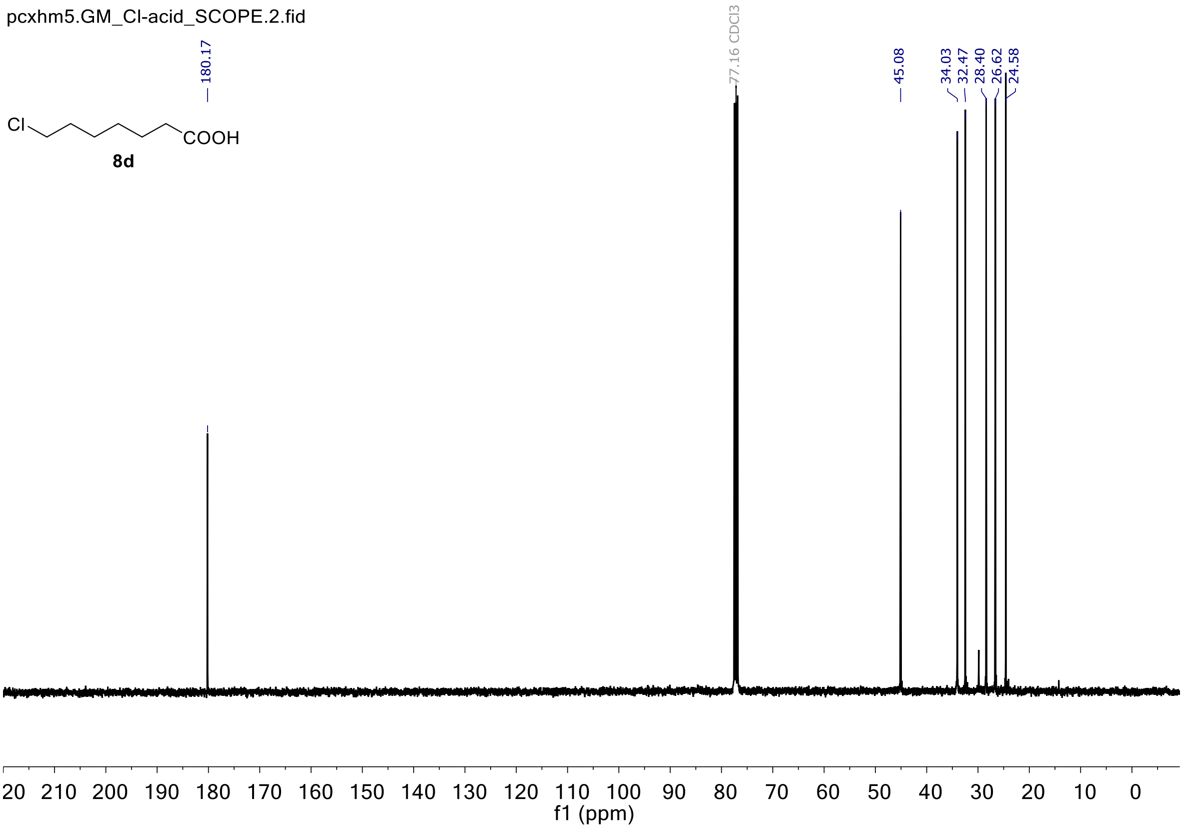

dodec-11-enoic acid (8e)

^{1}H-NMR ($CDCl_3$, 500 MHz)

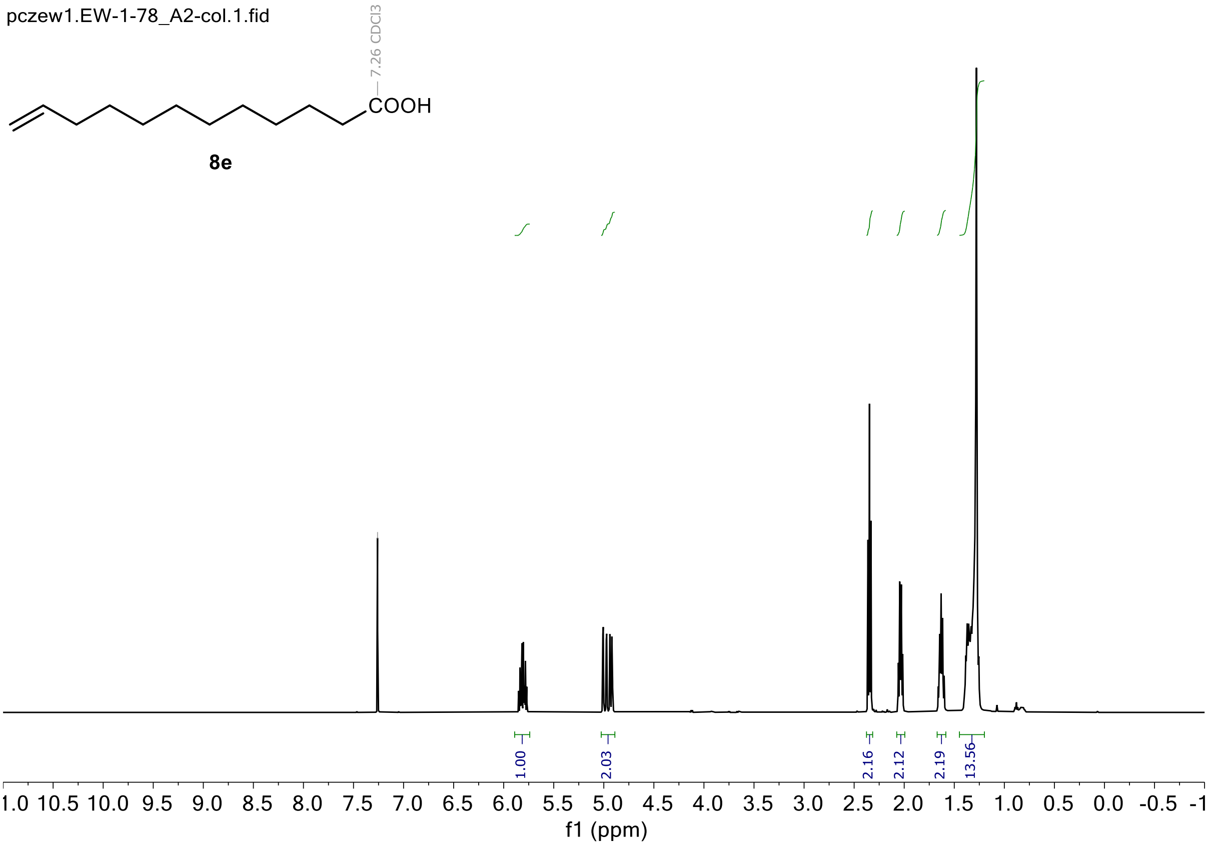

^{13}C-NMR ($CDCl_3$, 126 MHz)

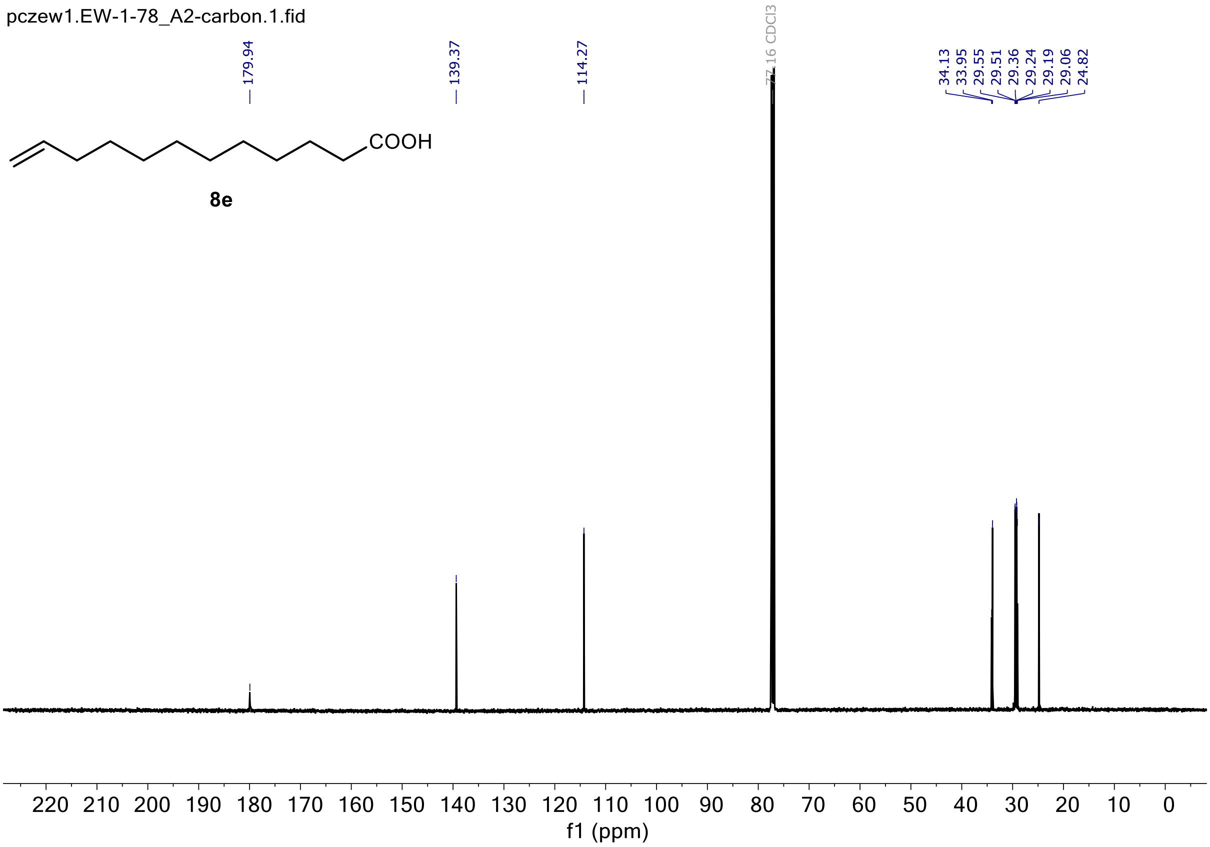

dodec-11-ynoic acid (8f)

^{1}H-NMR ($CDCl_3$, 500 MHz)

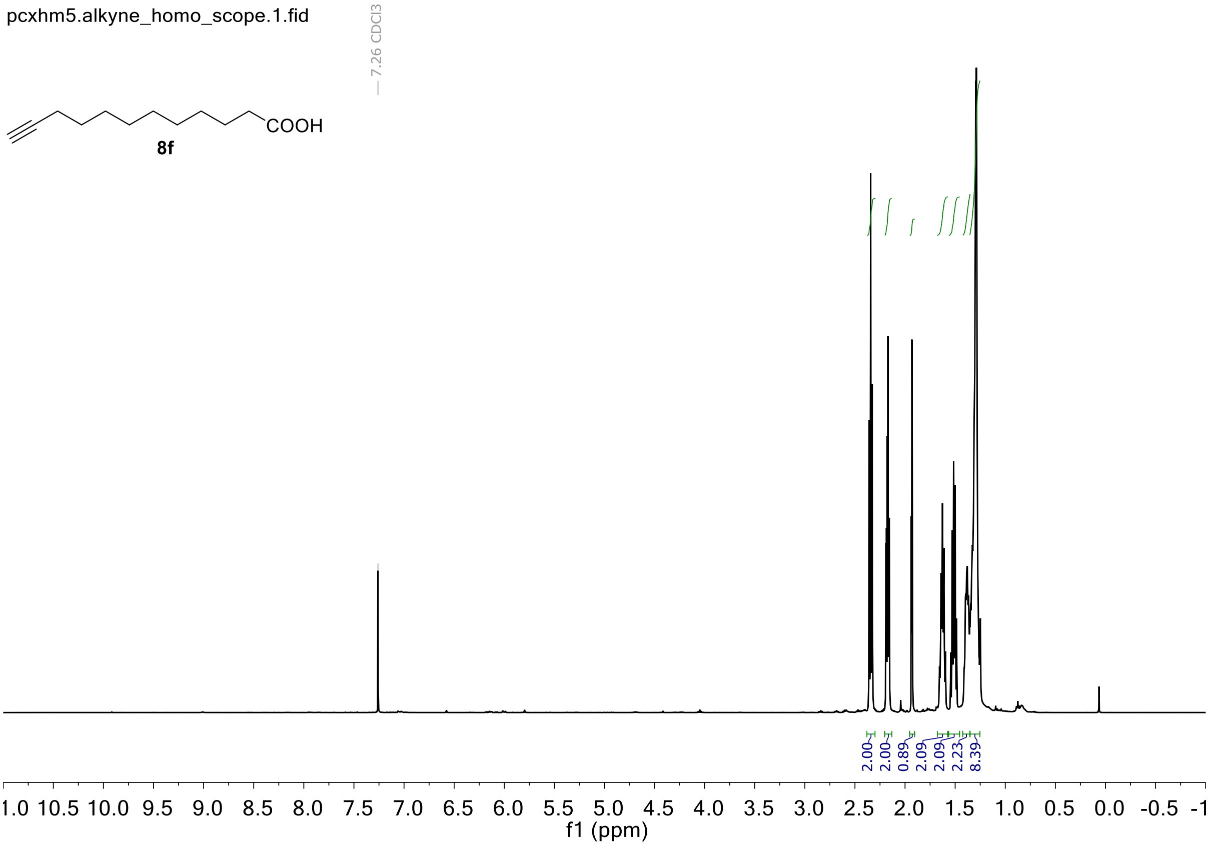

^{13}C-NMR ($CDCl_3$, 126 MHz)

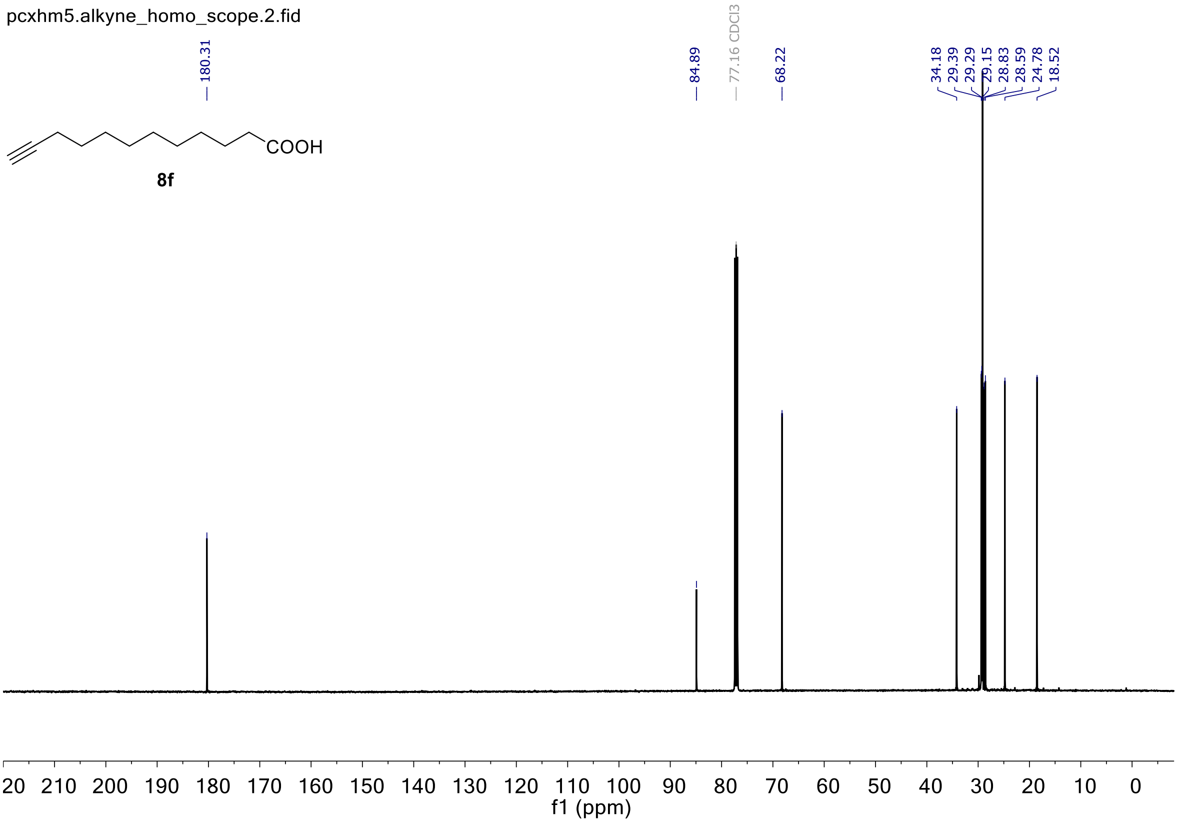

7-(benzylamino)-7-oxoheptanoic acid (8g)

^{1}H-NMR ($CDCl_3$, 400 MHz)

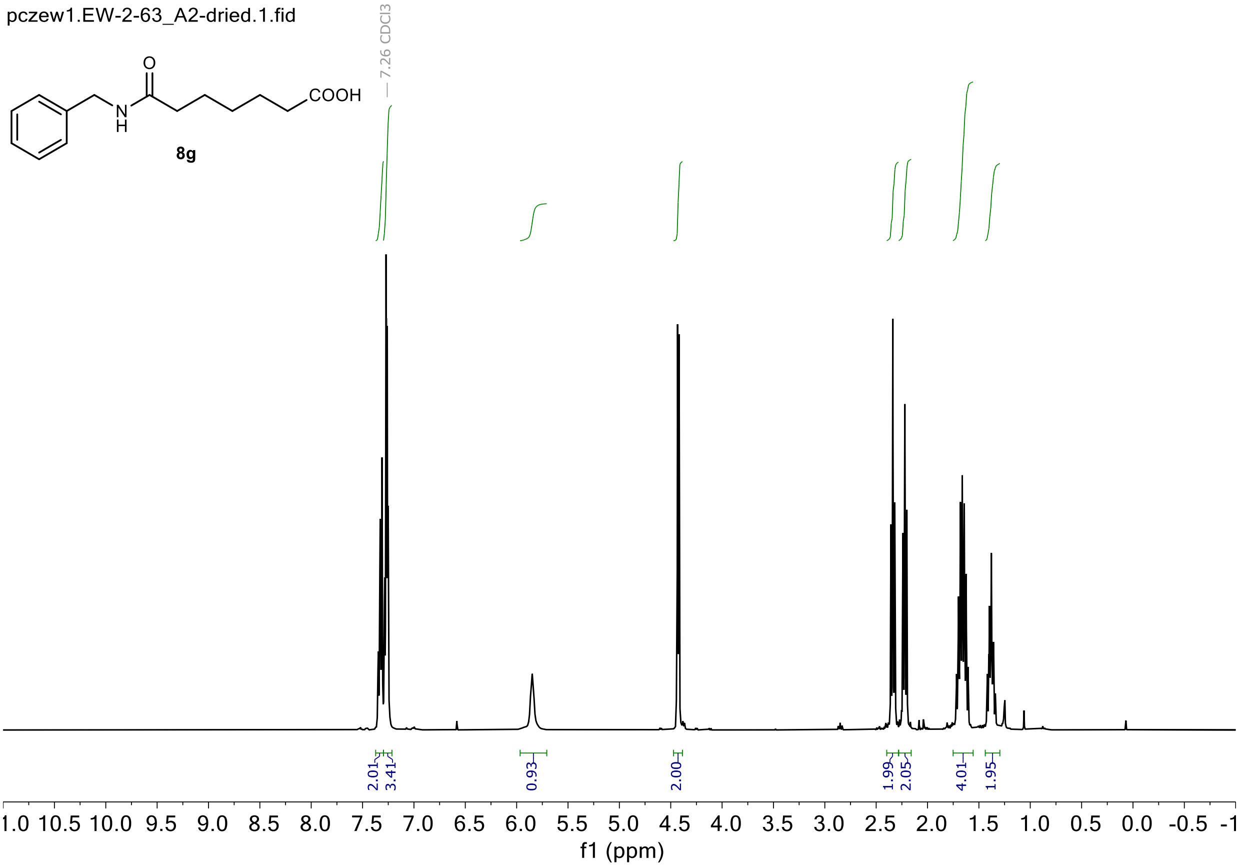

^{13}C-NMR ($CDCl_3$, 101 MHz)

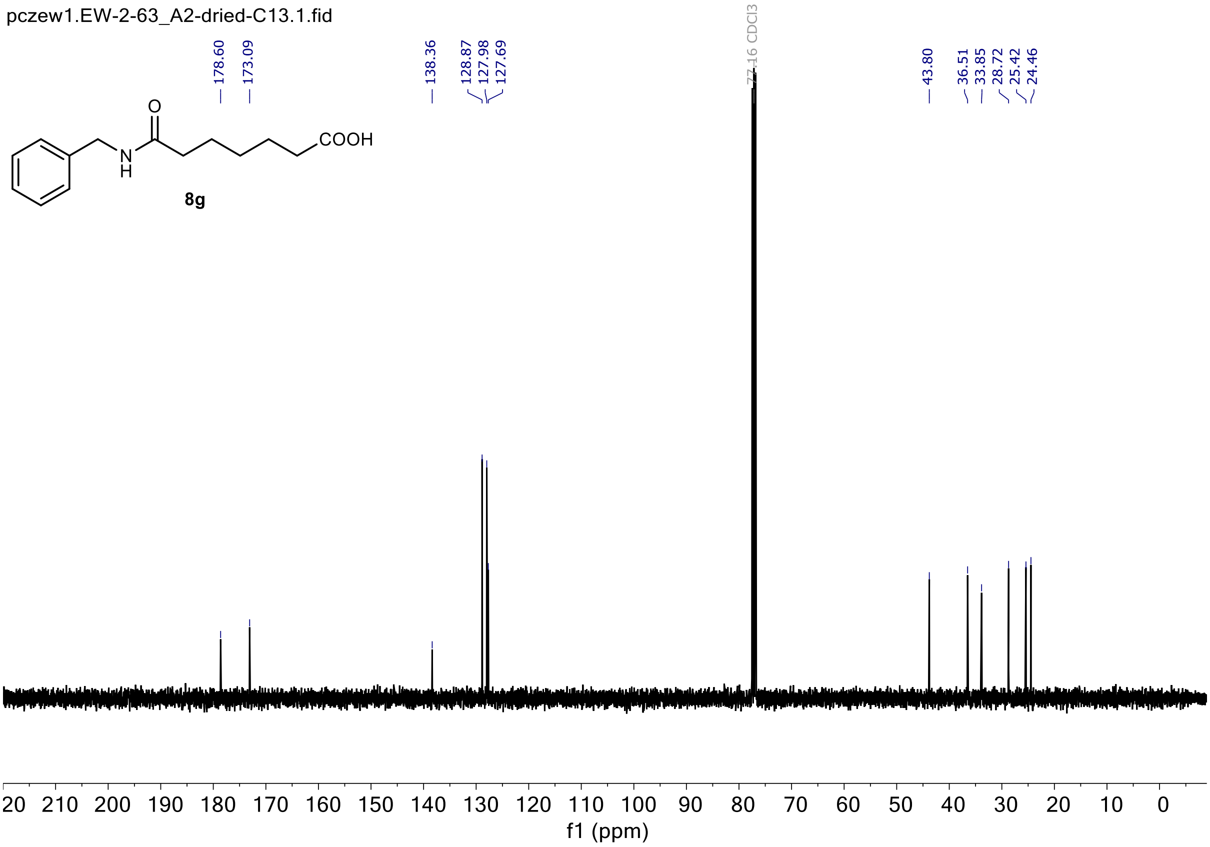

7-(phenylsulfonyl)heptanoic acid (8h)

^{1}H-NMR ($CDCl_3$, 500 MHz)

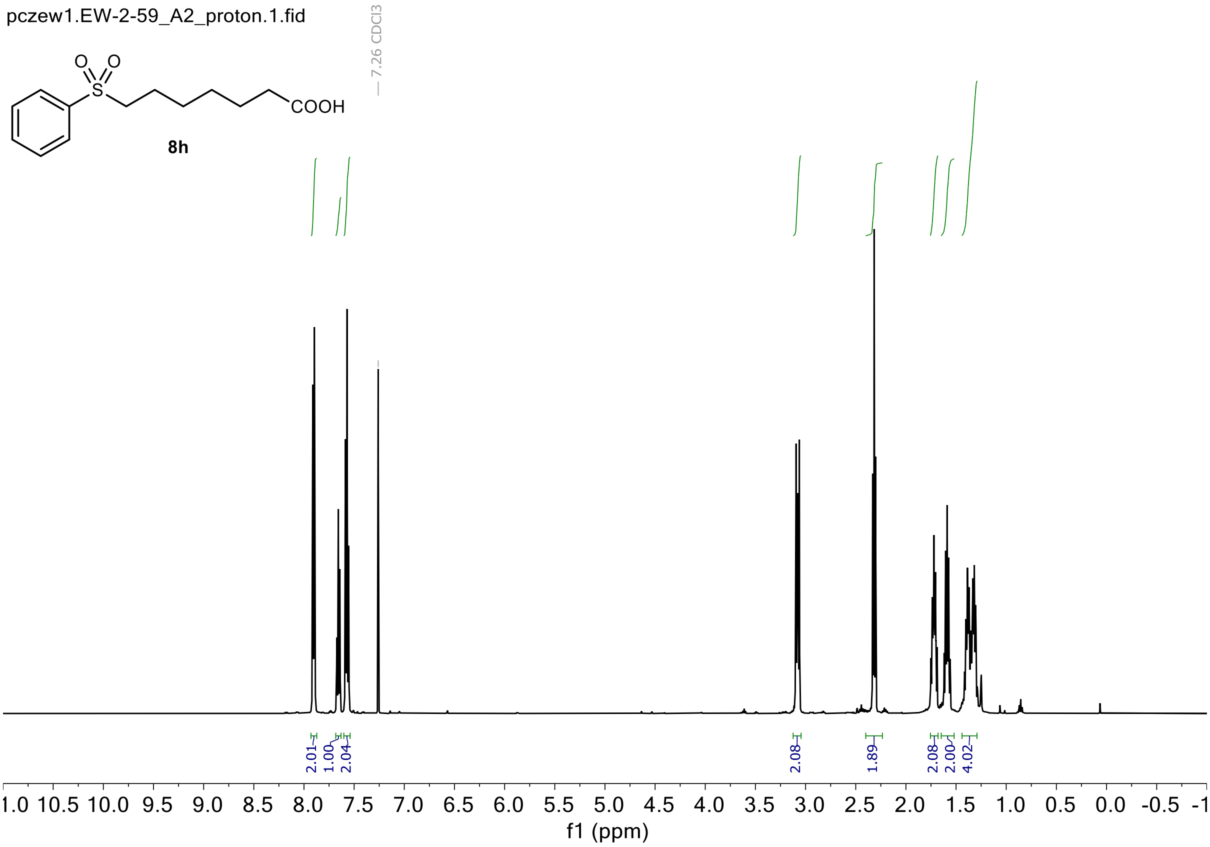

^{13}C-NMR ($CDCl_3$, 126 MHz)

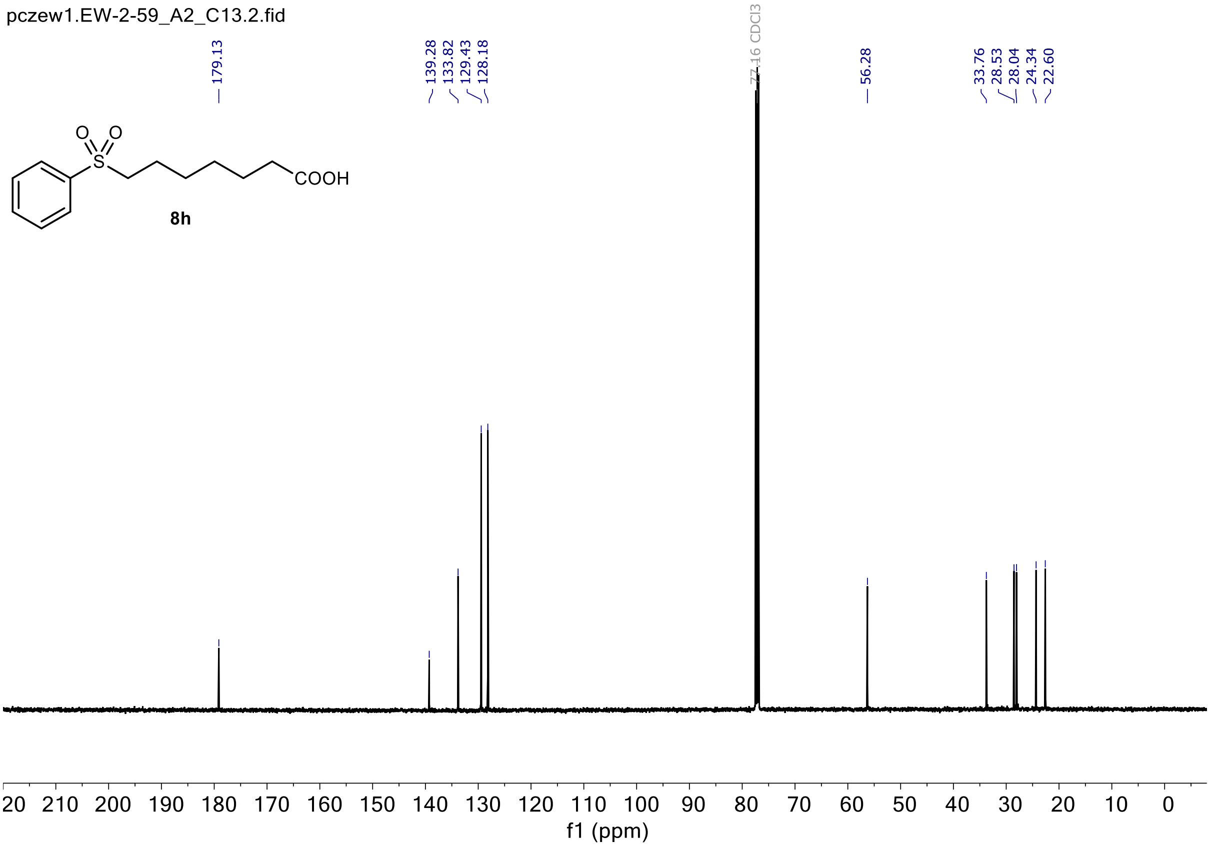

7-(diethoxyphosphoryl)heptanoic acid (8i)

^{1}H-NMR ($CDCl_3$, 500 MHz)

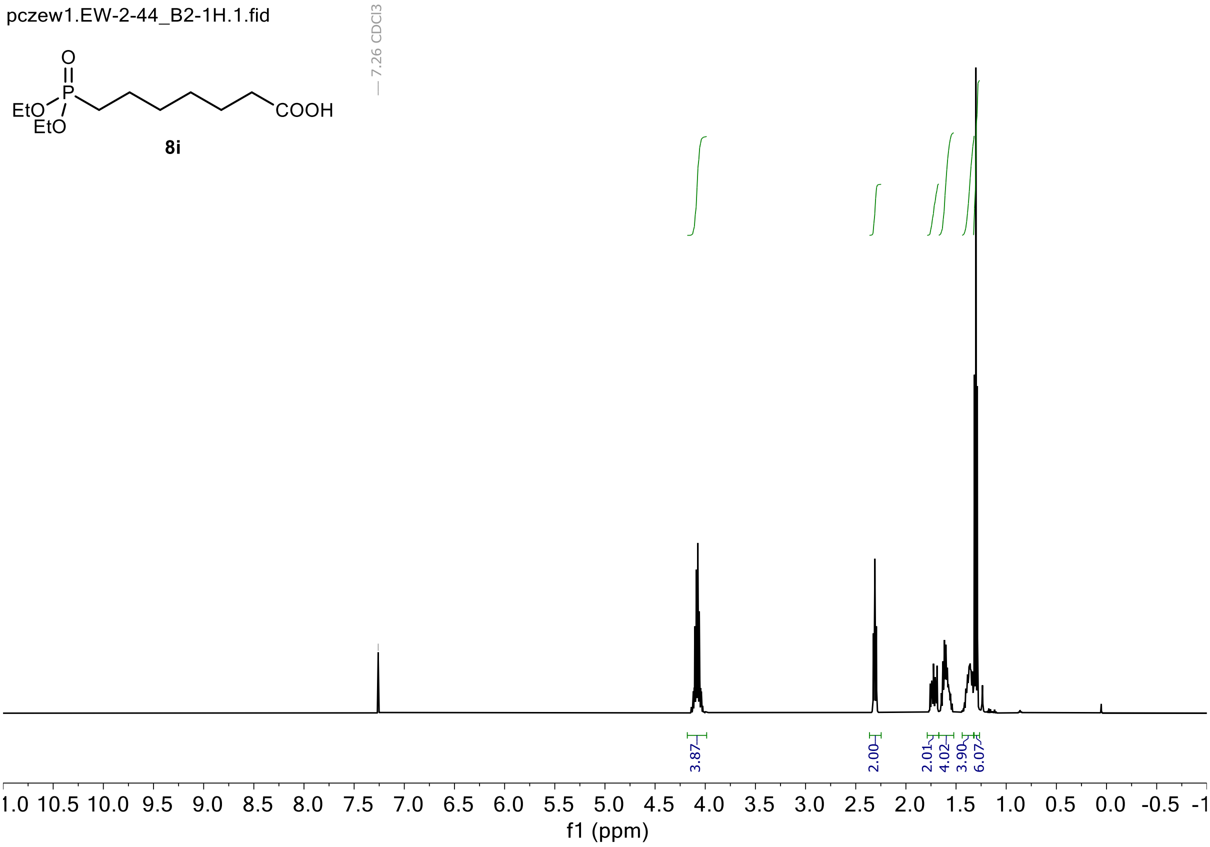

^{13}C-NMR ($CDCl_3$, 126 MHz)

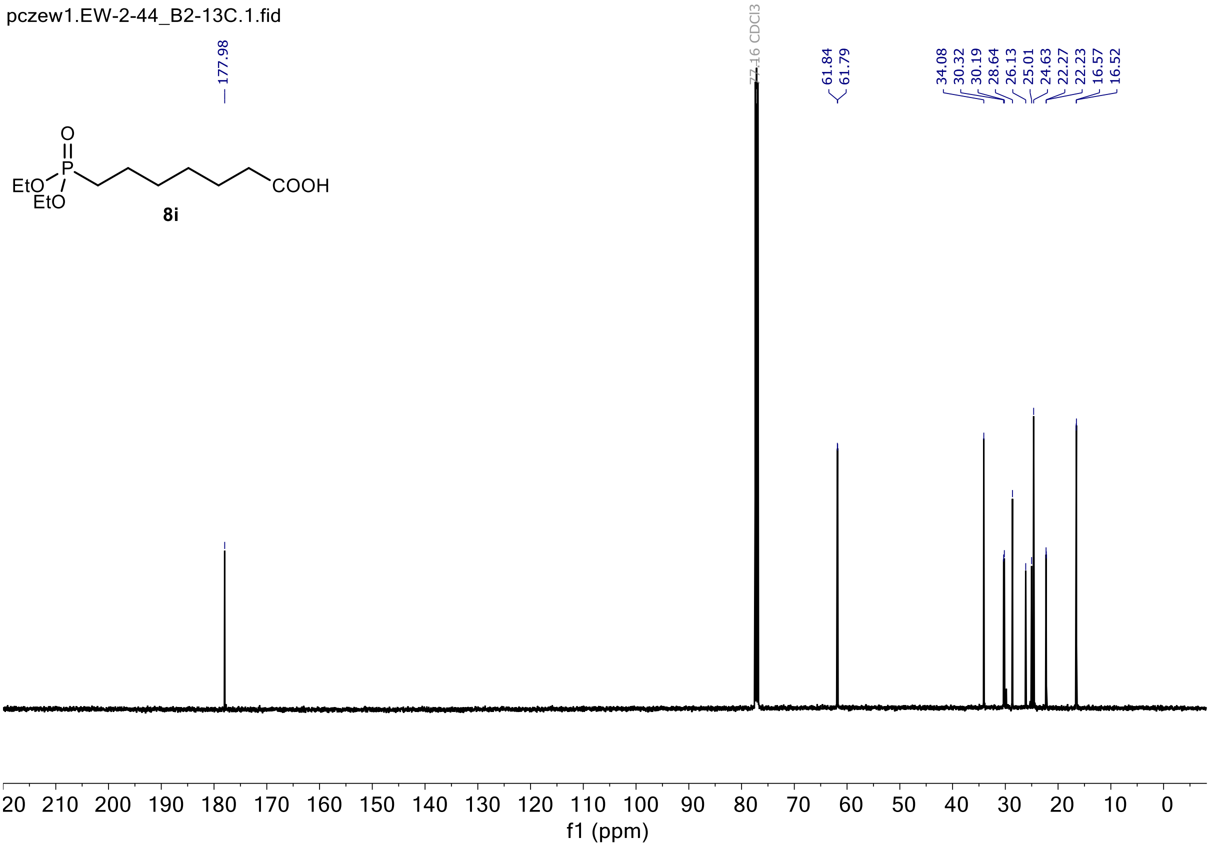

2-cyclohexylacetic acid (8j)

^{1}H-NMR ($CDCl_3$, 500 MHz)

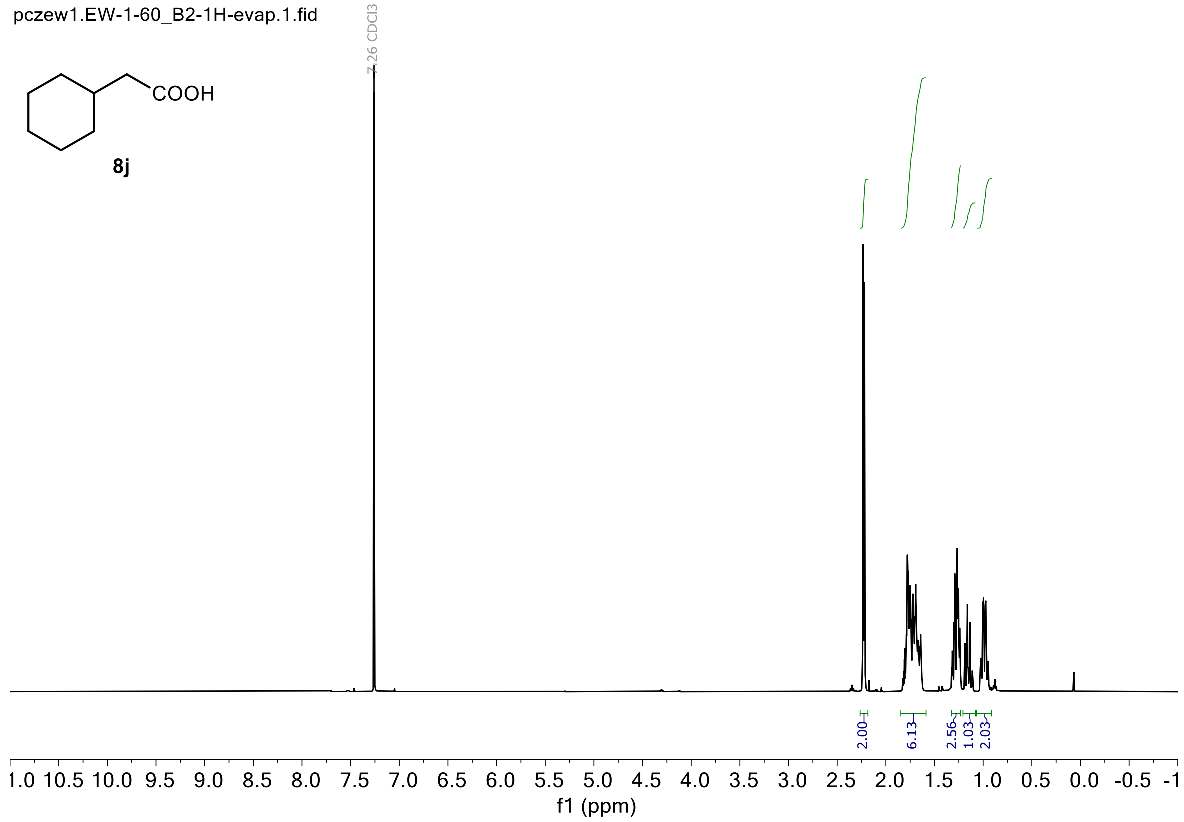

^{13}C-NMR ($CDCl_3$, 126 MHz)

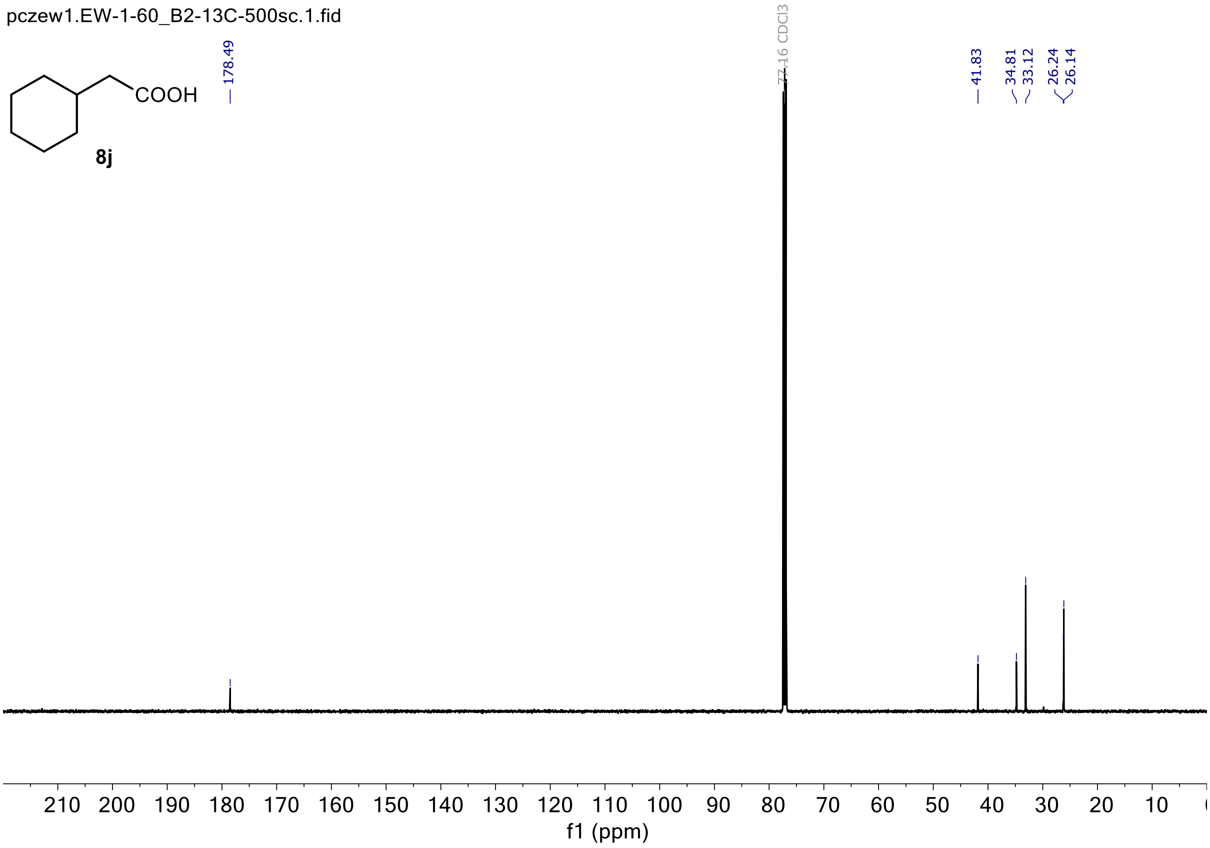

2-(tetrahydro-2H-pyran-4-yl)acetic acid (8a)

^{1}H-NMR ($CDCl_3$, 500 MHz)

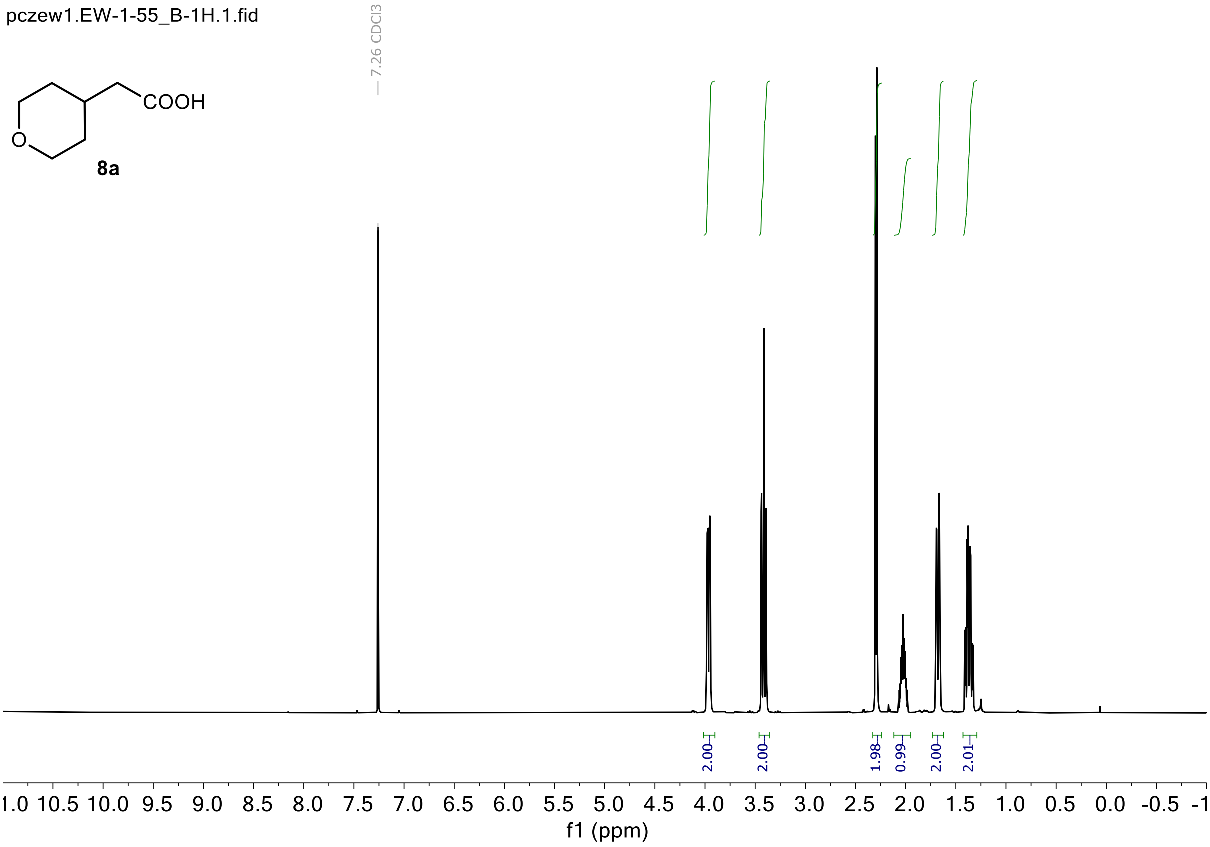

^{13}C-NMR ($CDCl_3$, 126 MHz)

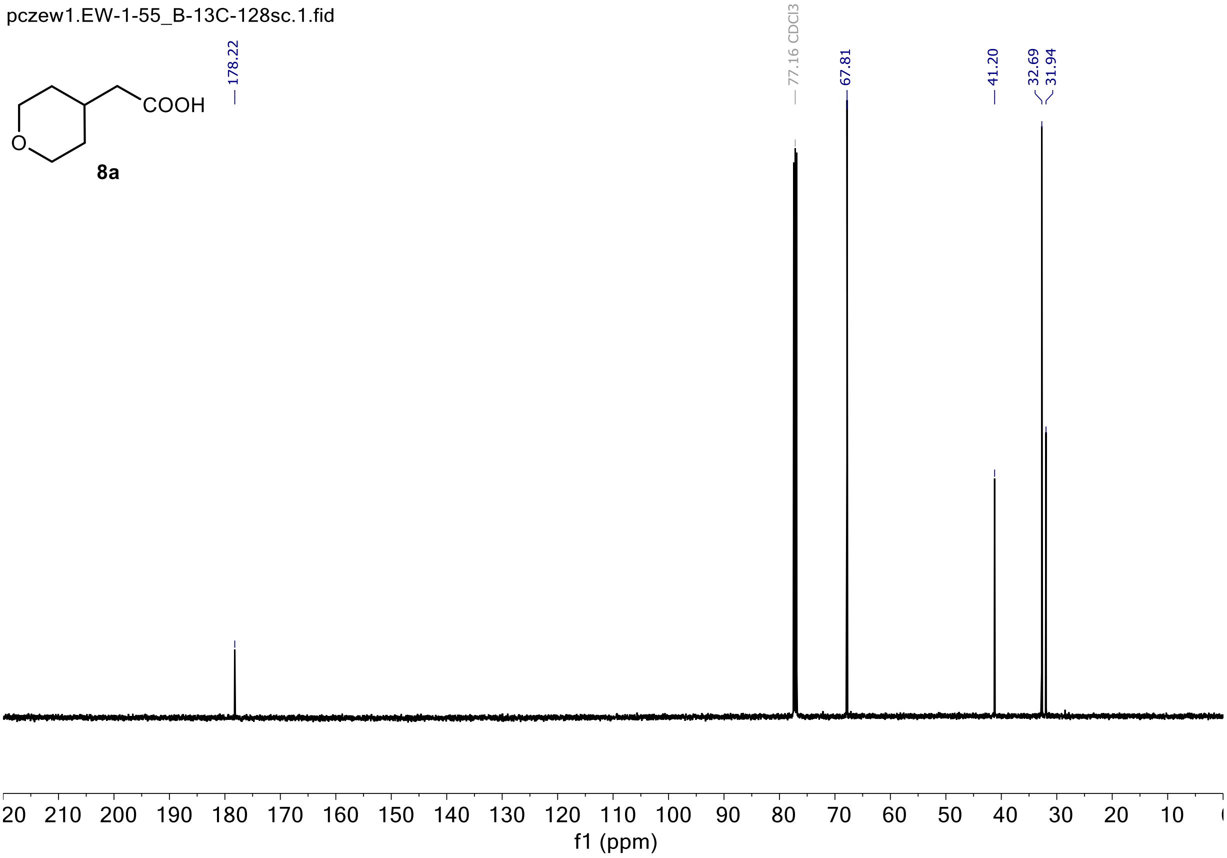

2-(1-(tert-butoxycarbonyl)piperidin-4-yl)acetic acid (8k)

^{1}H-NMR ($CDCl_3$, 500 MHz)

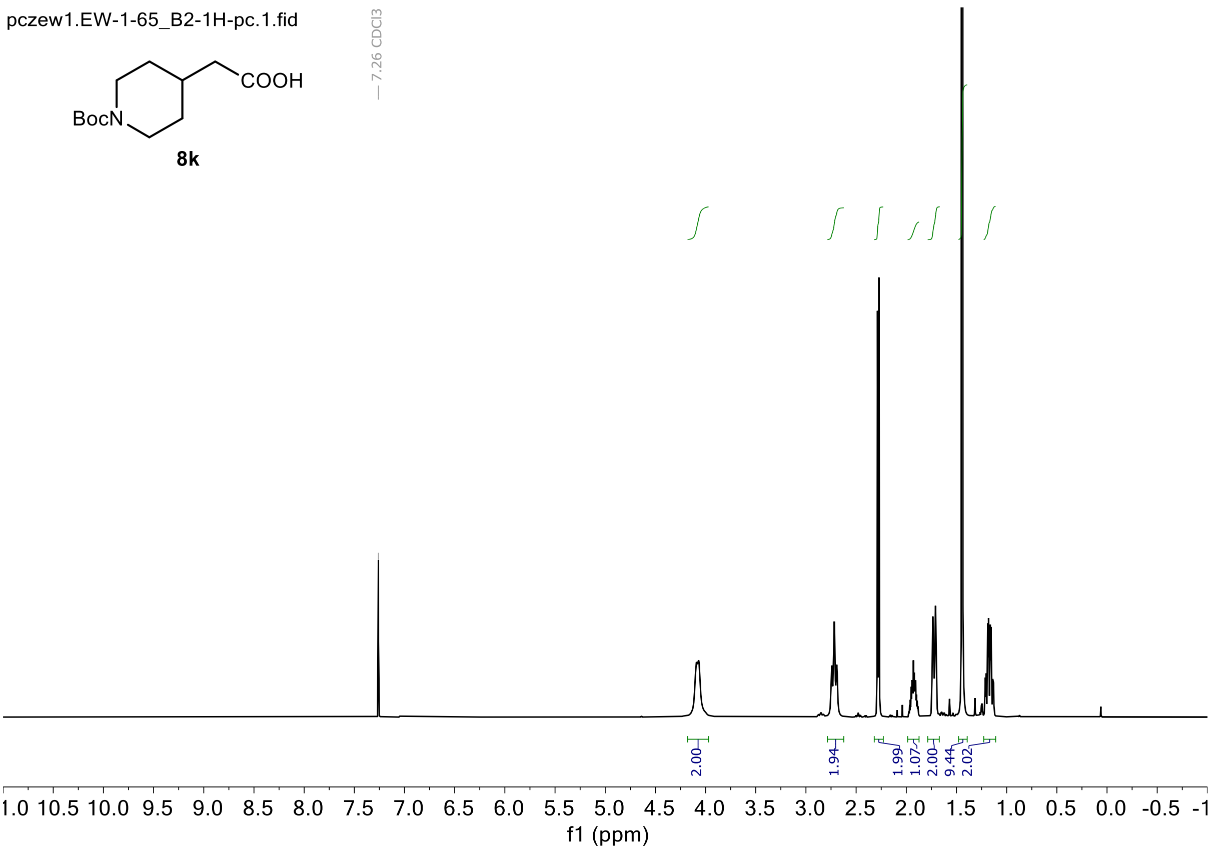

^{13}C-NMR ($CDCl_3$, 126 MHz)

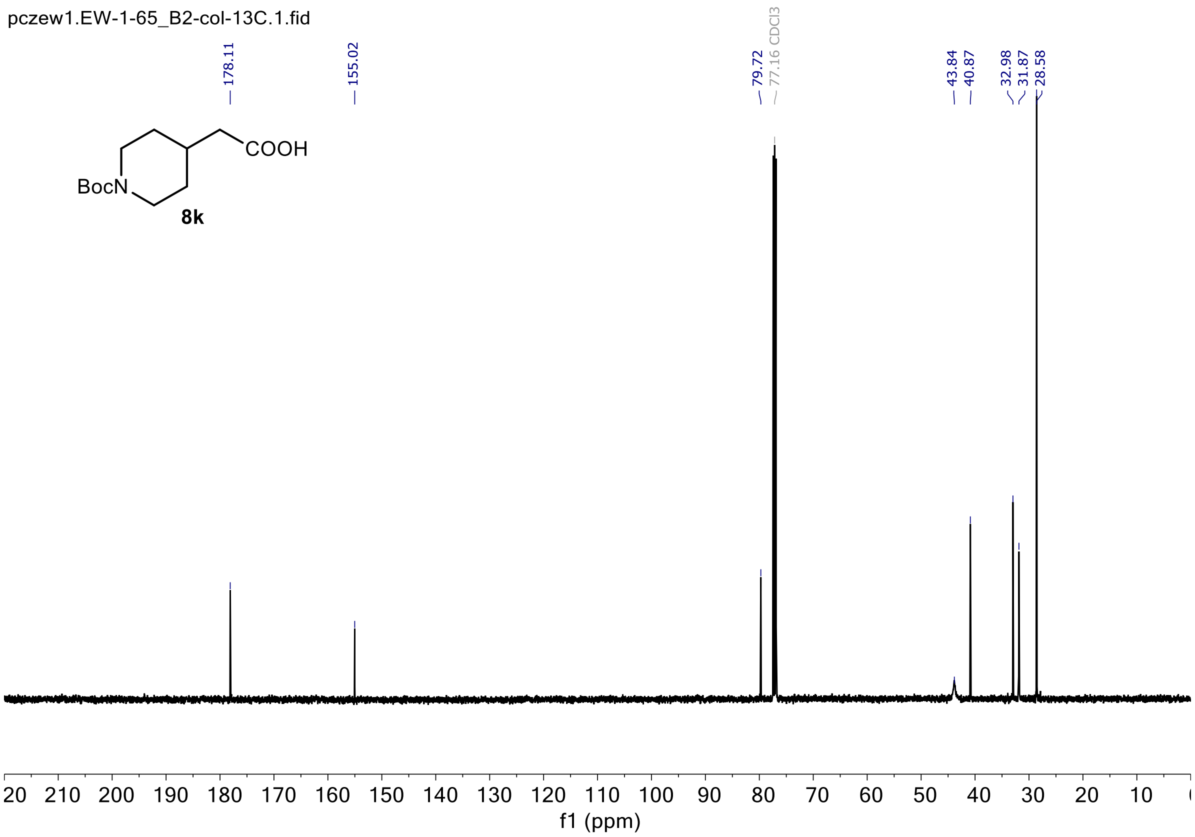

2-(tetrahydrofuran-3-yl)acetic acid (8l)

^{1}H-NMR ($CDCl_3$, 500 MHz)

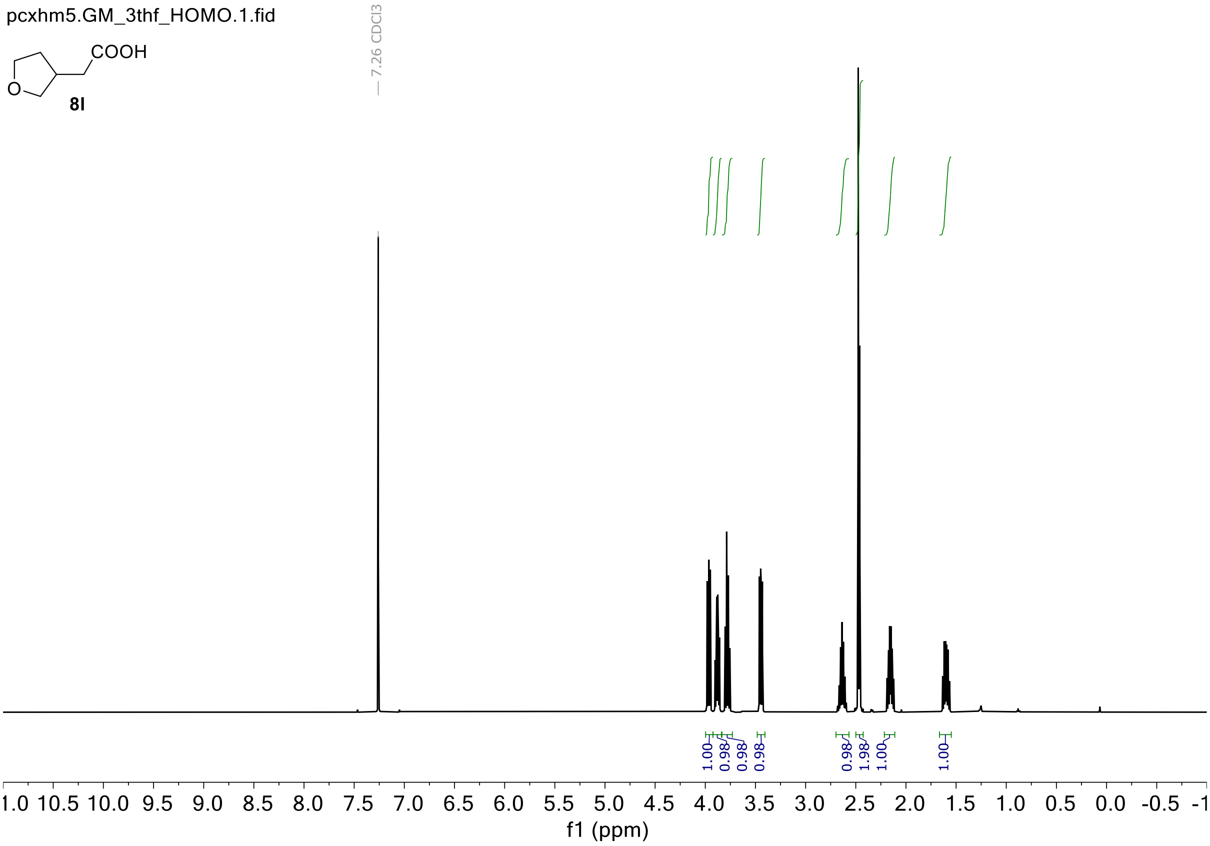

^{13}C-NMR ($CDCl_3$, 126 MHz)

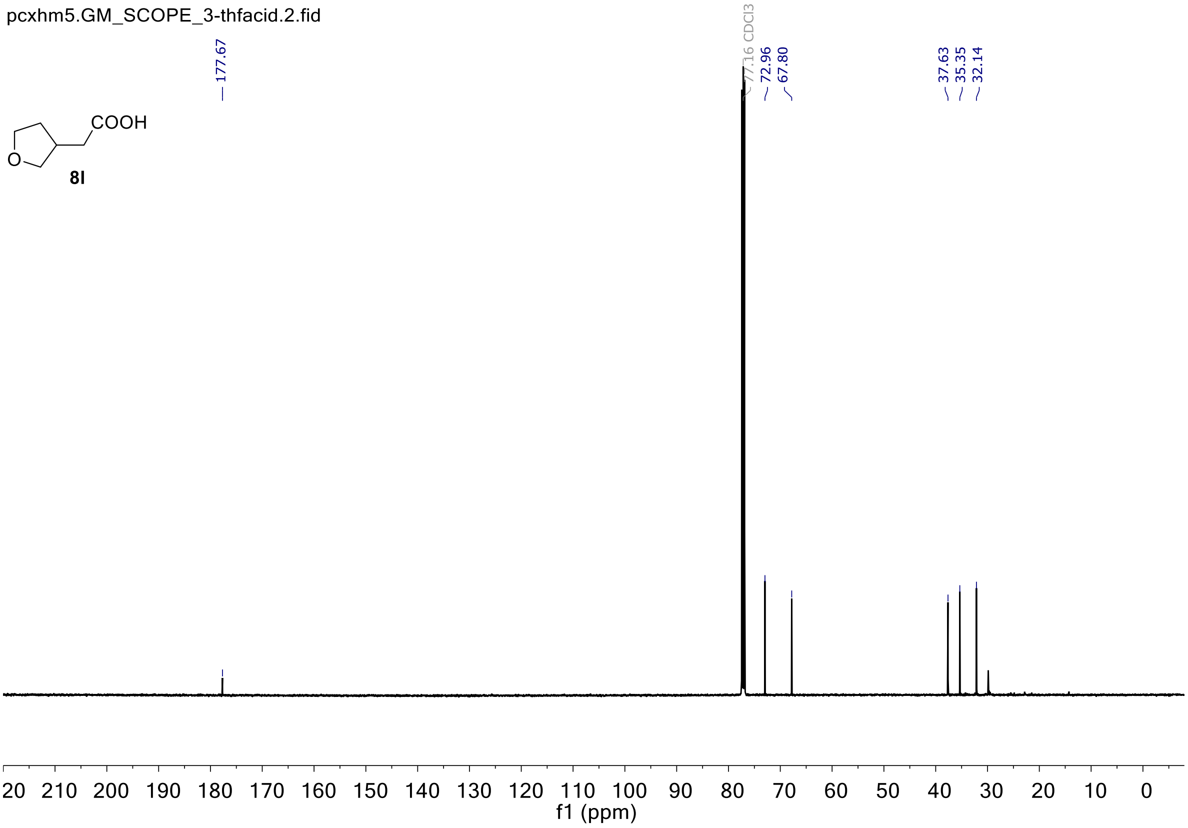

2-(tetrahydrofuran-2-yl)acetic acid (8m)

^{1}H-NMR ($CDCl_3$, 500 MHz)

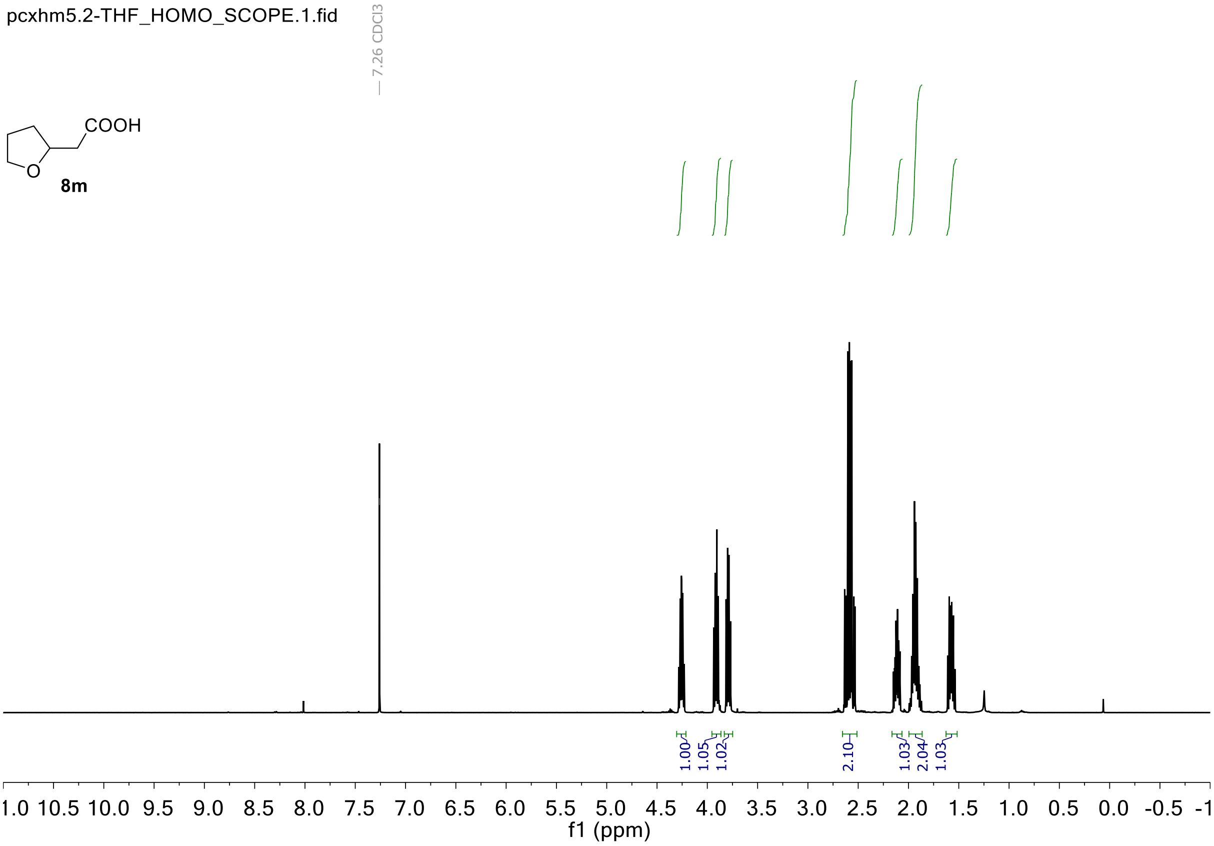

^{13}C-NMR ($CDCl_3$, 126 MHz)

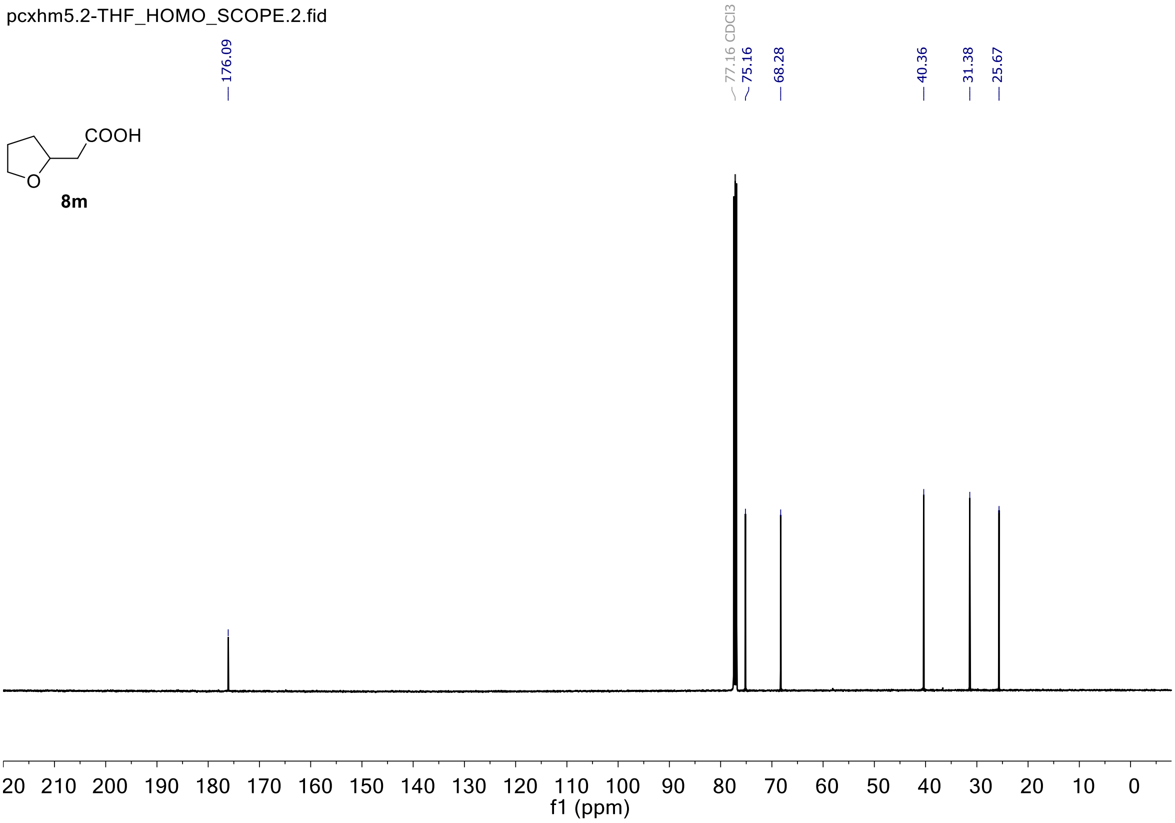

2-(2,3-dihydrobenzofuran-2-yl)acetic acid (8n)

^{1}H-NMR (CDCl$_3$, 500 MHz)

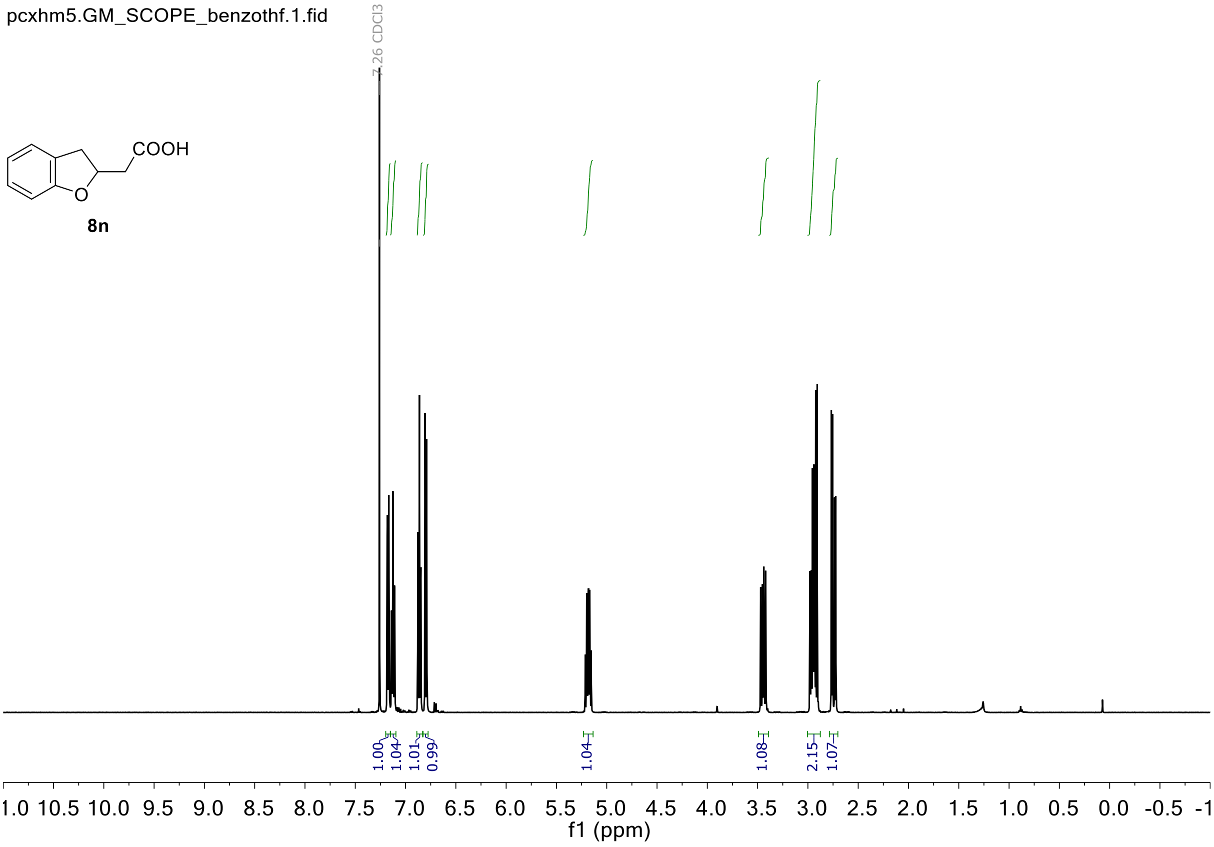

^{13}C-NMR (CDCl$_3$, 126 MHz)

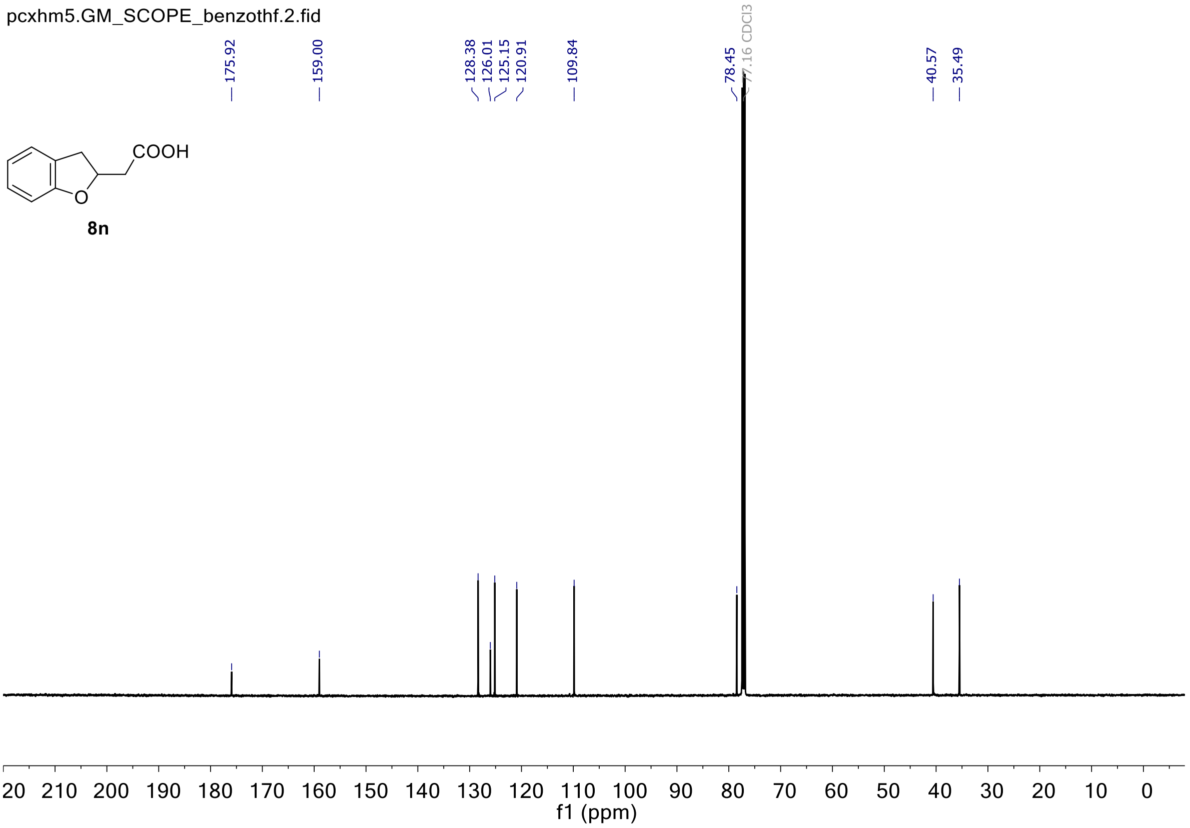

2-(1-(tert-butoxycarbonyl)azetidin-3-yl)acetic acid (8o)

^{1}H-NMR ($CDCl_3$, 500 MHz)

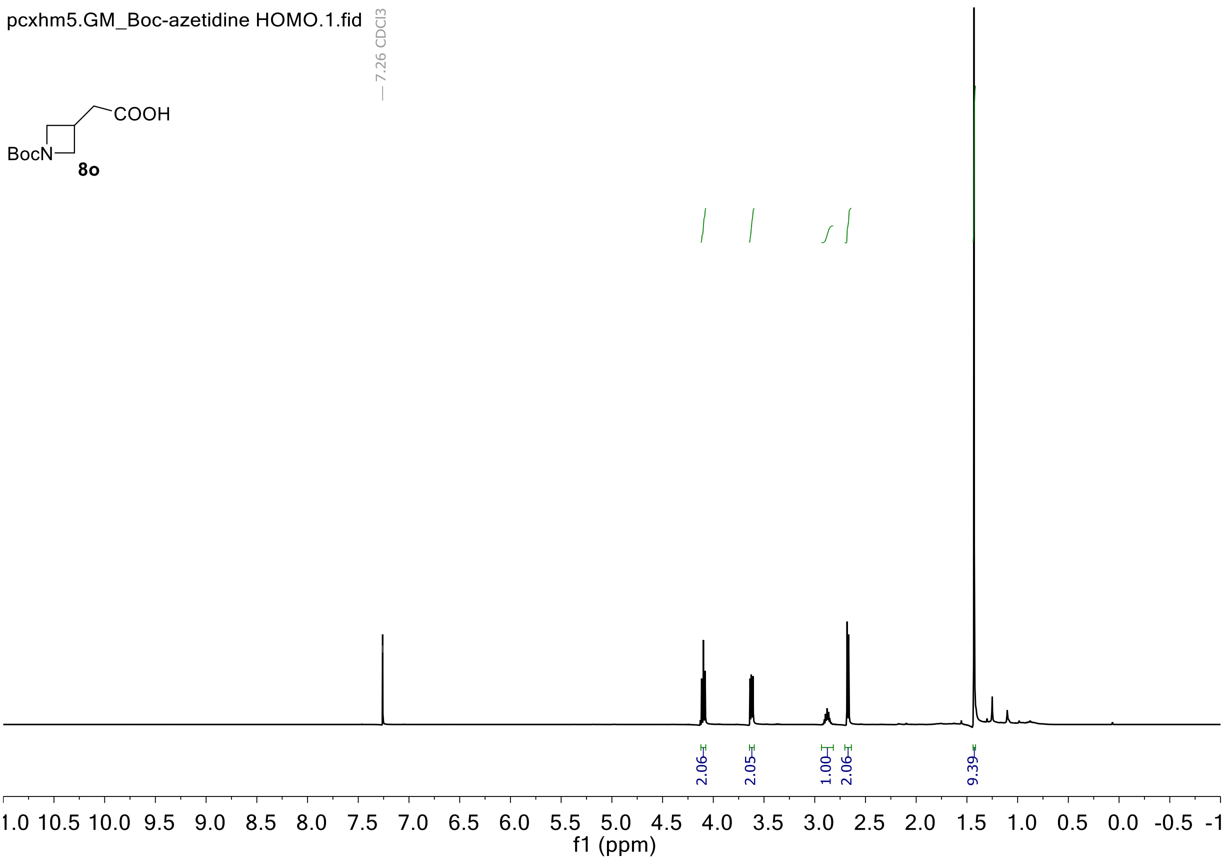

^{13}C-NMR ($CDCl_3$, 126 MHz)

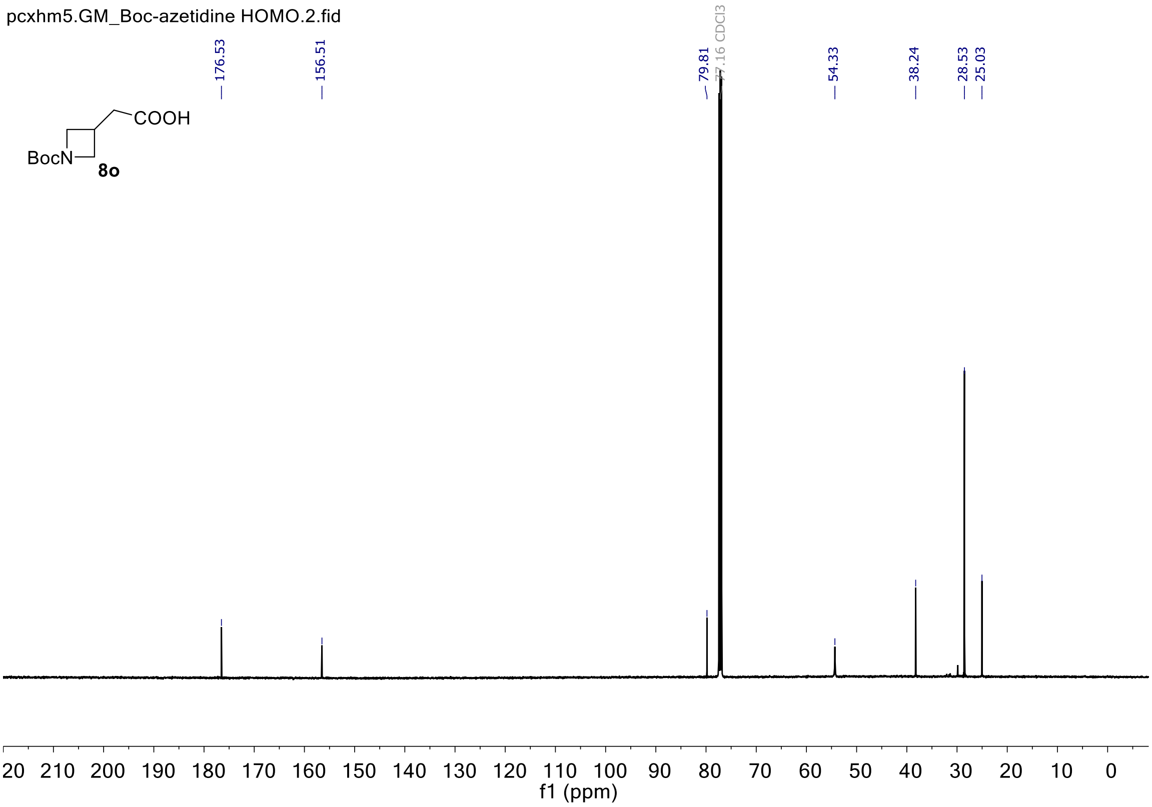

2-((3r,5r,7r)-adamantan-1-yl)acetic acid (8p)

^{1}H-NMR ($CDCl_3$, 500 MHz)

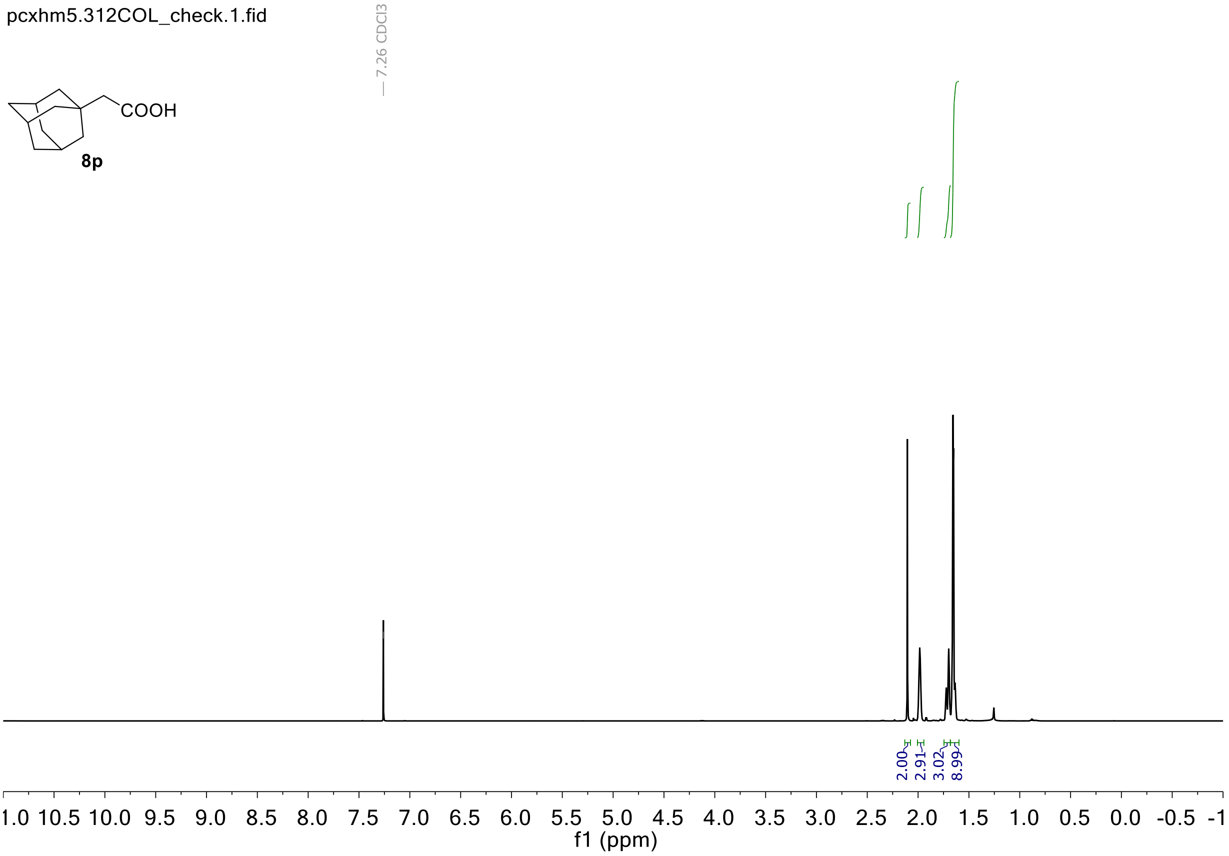

^{13}C-NMR ($CDCl_3$, 126 MHz)

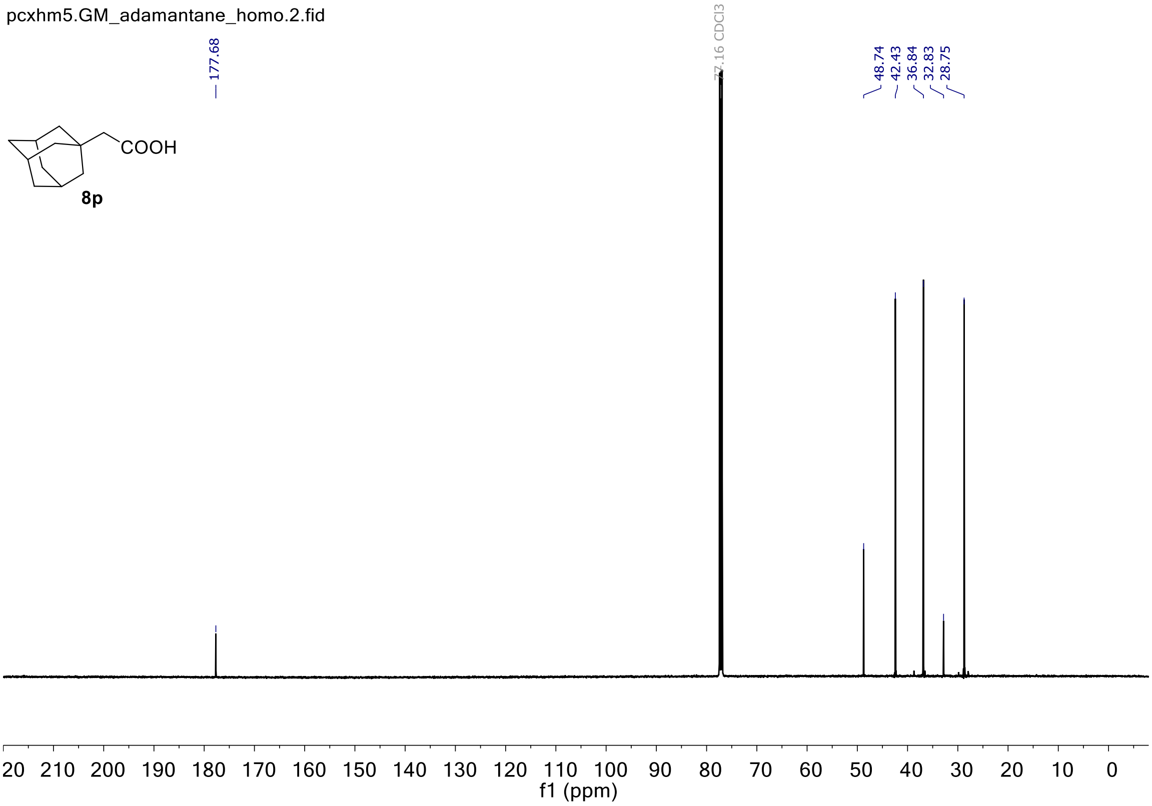

2-(1-phenylcyclopropyl)acetic acid (8q)

^{1}H-NMR (CDCl$_3$, 500 MHz)

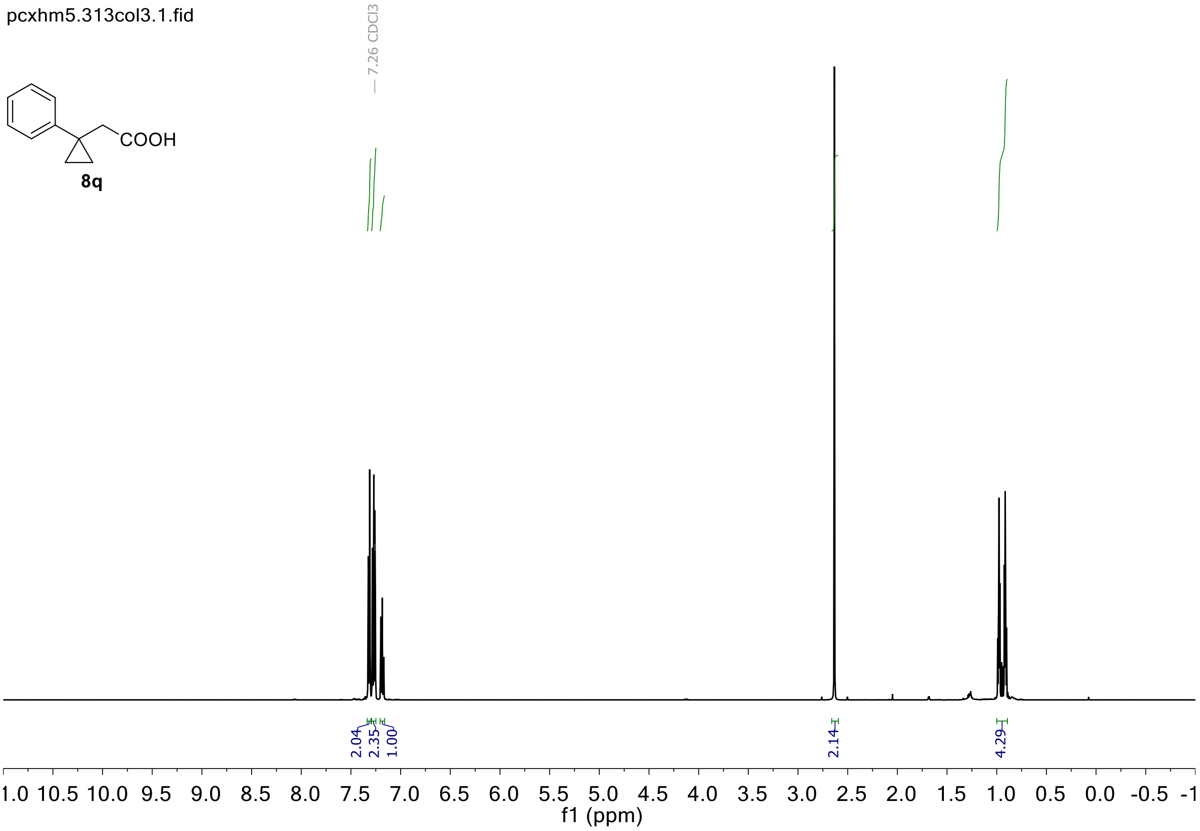

^{13}C-NMR (CDCl$_3$, 126 MHz)

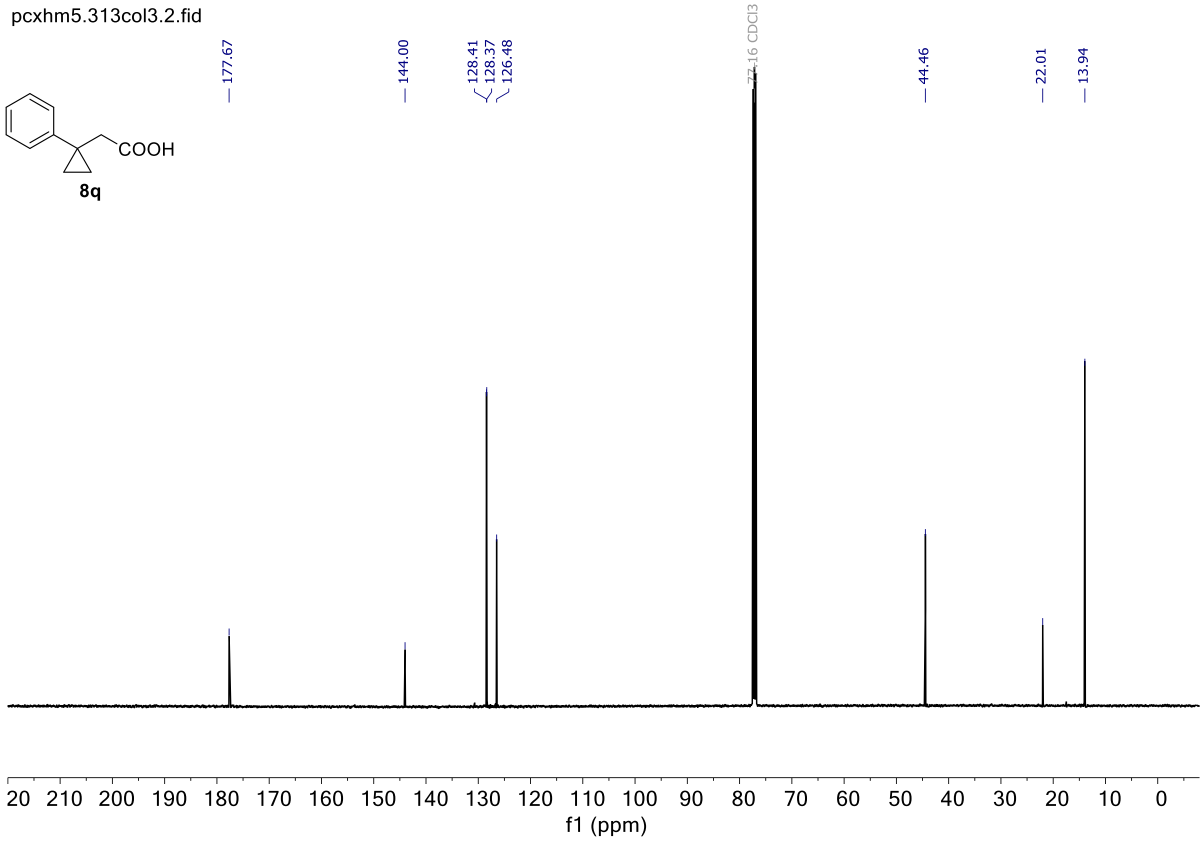

2-(3-(methoxycarbonyl)bicyclo[1.1.1]pentan-1-yl)acetic acid (8r)

^{1}H-NMR ($CDCl_3$, 500 MHz)

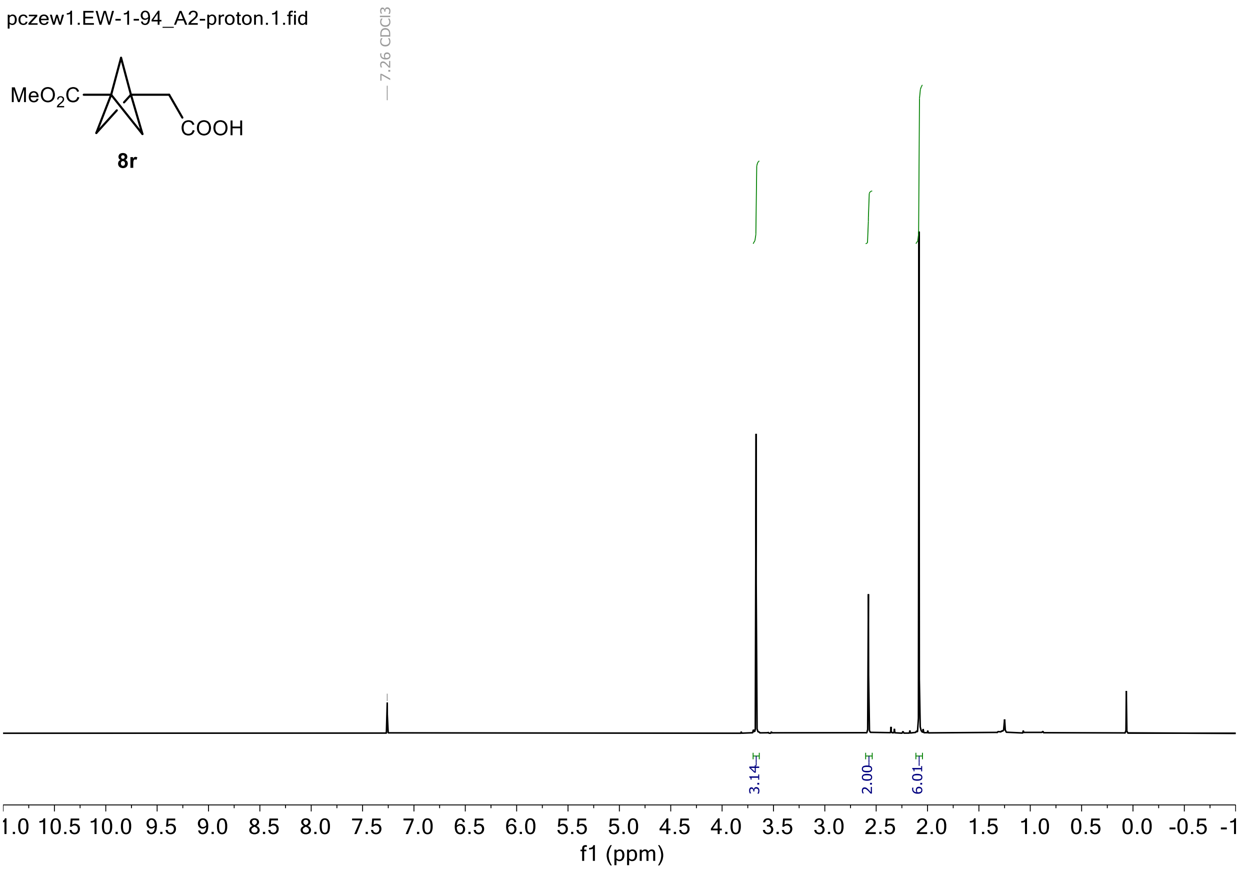

^{13}C-NMR ($CDCl_3$, 126 MHz)

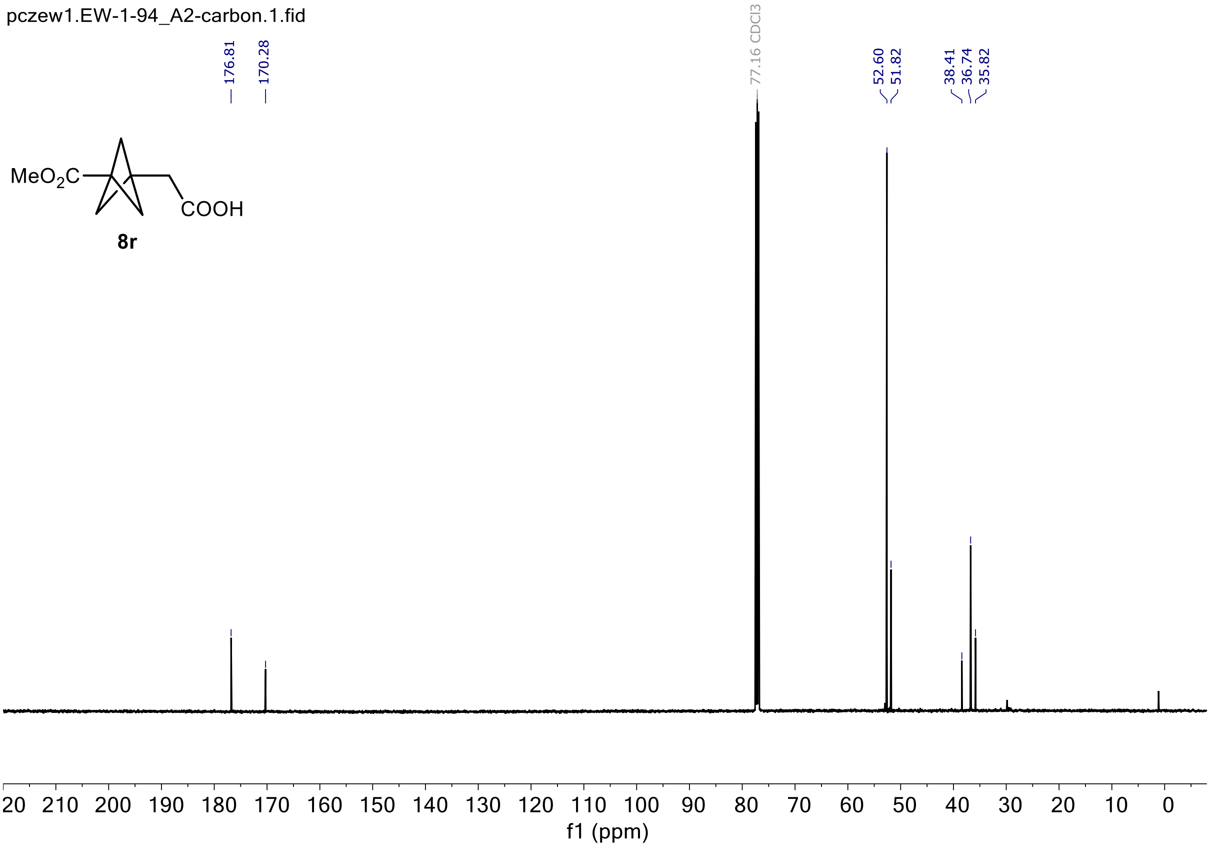

2-(4-(methoxycarbonyl)bicyclo[2.2.2]octan-1-yl)acetic acid (8s)

^{1}H-NMR ($CDCl_3$, 500 MHz)

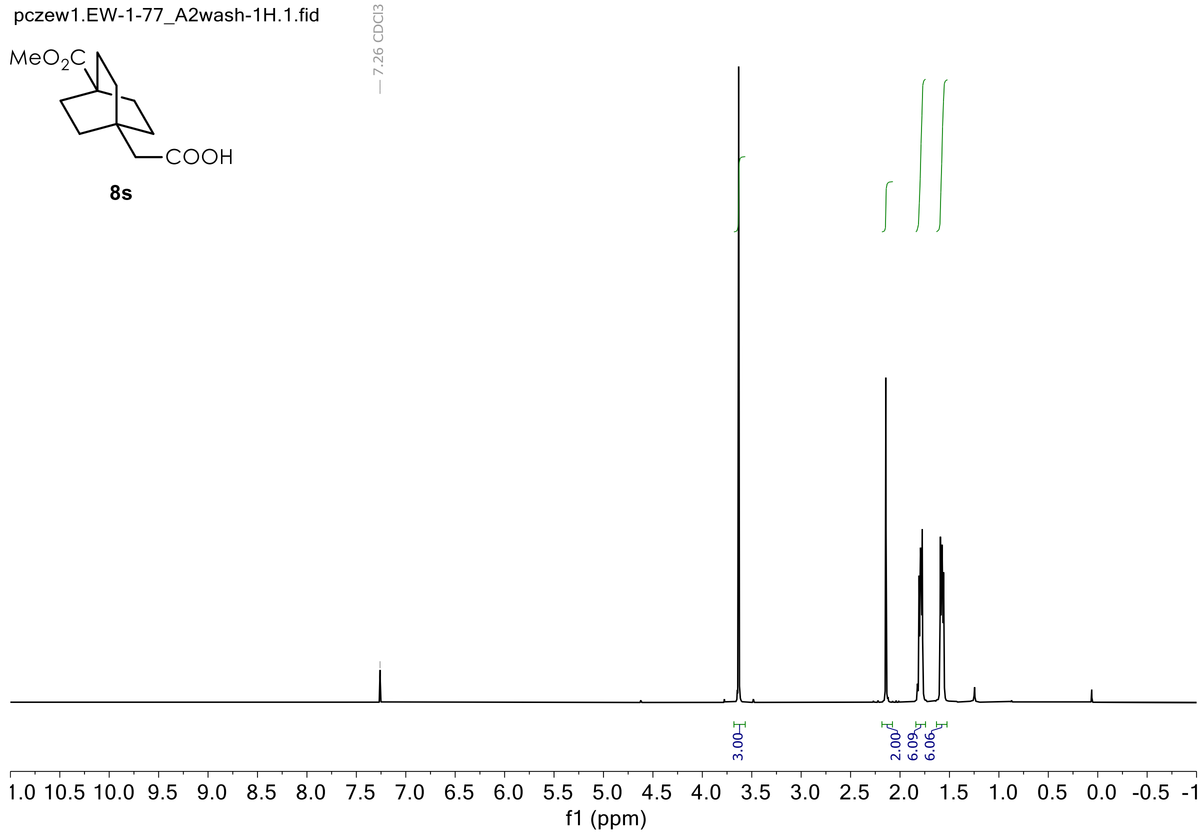

^{13}C-NMR ($CDCl_3$, 126 MHz)

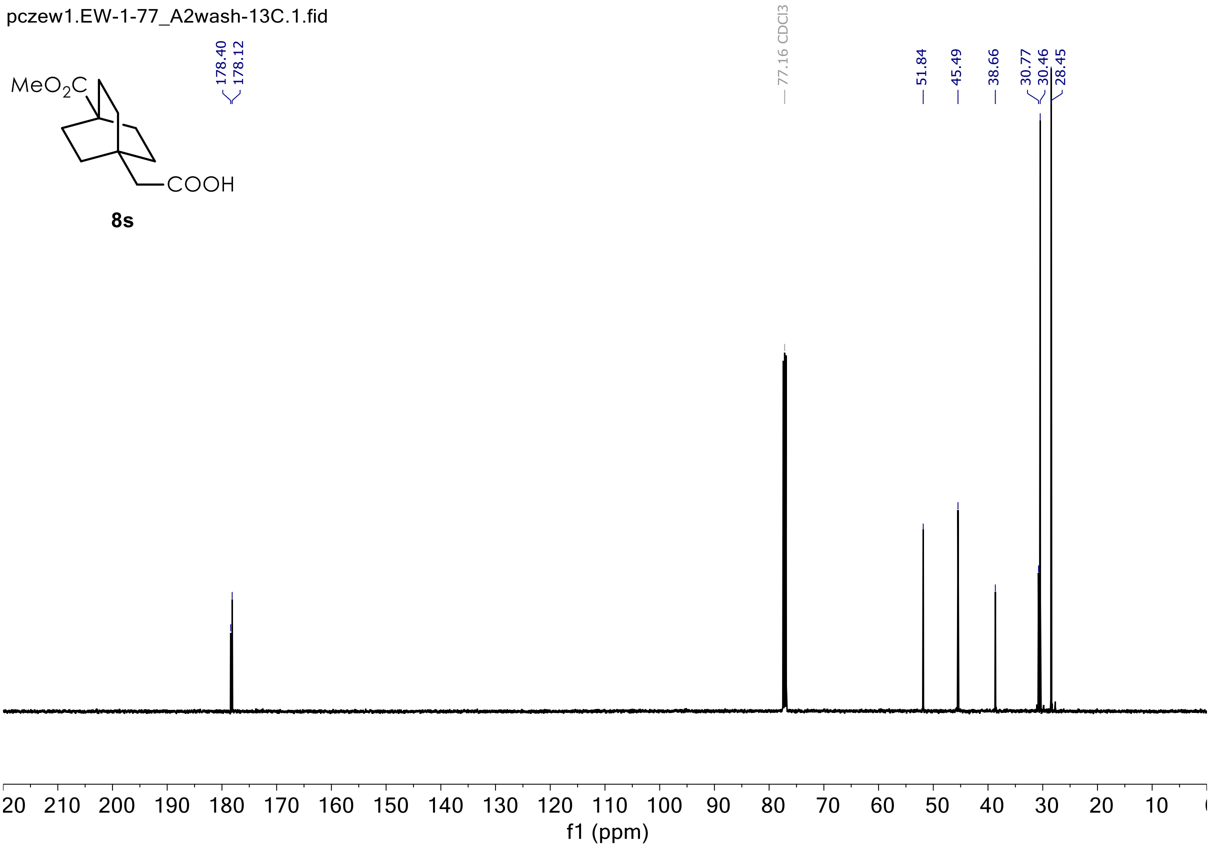

2-((1R,4R)-7,7-dimethyl-2-oxobicyclo[2.2.1]heptan-1-yl)acetic acid (8t)

^{1}H-NMR ($CDCl_3$, 500 MHz)

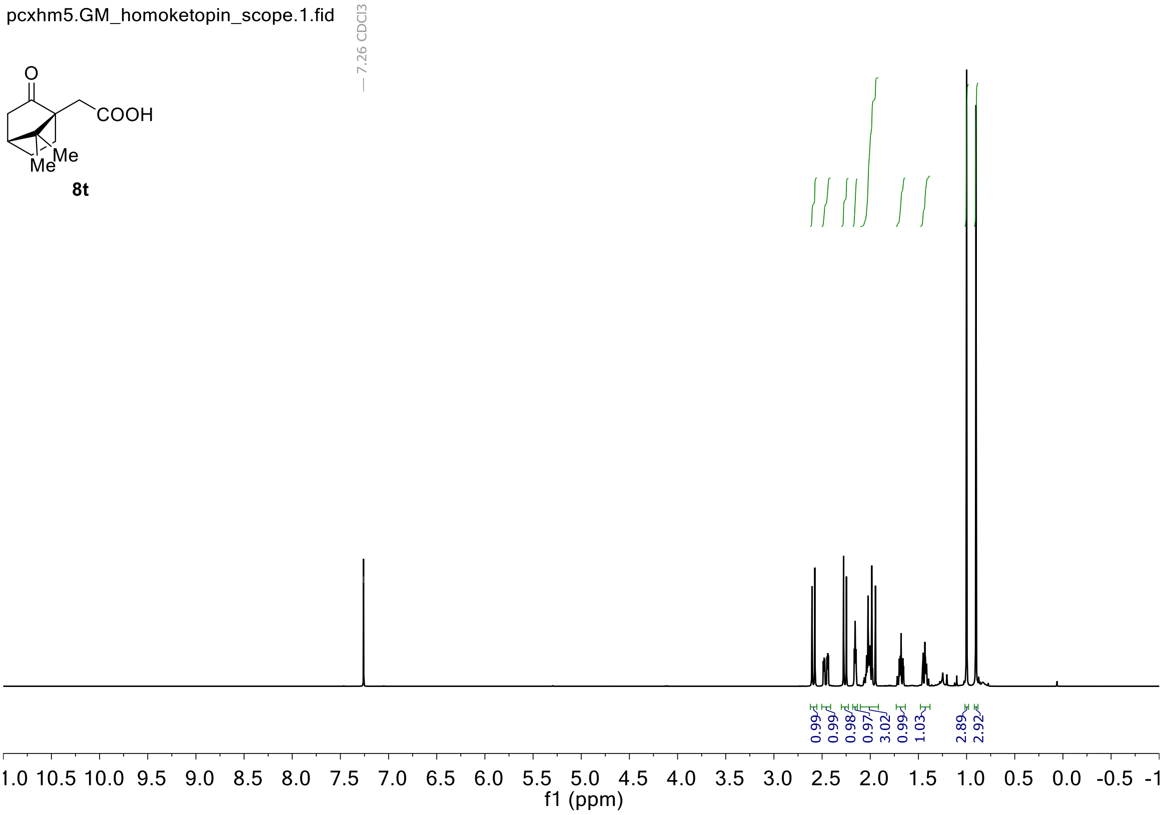

^{13}C-NMR ($CDCl_3$, 126 MHz)

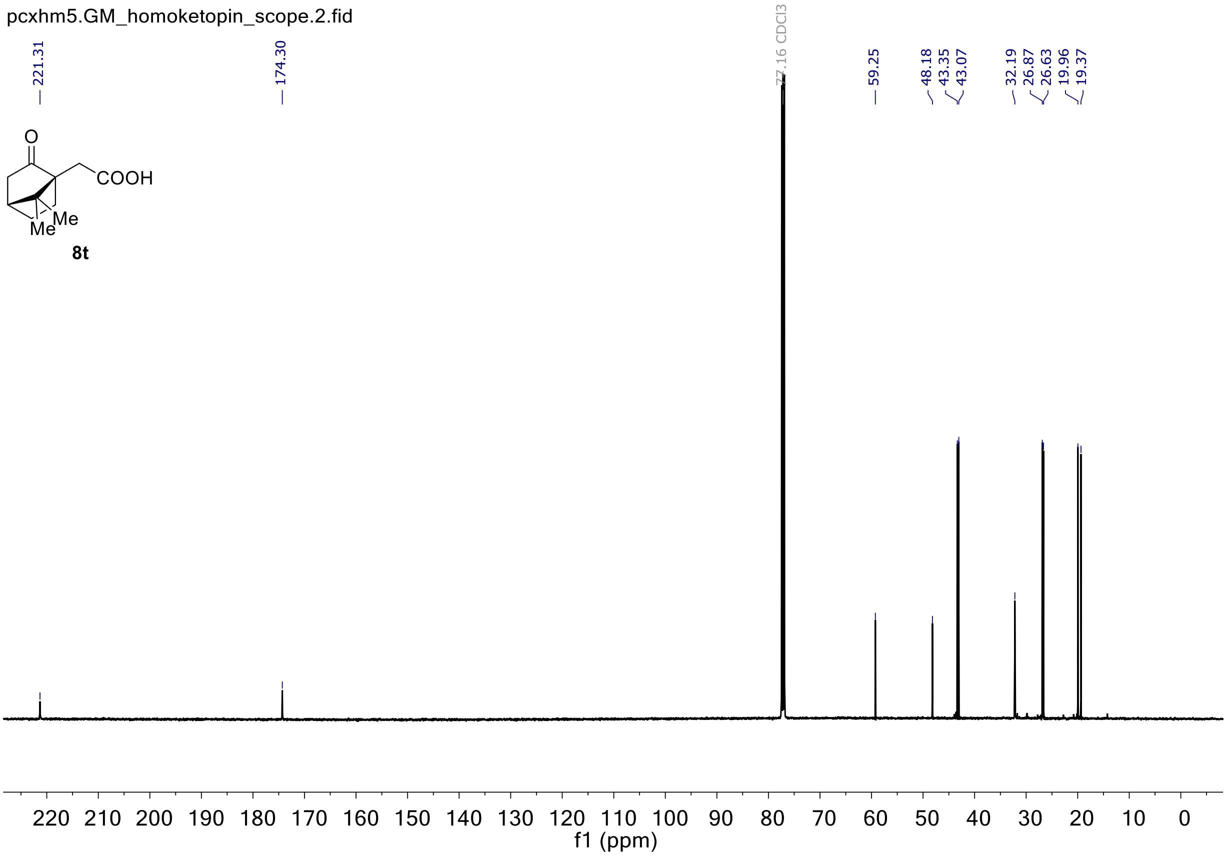

6-(2,5-dimethylphenoxy)-3,3-dimethylhexanoic acid (8u)

^{1}H-NMR (CDCl$_3$, 500 MHz)

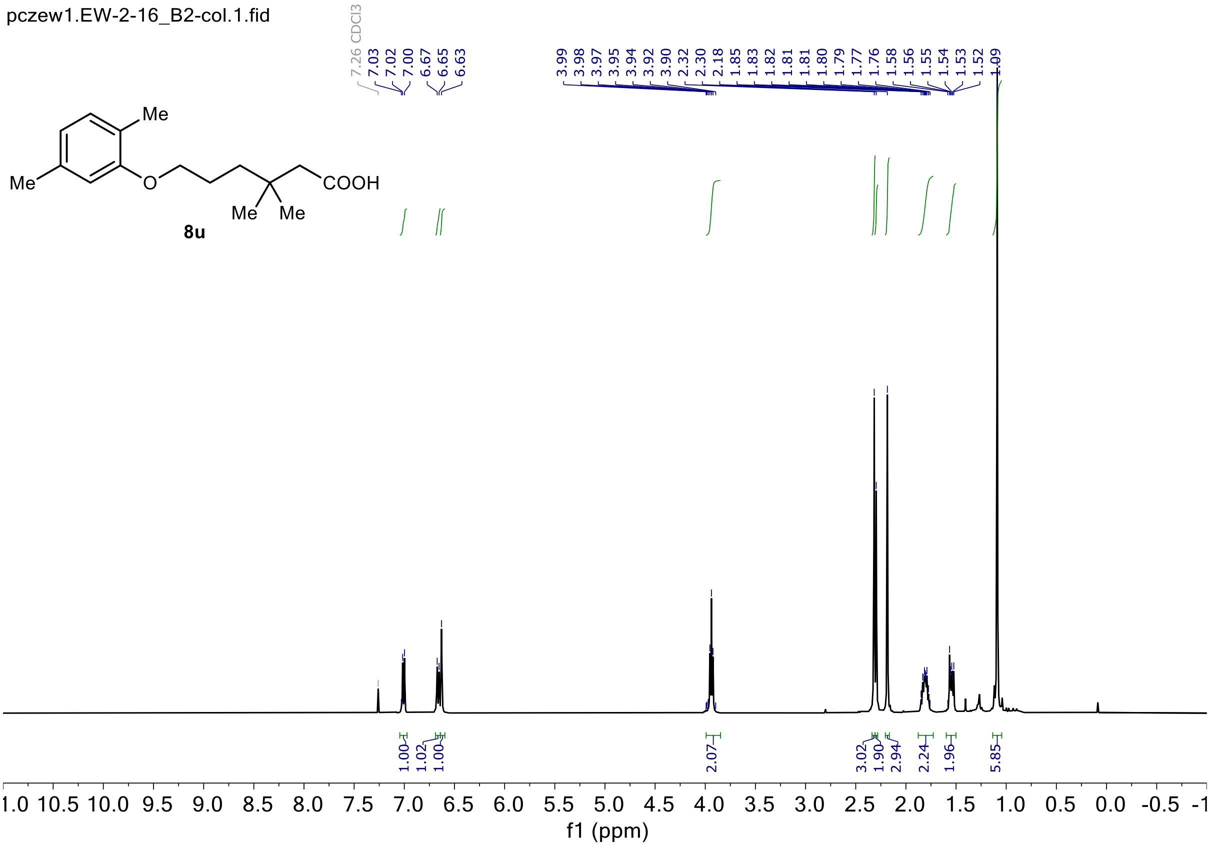

^{13}C-NMR (CDCl$_3$, 126 MHz)

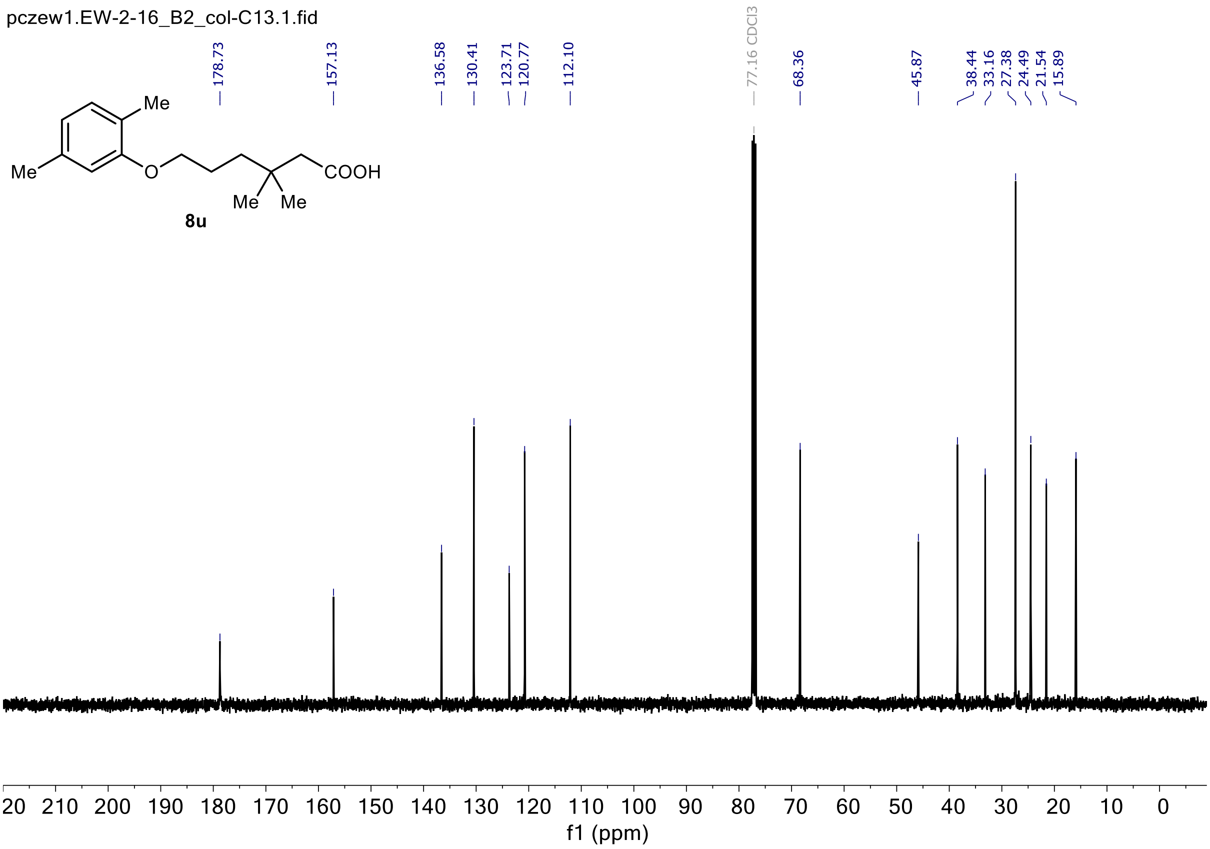

(5R)-5-((3R,8R,9S,10S,13R,14S,17R)-3-hydroxy-10,13-dimethylhexadecahydro-1H-cyclopenta[a]phenanthren-17-yl)hexanoic acid (8v)

^{1}H-NMR ($CDCl_3$, 400 MHz)

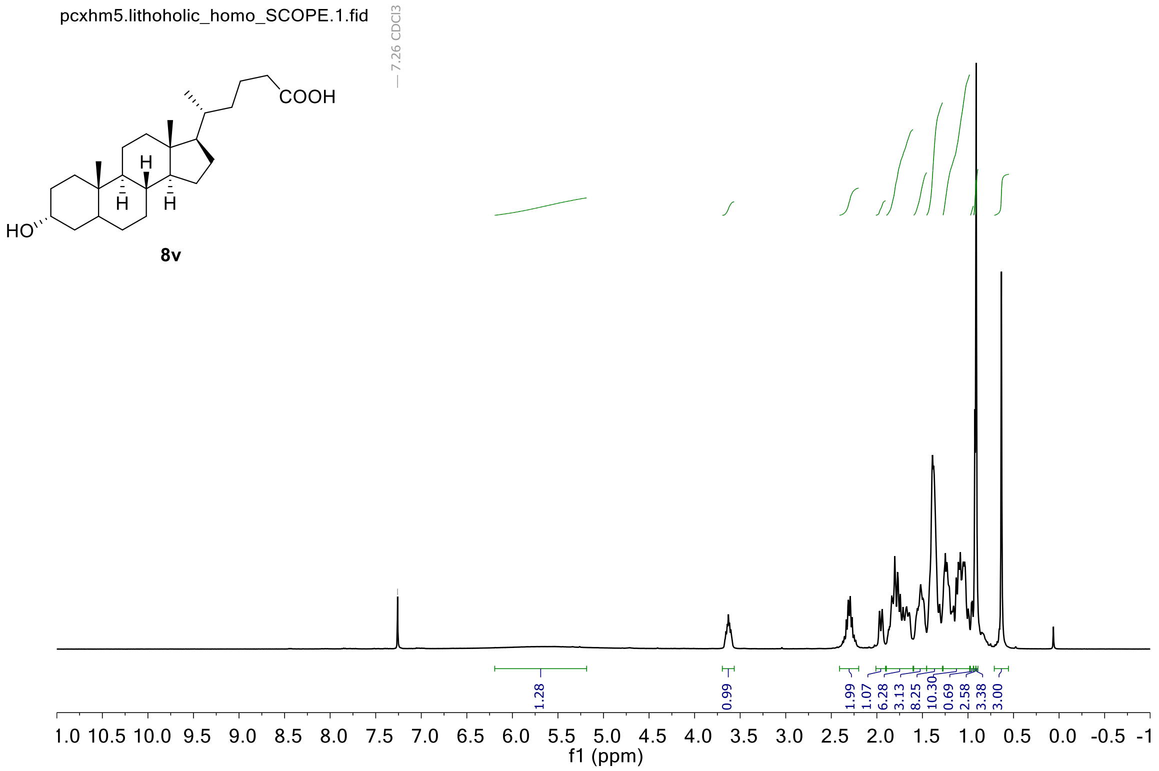

^{13}C-NMR ($CDCl_3$, 101 MHz)

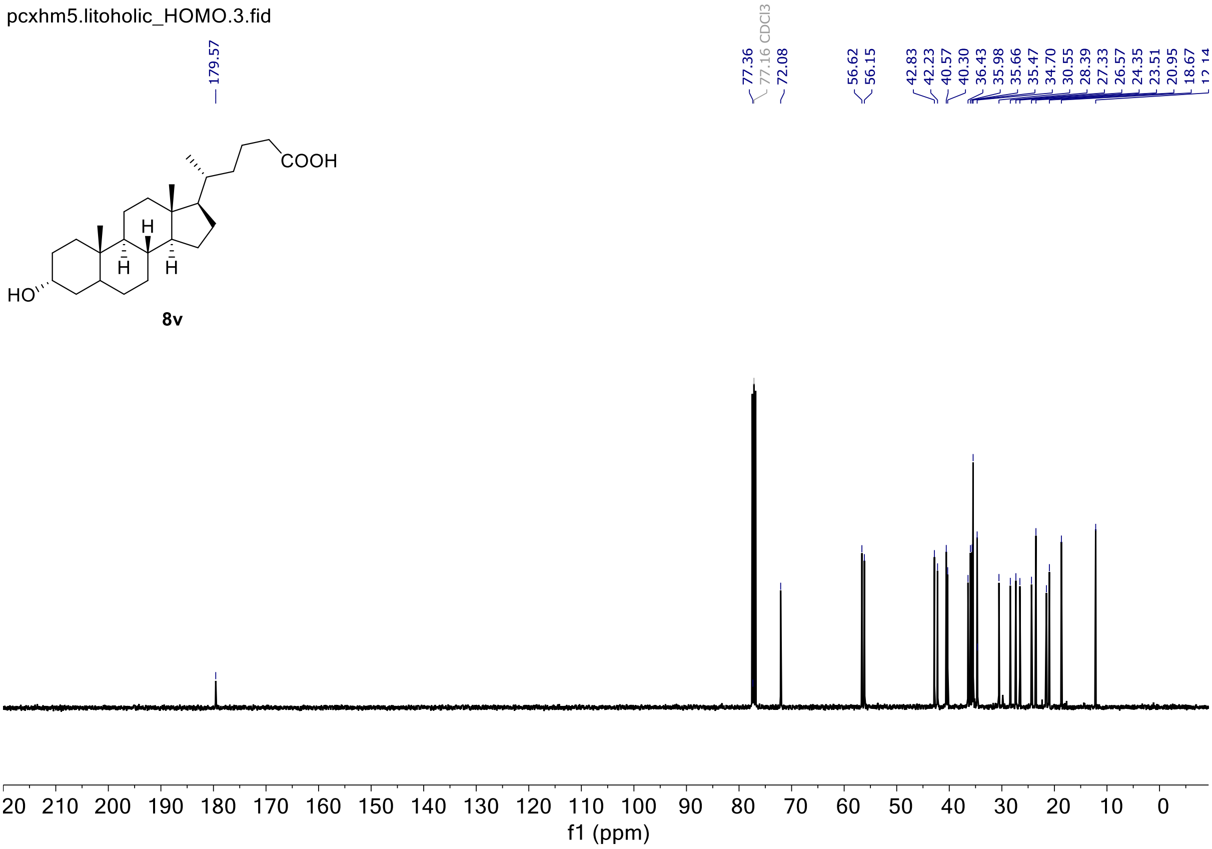

2-((4aR,6aS,6bR,8aR,10S,12aR,12bR,14bR)-10-hydroxy-2,2,6a,6b,9,9,12a-heptamethyl-1,3,4,5,6,6a,6b,7,8,8a,9,10,11,12,12a,12b,13,14b-octadecahydropicen-4a(2H)-yl)acetic acid (8w)

^{1}H-NMR ($CDCl_3$, 500 MHz)

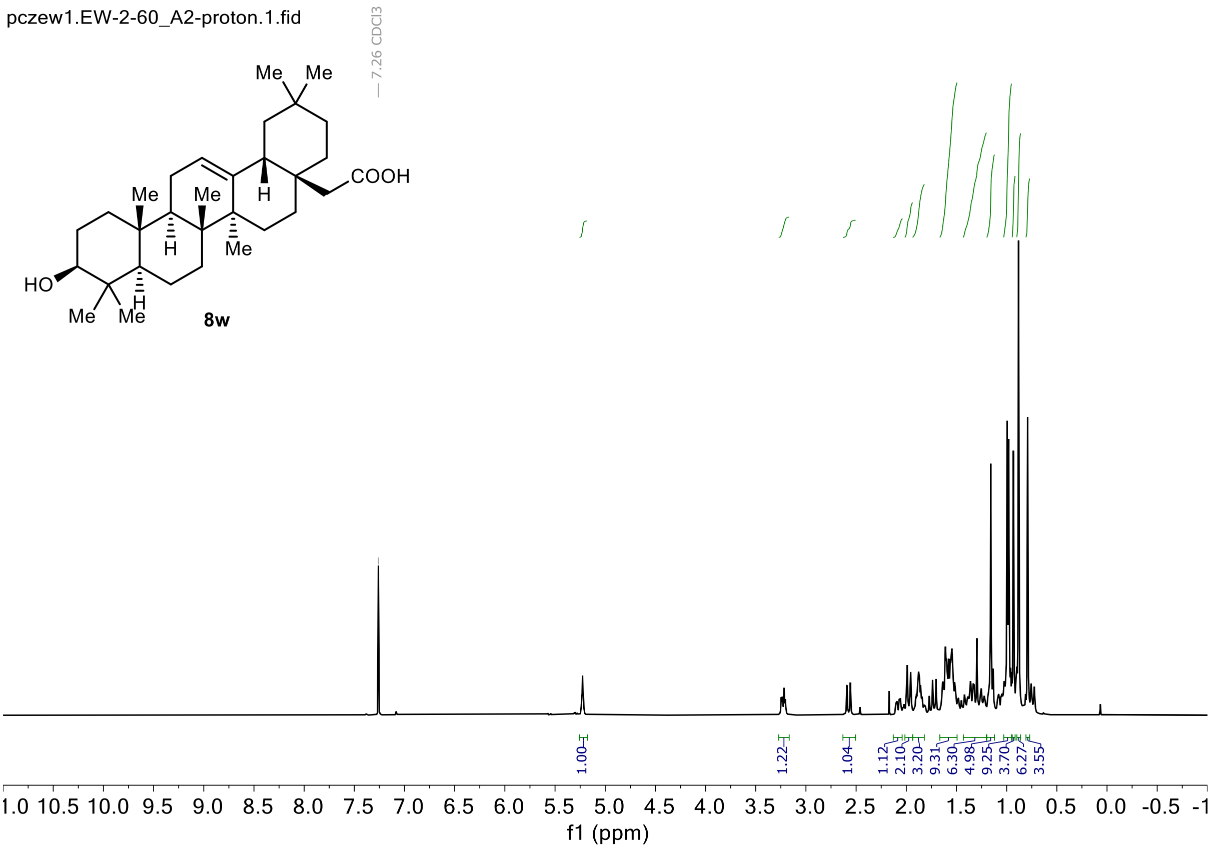

^{13}C-NMR ($CDCl_3$, 126 MHz)

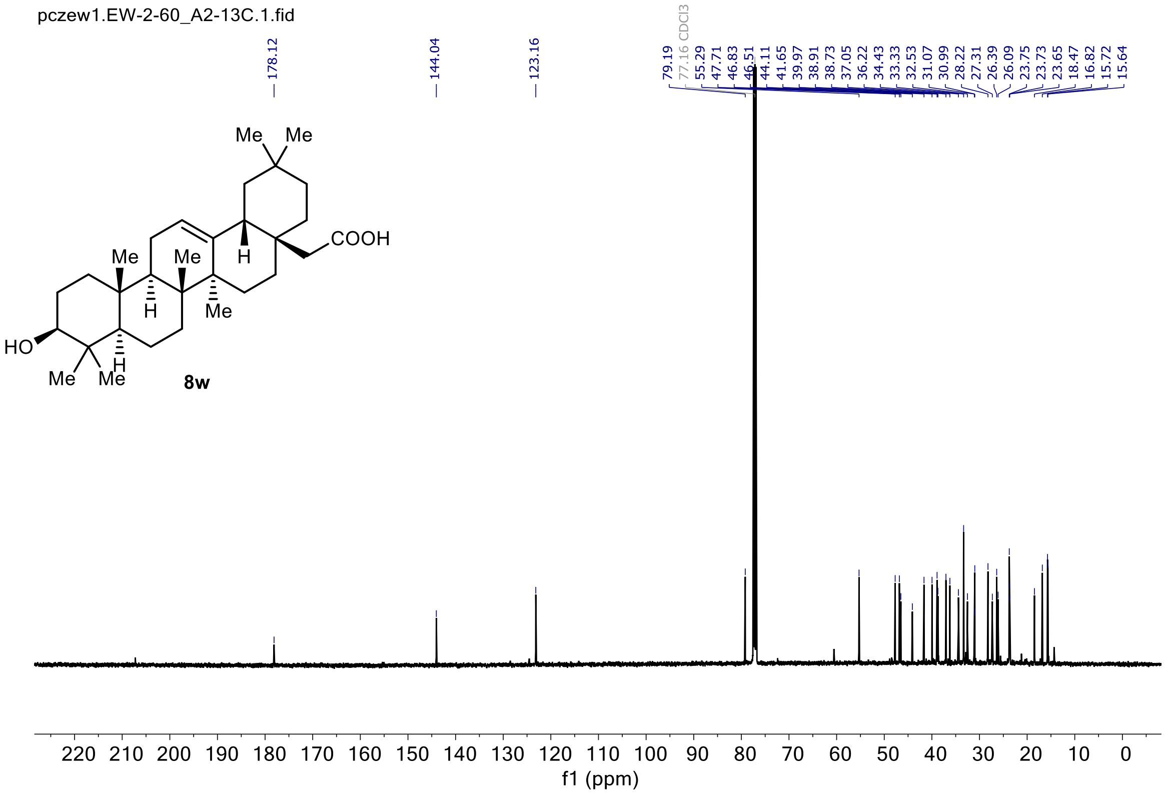

(S)-6-(tert-butoxy)-5-((tert-butoxycarbonyl)amino)-6-oxohexanoic acid (8x)

^{1}H-NMR ($CDCl_3$, 400 MHz)

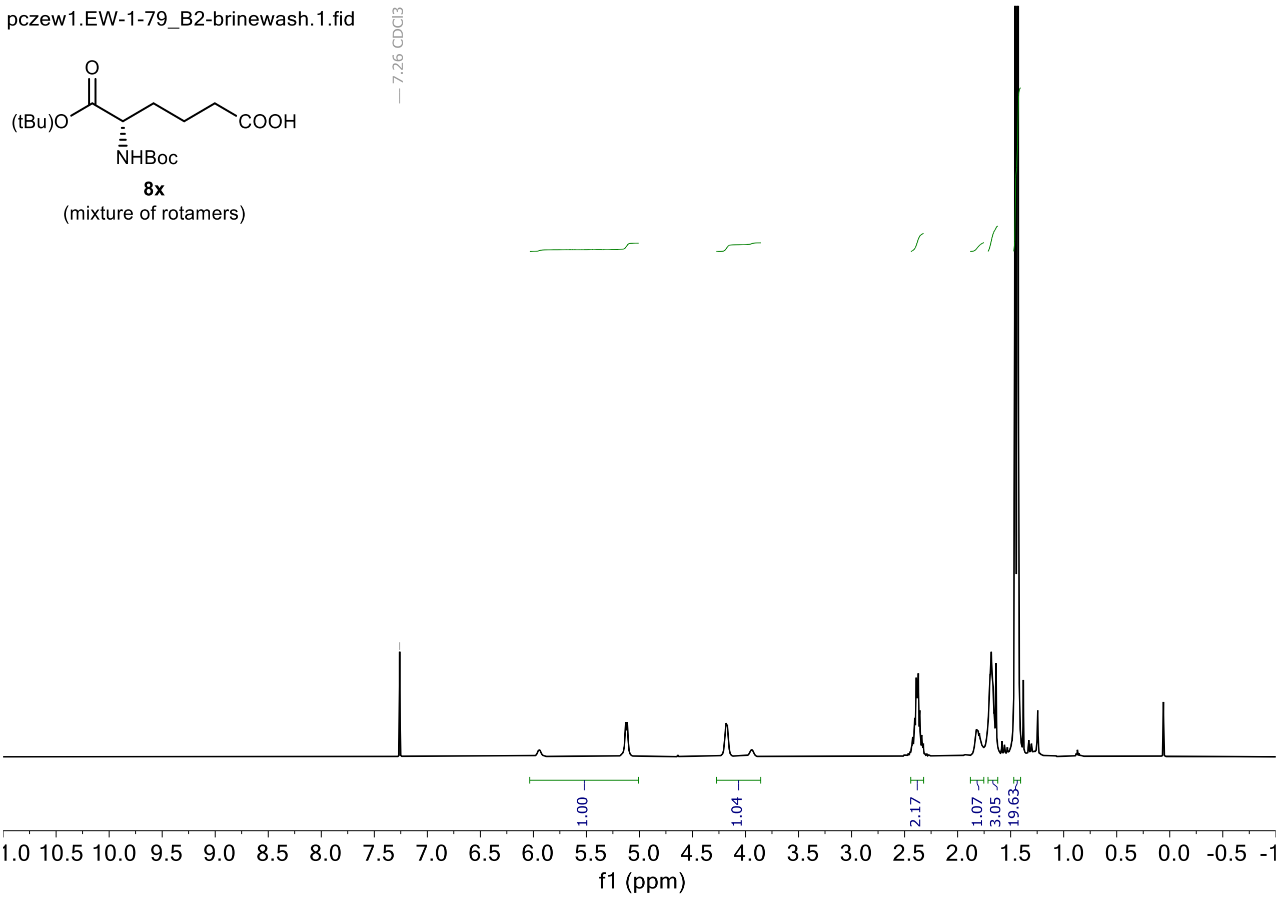

^{13}C-NMR ($CDCl_3$, 101 MHz)

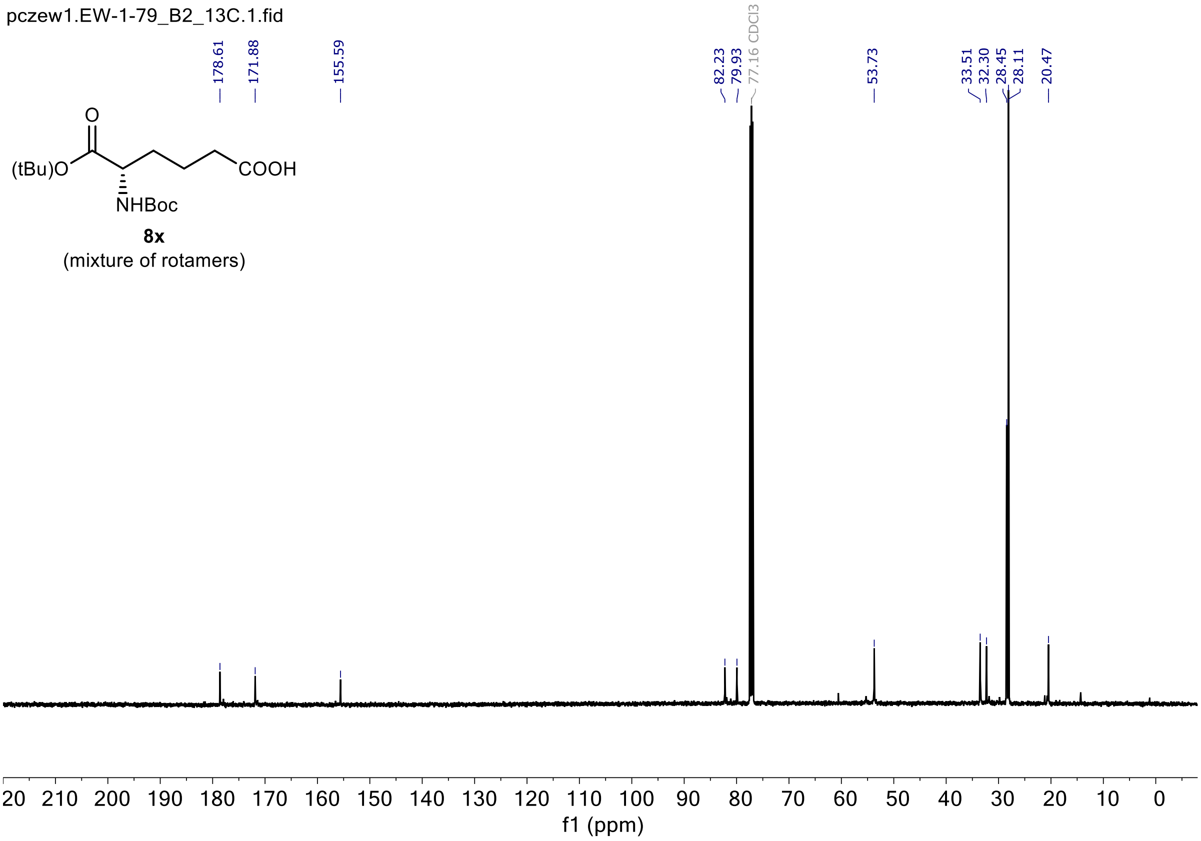

(S)-7-(tert-butoxy)-6-((tert-butoxycarbonyl)amino)-7-oxoheptanoic acid (8y)

^{1}H-NMR ($CDCl_3$, 400 MHz)

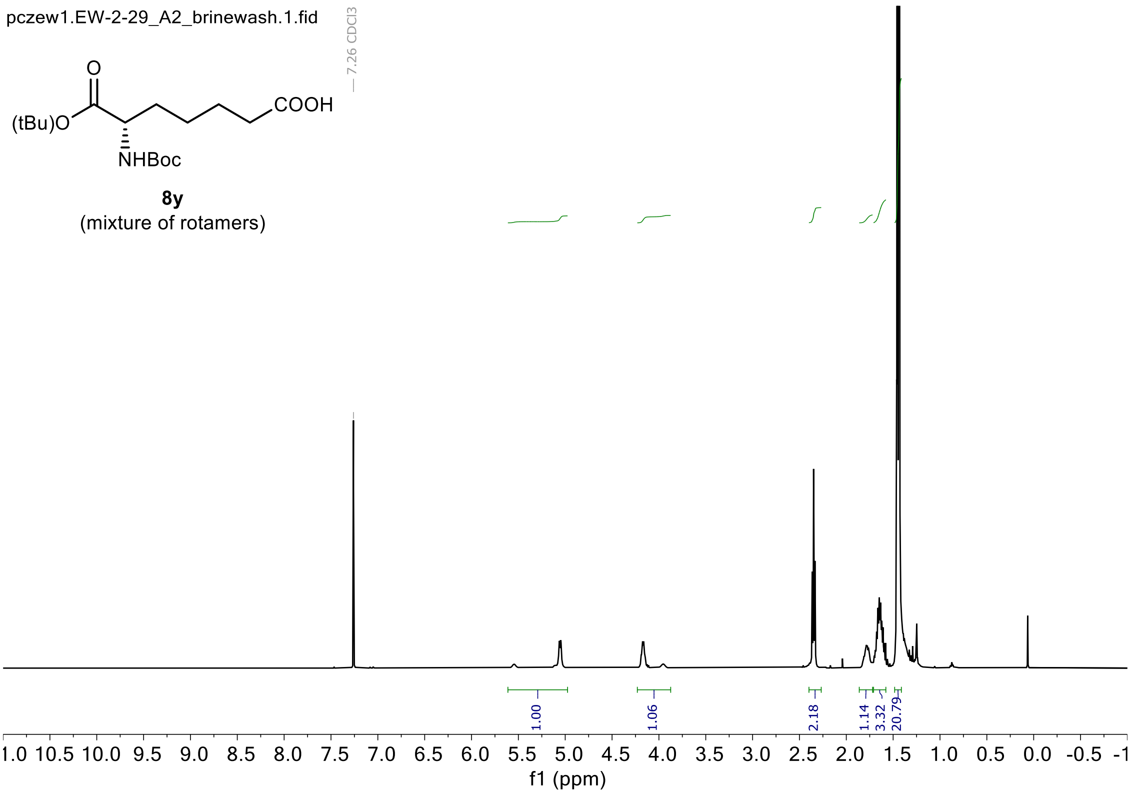

8y
(mixture of rotamers)

^{13}C-NMR ($CDCl_3$, 101) MHz

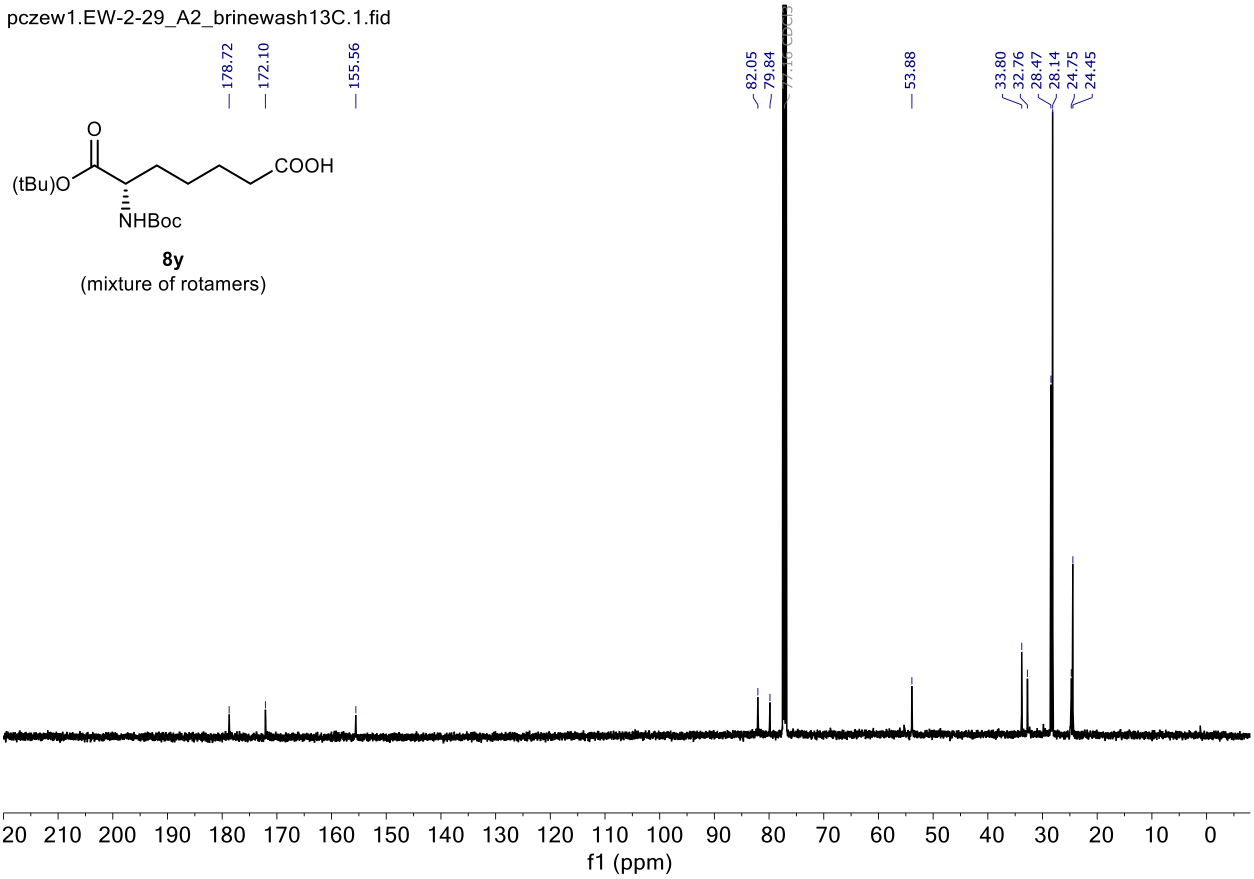

8y
(mixture of rotamers)

tert-butyl (S)-6-amino-2-((tert-butoxycarbonyl)amino)-6-oxohexanoate (9)

^{1}H-NMR ($CDCl_3$, 500 MHz)

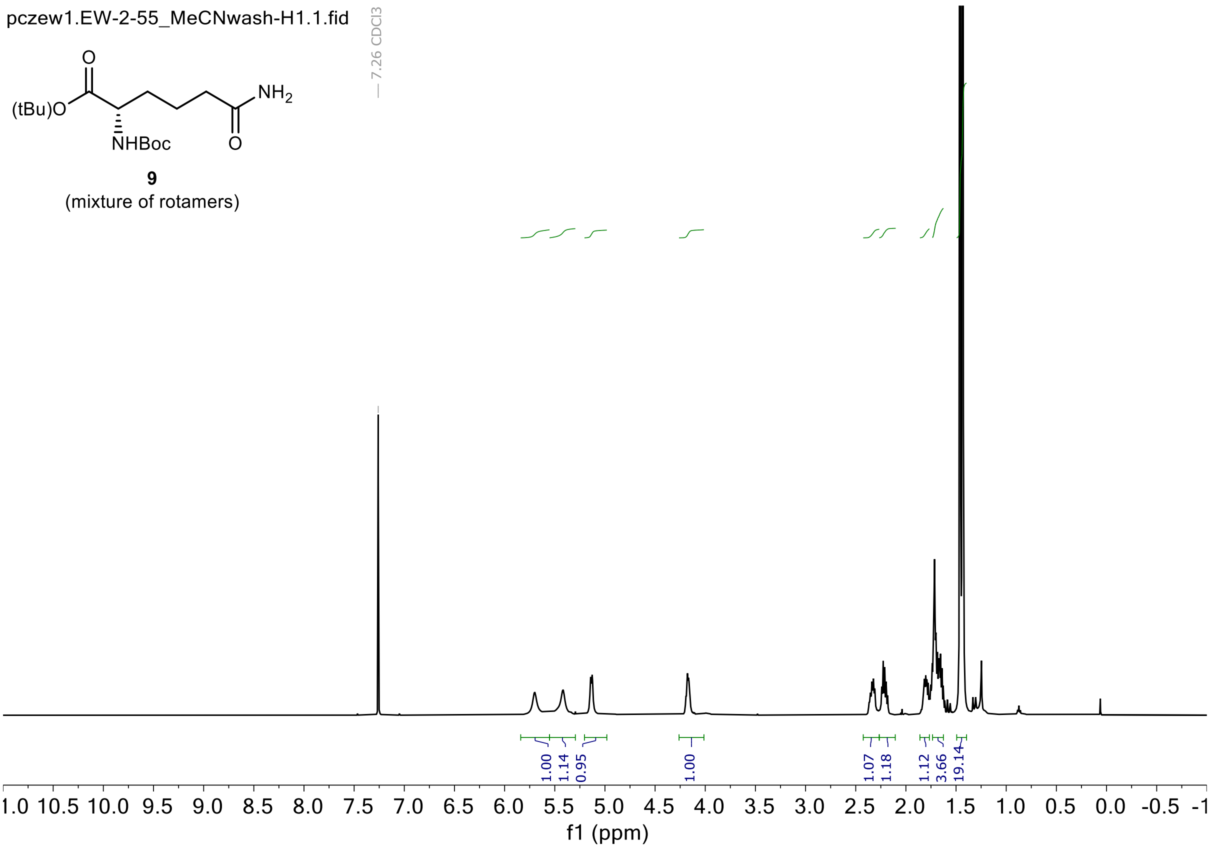

^{13}C-NMR ($CDCl_3$, 126 MHz)

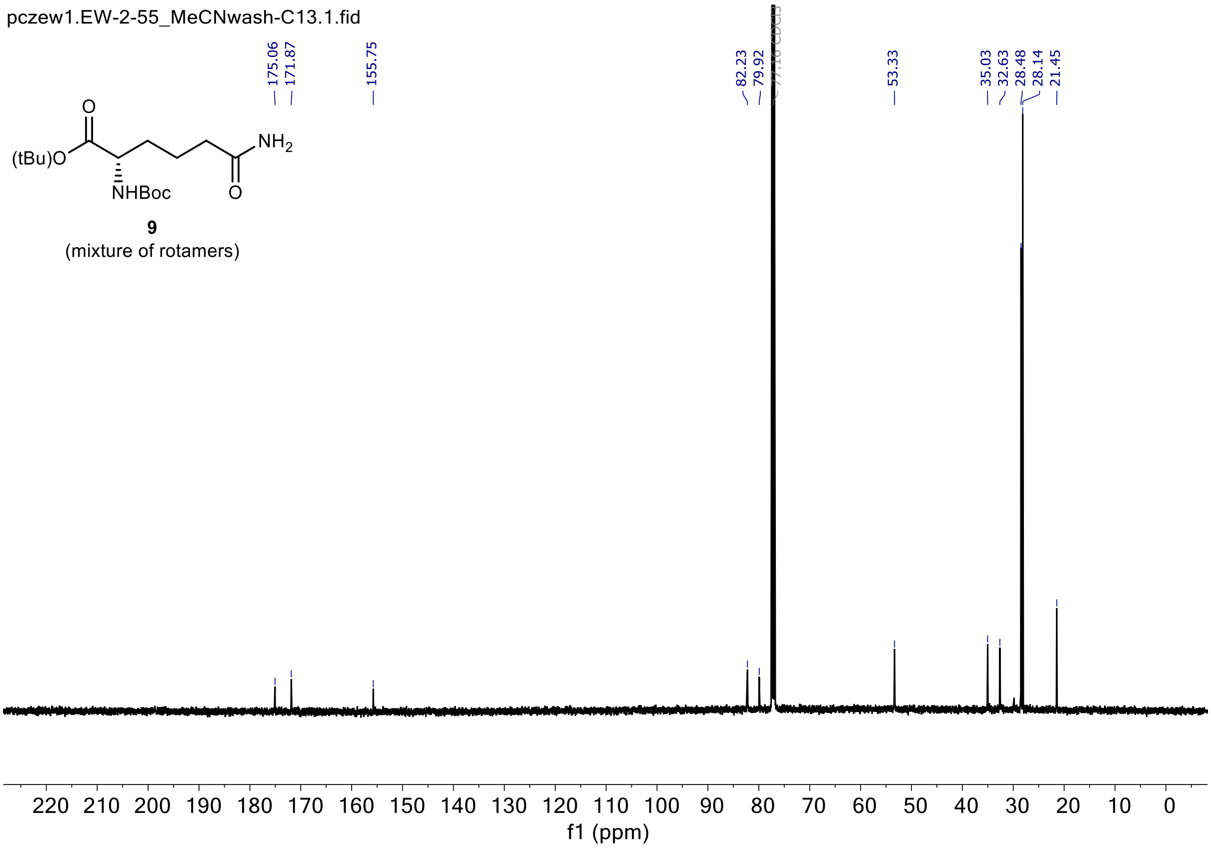

tert-butyl (S)-2-((tert-butoxycarbonyl)amino)-6-hydroxyhexanoate (10)

^{1}H-NMR ($CDCl_3$, 500 MHz)

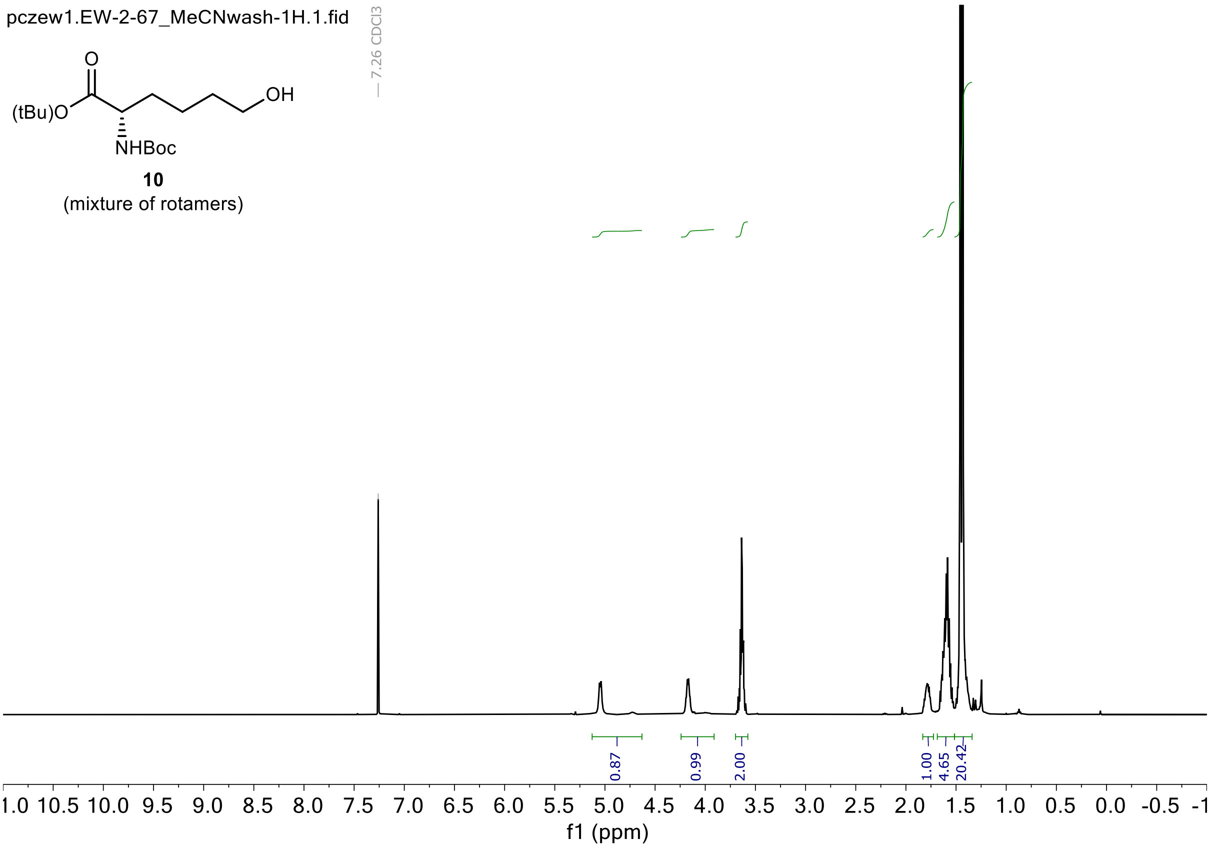

^{13}C-NMR ($CDCl_3$, 126 MHz)

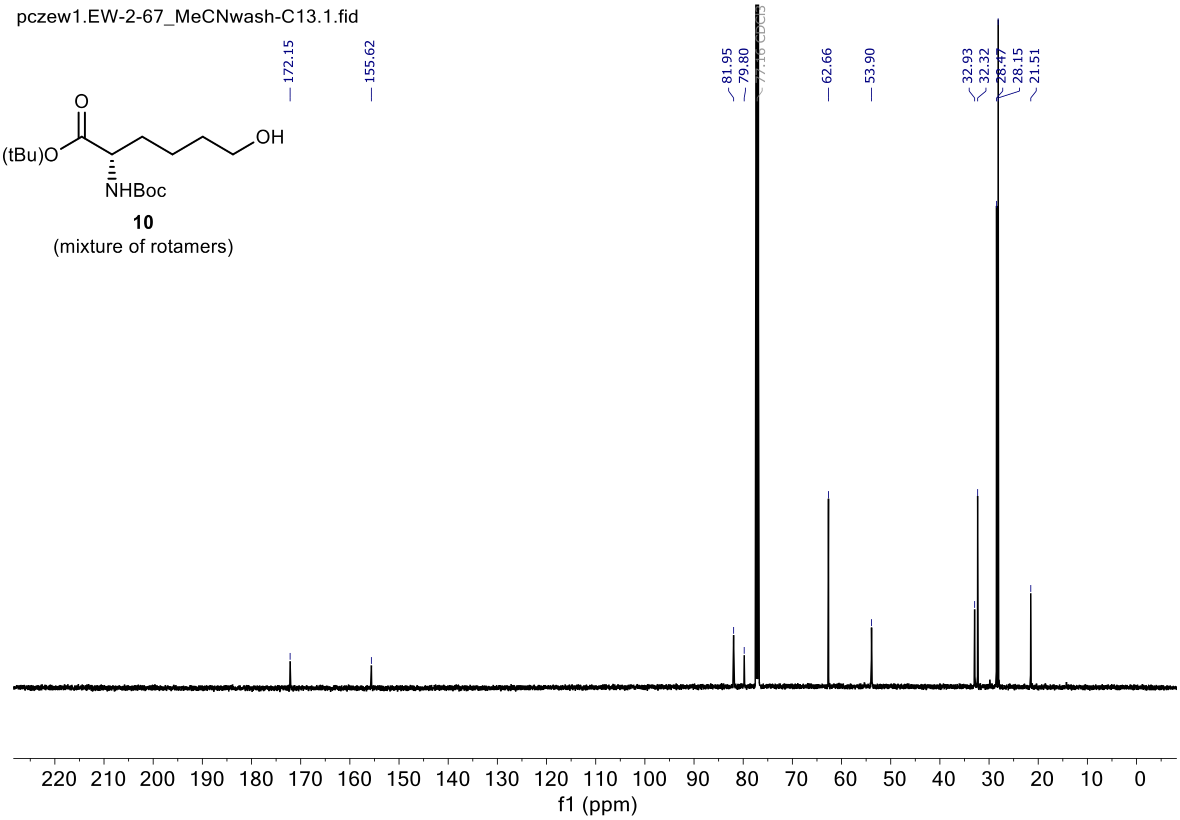

tert-butyl (S)-7-amino-2-((tert-butoxycarbonyl)amino)heptanoate (11)

^{1}H-NMR (CDCl$_3$, 500 MHz)

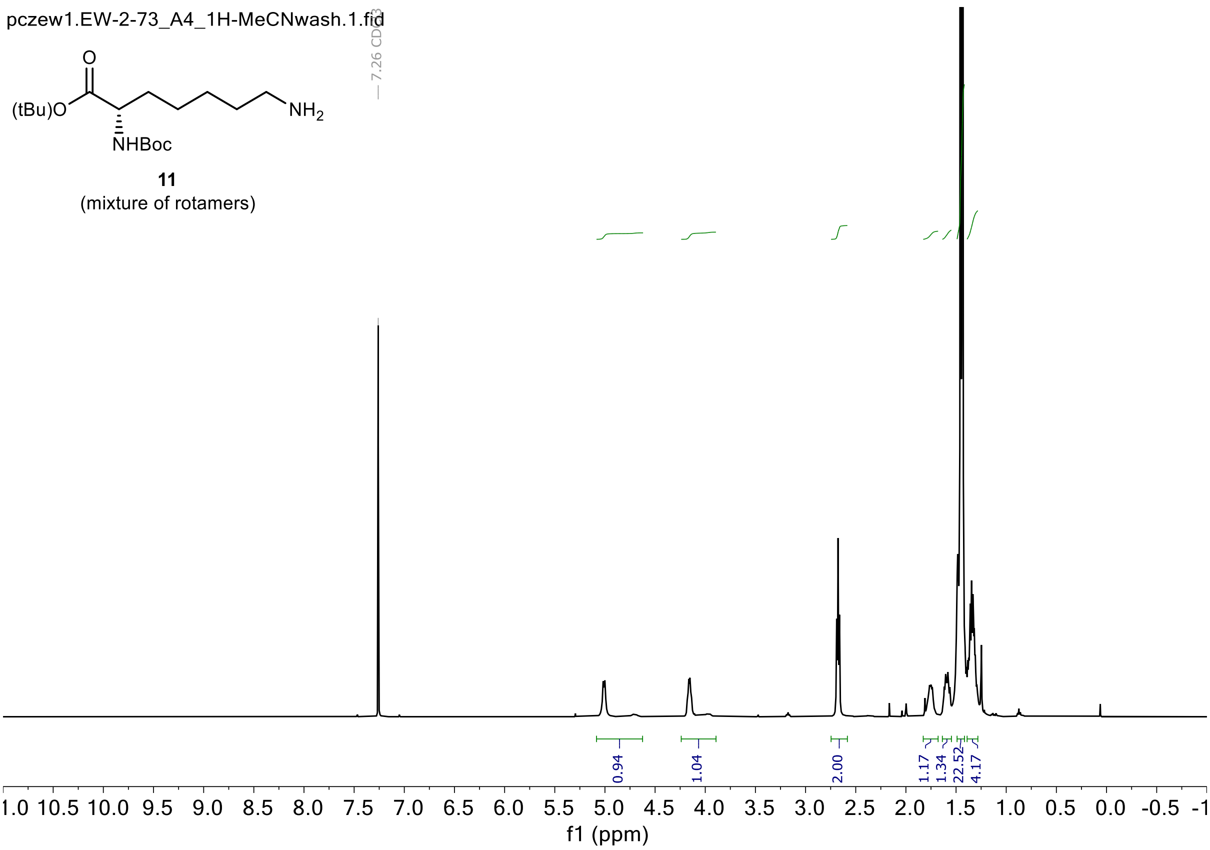

^{13}C-NMR (CDCl$_3$, 126 MHz)

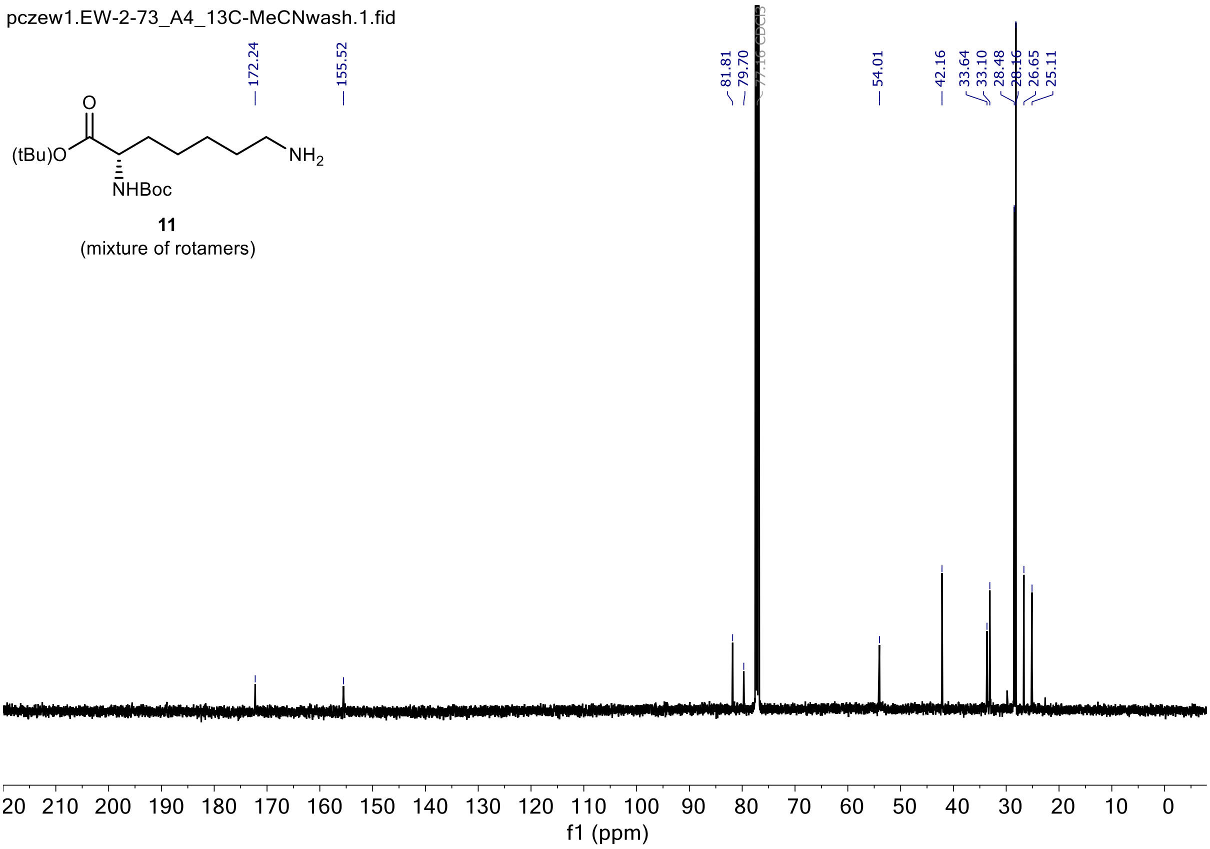

2-((1S,4aS)-7-isopropyl-1,4a-dimethyl-1,2,3,4,4a,9,10,10a-octahydrophenanthren-1-yl)acetic acid (8z)

^{1}H-NMR ($CDCl_3$, 500 MHz)

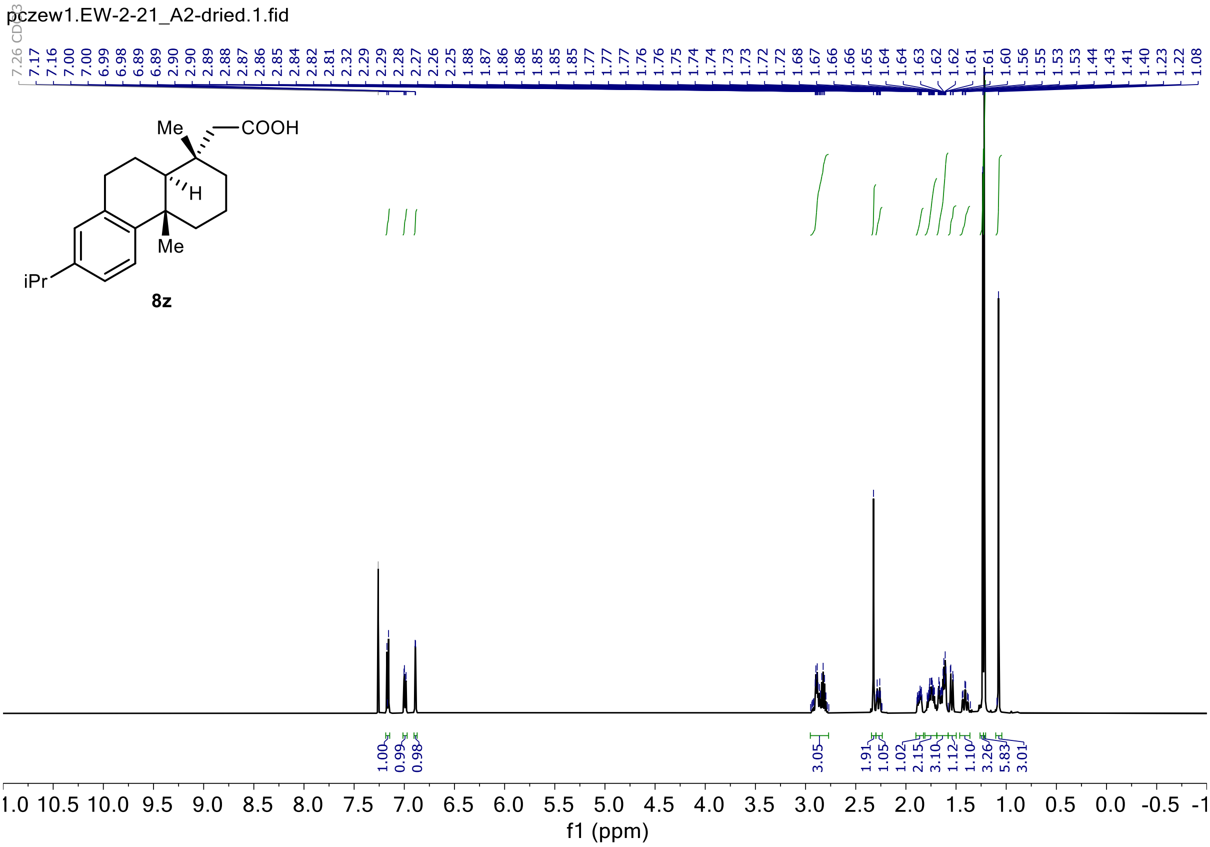

^{13}C-NMR ($CDCl_3$, 126 MHz)

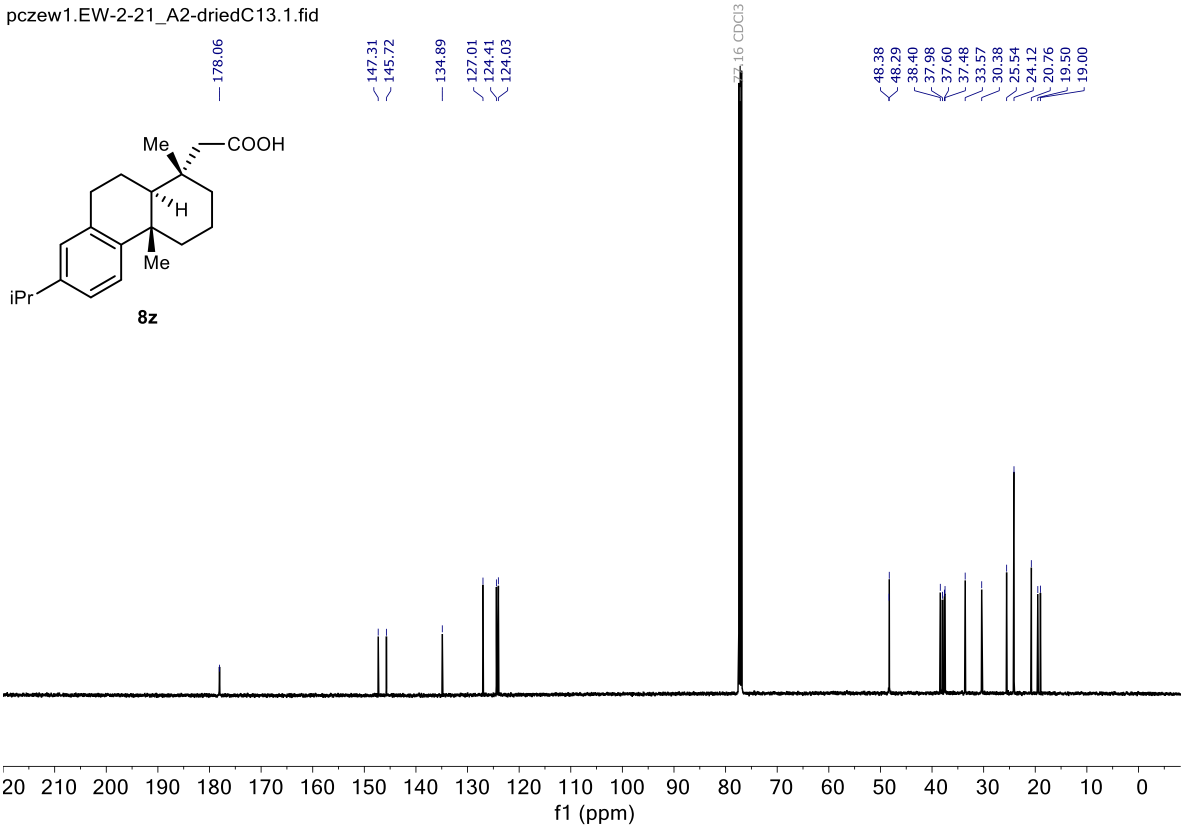

2-((1S,4aS)-7-isopropyl-1,4a-dimethyl-1,2,3,4,4a,9,10,10a-octahydrophenanthren-1-yl)ethan-1-amine (8x)

^{1}H-NMR (CDCl$_3$, 400 MHz)

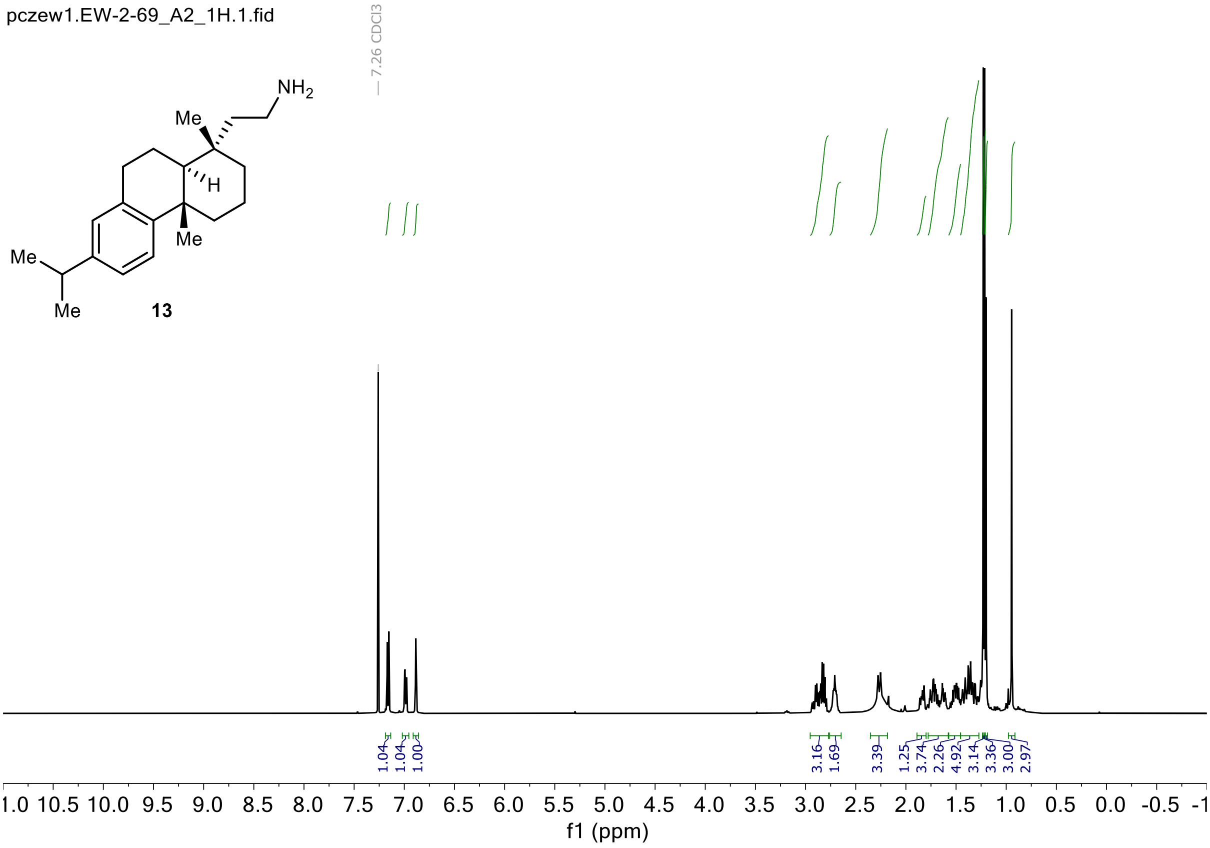

^{13}C-NMR (CDCl$_3$, 101 MHz)

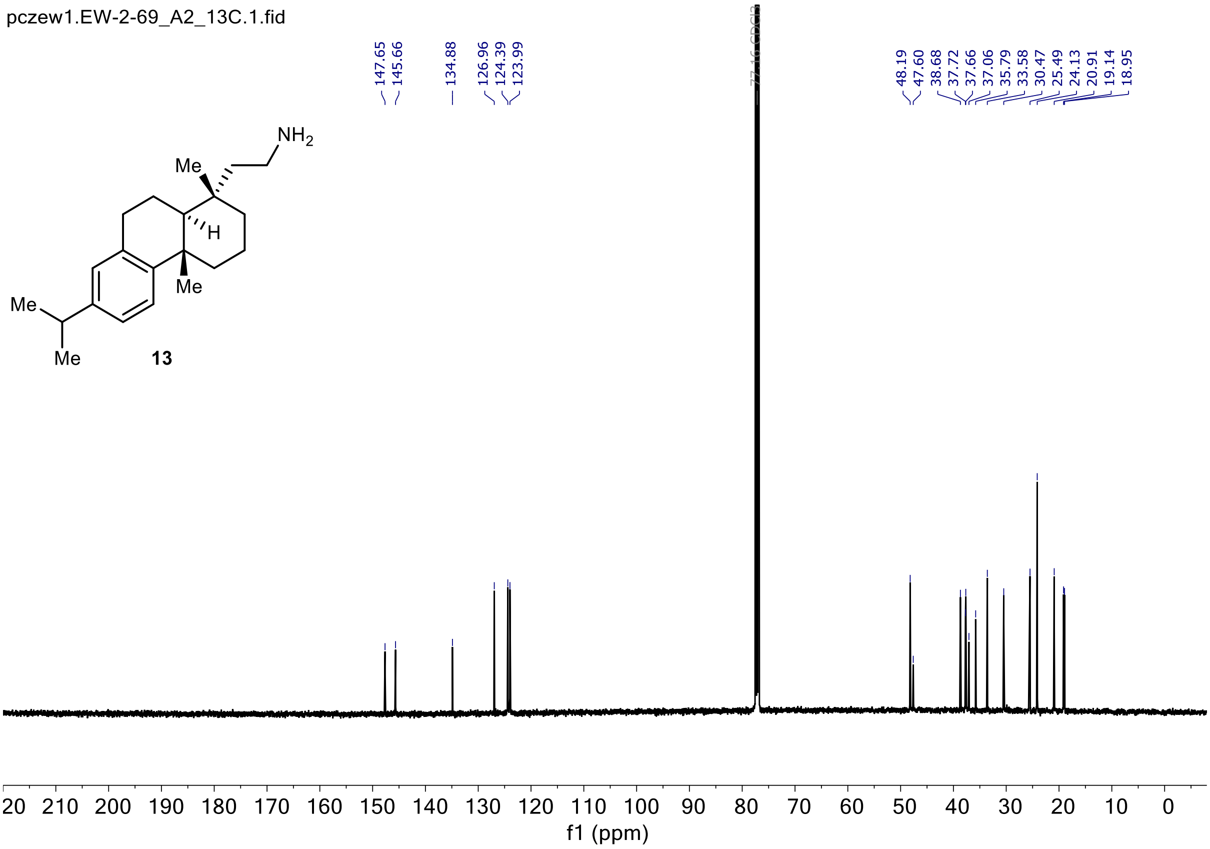

Supplement: Supplementary file 1 — ja4c13630_si_001.pdf [file ja4c13630_si_001.pdf]
